# Liquid-Phase CO**_2_** Capture by a Nonaqueous Cooperative Absorption Mechanism

**DOI:** 10.1021/jacs.6c06590

**Published:** 2026-06-04

**Authors:** Lu Lu, Ankana Roy, Saptarshi Chatterjee, Stephen Schuyten, M. G. Finn, Ryan P. Lively

**Affiliations:** † School of Chemical & Biomolecular Engineering, 1372 Georgia Institute of Technology, Atlanta, Georgia 30332, United States; ‡ School of Chemistry and Biochemistry, Georgia Institute of Technology, Atlanta, Georgia 30332, United States; § School of Biological Sciences, 1757 Virginia Tech, Blacksburg, Virginia 24061, United States; ∥ Johnson Matthey Process Technologies Inc., Savannah, Georgia 31408, United States

## Abstract

Anthropogenic carbon dioxide emissions remain a critical driver of climate change, necessitating the development of efficient carbon capture technologies. While aqueous amines are widely used, they suffer from high regeneration energies and solvent degradation. Here, we report a nonaqueous, cooperative CO_2_ absorption system using 1-methylpiperazine (MPZ) dissolved in a family of aromatic additives. Among these, the formulation with 2′-hydroxyacetophenone (2′HAP) exhibited the best overall performance, with a stepped sorption isotherm indicating cooperative absorption and a CO_2_:MPZ stoichiometry of up to 0.93 at moderate pressures (∼163 kPa) without the addition of water. While other structurally related additives, including acetophenone (AP), 2′-methoxyacetophenone (2′MAP), and 1,4-diisopropylbenzene (DIPB), also enabled high uptake capacities (absorbed CO_2_:MPZ stoichiometries up to ∼0.85), the stepped behavior enables enhanced working capacity with reduced temperature differentials, offering potential energy savings in temperature-swing absorption processes. Measurements of performance with different additives including acetophenone isomers and phenolic analogs revealed that CO_2_ uptake and isotherm shape are governed by solvent acidity, carbonyl presence, and the ability to stabilize carbamic acid intermediates, perhaps through hydrophobic interactions. Equilibrium network modeling supported the proposed mechanism, illustrating how additive-amine interactions shift key equilibria to enable stepped isotherm behavior. Control experiments with monoethanolamine (MEA) and morpholine (MP) underscored the importance of the diamine nature of MPZ in facilitating cooperative uptake. Breakthrough experiments confirmed the system’s robust performance across varying CO_2_ concentrations (4.5–25%). This work provides mechanistic insights into additive-amine cooperativity and highlights MPZ-based nonaqueous systems as promising candidates for energy-efficient CO_2_ capture.

## Graphical abstract

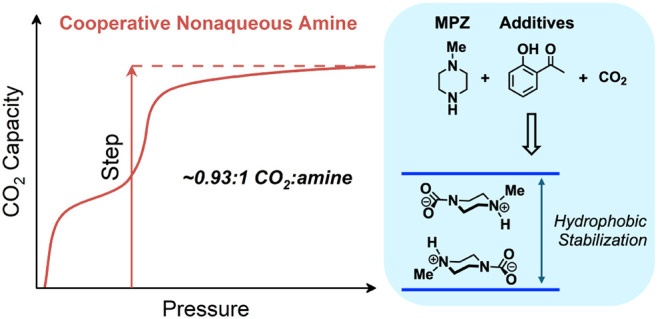

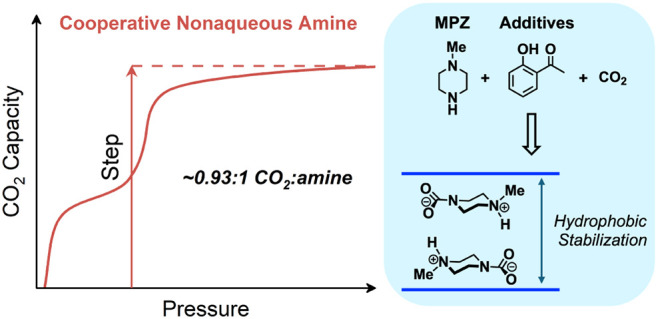

## Introduction

Escalating levels of anthropogenic carbon dioxide (CO_2_) in the atmosphere are a critical driver of global climate change. Combustion of fossil fuels in power plants and industrial processes accounts for approximately 80% of global CO_2_ emissions.^1^ Despite efforts to transition to cleaner energy, fossil fuels are expected to remain a dominant energy source for the foreseeable future. Consequently, carbon capture and sequestration (CCS) have emerged as an essential strategy to mitigate CO_2_ emissions. While postcombustion CCS targets flue gas containing 4–15% CO_2_,^2–4^ the growing demand for hydrogen as a clean energy carrier further underscores the importance of advancing CO_2_ separation technologies.^5,6^ Blue hydrogen, produced via steam methane reforming (SMR) with CCS, offers a low-carbon pathway for meeting the growing hydrogen demand. The SMR process generates CO_2_ concentrations of ∼15–25% in shifted syngas, which enables more efficient CO_2_ capture compared to flue gas. However, the costs of CO_2_ capture, transportation, and storage remain significant challenges.^7,8^

The most well-understood process for CO_2_ treatment is the absorber-stripper-based approach. Both physical and chemical solvents are commonly employed, which differ in their modes of interaction and resulting energy requirements. Physical solvents absorb CO_2_ through dissolution and relatively weak molecular interactions, which can enable energy-efficient desorption in the stripping process. This method has been widely applied in industrial processes to separate high-activity/pressure gas mixtures, with several such processes successfully commercialized.^9–12^ In contrast, chemical solvents form stronger chemical bonds with the target gas and have been extensively studied for their ability to extract CO_2_ from low pressure sources.^13–15^ Aqueous solutions of amines such as monoethanolamine (MEA) are among the most widely employed sorbents due to their ability to selectively and rapidly react with CO_2_.^16,17^ However, aqueous amine solutions are also subject to solvent evaporation, amine oxidation, and high regeneration energy requirements. Recent research on water-lean amine systems^18^ and novel separation media, such as porous liquids,^19^ offers potential for more efficient CO_2_ capture. However, large-scale testing for CO_2_ separation using these systems remains limited.^20,21^

In our efforts to develop porous liquids as solvents for CO_2_ separation,^22–24^ we incorporated amines such as piperazine (PZ) and derivatives to enhance sorption capacity in the low-pressure regime relevant to postcombustion conditions. Control experiments using only the amine dissolved in one of the commonly used porous liquid solvents (2′-hydroxyacetophenone, 2′HAP) revealed unexpected CO_2_ sorption and desorption behavior without the presence of porous cages. We describe here the use of 1-methylpiperazine (MPZ), chosen because of its liquid state at ambient temperature, lower heat of absorption, and faster reaction kinetics compared to MEA and PZ.^13,25,26^ MPZ mixed with 2′HAP gave a “stepped” CO_2_ sorption isotherm, which suggested the existence of a cooperative absorption phenomenon. Such cooperativity presents promising opportunities for enhancing working capacity while reducing the need for large temperature and pressure swings in absorption–desorption cycles.^27–29^

Figure 1 shows the various components employed in our exploration of the CO_2_ uptake behavior of the solution-phase MPZ + HAP system. Monoethanolamine (MEA) and morpholine (MP) were used to test the extension of cooperative absorption phenomena to other amines. In addition to structurally related analogs of 2′HAP [acetophenone (AP), 3′-hydroxyacetophenone (3′HAP), 4′-hydroxyacetophenone (4′HAP)], two additional porous liquid solvents [2-isopropylphenol (2IPP) and 2-chlorophenol (2CP)] were also tested. Two additional solvents, 2′-methoxyacetophenone (2′MAP) and 1,4-diisopropylbenzene (DIPB), served as controls for the hydroxyl and ketone groups of 2′HAP and for simple steric effects. While 2′HAP, 2IPP, and 2CP have been used in many porous liquids,^22,23,30^ these findings provide a comprehensive evaluation of the use of MPZ with a variety of water-lean solvents for CO_2_ capture, offering new insights into the potential of cooperative absorption mechanisms for optimizing carbon capture processes.

**Figure 1:**
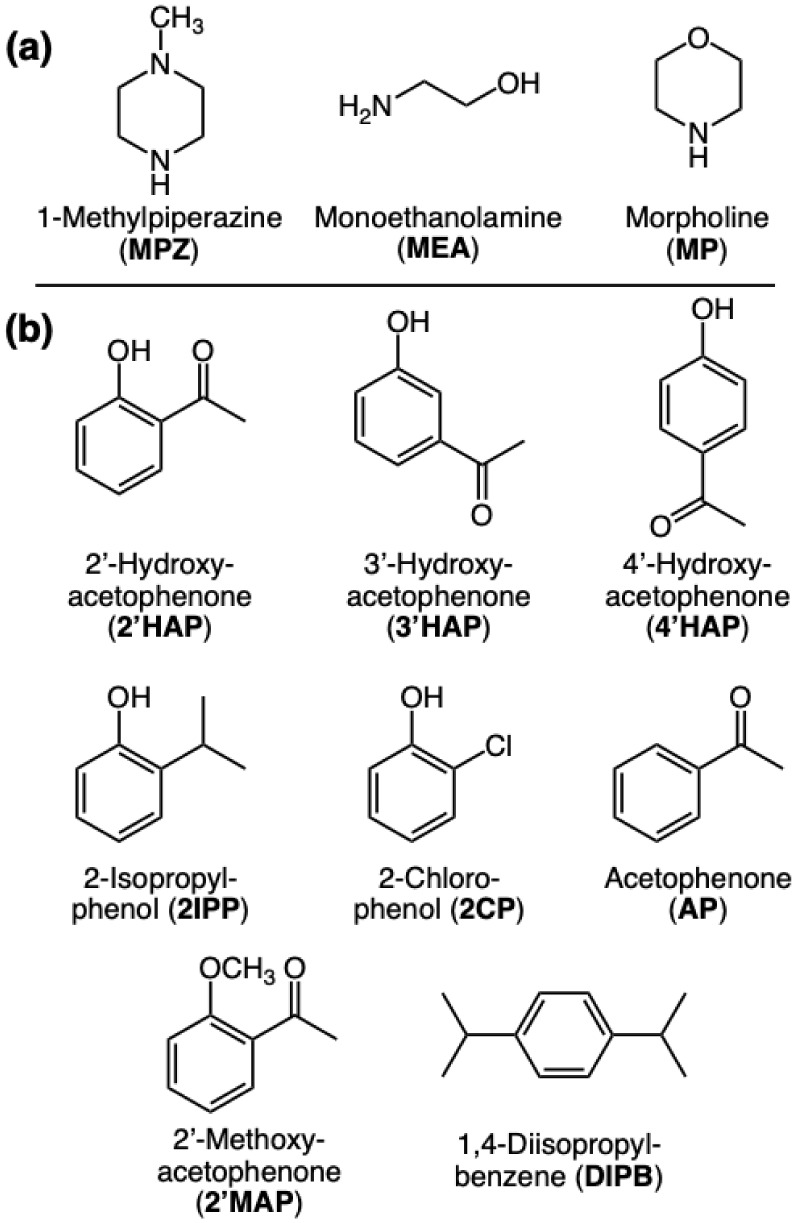
Chemical structures of the (a) amines and (b) solvents used in this study. These solvents are also referred to here as “additives” since they are not often used in large molar excess relative to amines.

## Methods

### Materials

Commercially available reagents were of reagent grade or better and used as received. The solvents used for syntheses and washings were of analytical grade. 1-Methylpiperazine, monoethanolamine, morpholine, 2′-hydroxyacetophenone, 3′-hydroxyacetophenone, 4′-hydroxyacetophenone, 2-isopropylphenol, 2-chlorophenol, acetophenone, 2′-methoxyacetophenone and 1,4-diisopropylbenzene were purchased Sigma-Aldrich. Ultrahigh purity He, N_2_, pure CO_2_ and premixed 4.5%, 12.5%, and 25% CO_2_ with He/N_2_ were purchased from Airgas and used as received.

### CO_2_ Isotherms

An in-house pressure decay cell described in the literature^24^ was used to measure CO_2_ isotherms. The sample and dosing chamber volumes were calibrated beforehand. Liquid samples were purged with helium for 2 h to eliminate absorbed CO_2_ and other gases. After degassing, CO_2_ was injected into the dosing chamber, evacuated three times to remove residual gases, and then introduced at the desired pressure. The valve between the dosing and sample chambers was opened briefly, and pressure decay was monitored for 6–24 h until equilibrium was reached. This process was repeated at increasing pressures up to 100–200 kPa.

### ^13^CO_2_ Absorption Measurement

1 mL freshly made solutions of MPZ in AP, 2′HAP, 3′HAP, and 4′HAP were transferred into four 25 mL flasks and connected to a Schlenk line. Each of the reaction vessels was degassed by three freeze–pump–thaw cycles, backfilling with N_2_. After a final pump-down in a dry ice-acetone bath, ^13^CO_2_ was introduced to the evacuated system from a gas cylinder (0.461 L volume, initial pressure = 2.37 atm). A brass CGA 110/180 regulator was used to maintain the system at ∼1 atm of ^13^CO_2_ pressure. The reaction vessel was then warmed to 30 °C (oil bath), and the system was allowed to stand overnight to allow ^13^CO_2_ absorption by the MPZ solutions. After absorption, the contents of each vessel consisted of both liquid and a solid or gel-like phase, indicating partial precipitation or phase separation. The mixtures (including both solid and liquid components) were therefore scooped out and dissolved in an appropriate NMR solvent for subsequent analysis. This experiment was designed to identify the species formed upon CO_2_ absorption, rather than to quantify their relative or absolute amounts.

### NMR

Spectra recorded in CDCl_3_ with a Bruker Avance 500 MHz NMR spectrometer. Chemical shifts are reported in ppm (δ) with reference to internal residual protonated species of the deuterated solvent.

### Dynamic Breakthrough Experiments

Liquid-phase breakthrough measurements were performed in a bubbler reactor under three inlet gas compositions: 4.5 mol % CO_2_ + 4.5 mol % He + balance N_2_, 12.5 mol % CO_2_ + 12.5 mol % He + balance N_2_, and 25 mol % CO_2_ + balance He (Figure S13). The bubbler reactor was made of cylindrical graduated glass (2.5 cm radius) with removable hose connections; the bubbler nozzle was equipped with a sintered glass filter for even gas distribution. Gas flow was controlled using a mass flow controller (Alicat Scientific/Omega), and temperature was controlled by an oil bath. Approximately 10 g of MPZ/solvent solution was loaded into the bubbler, and glass wool and a cold trap were placed at the gas outlet to prevent solvent carryover before the gas entered the CO_2_ analyzer. Sample activation was conducted at 60 °C under dry N_2_ flow for 2 h and then cooled to the set absorption temperature. The inlet gas stream was then switched to 4.5%, 12.5%, or 25% CO_2_, initiating the CO_2_ absorption process. The outlet CO_2_ concentration was continuously monitored using an infrared gas analyzer (Quantek Model 906). Once the outlet CO_2_ concentration reached the inlet concentration, the gas flow was switched to pure N_2_, and the reactor was purged at the absorption temperature for 1–2 h to desorb weakly bound CO_2._ Additional desorption was carried out at 60, 80, and 100 °C under an inert gas flow with continuous CO_2_ monitoring. A blank experiment (carried out with an empty reactor) was conducted to determine system dead time.

## Results and Discussion

### CO_2_ Solubility Measurements

The reaction of primary and secondary amines with CO_2_ in aqueous solutions is a benchmark for point source CO_2_ capture.^31–33^ The rapid formation of a stable ammonium carbamate (reaction 1) imposes a stoichiometric limit of 0.5 mol CO_2_ per mol of amine. While the presence of water allows for carbamate hydrolysis to bicarbonate (reaction 2) and therefore the possibility of the association of 1 mol CO_2_ per mol amine (reaction 3), such a process is often thermodynamically unfavorable due to the high stability of the carbamate-ammonium ion pair and carbamate hydrolysis is kinetically slow compared to the rapid initial carbamate formation. This behavior typifies first-generation aqueous amine solvents such as MEA, widely employed in commercial CO_2_ capture processes.^34,35^ 
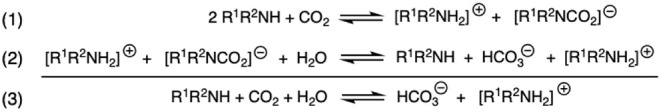


The pressure-dependent absorption of CO_2_ by MPZ in aqueous solution has been previously described.^36^ Consistent with that report, we found a 40 weight-percent (wt %) aqueous MPZ solution to absorb 3.92 mmol CO_2_ per gram solution (1.09 mol CO_2_ per mol MPZ) at 175 kPa (Figure S1), presumably because of its diamine structure. The secondary amine initially forms a carbamate, while the adjacent tertiary amine, incapable of direct carbamate formation, acts as an internal base to provide an approximately 1:1 CO_2_:MPZ stoichiometry.^37^ In the presence of water, marginal gains in CO_2_ uptake per mole of MPZ likely occur by virtue of aqueous bicarbonate formation and regeneration of the secondary amine (Scheme 1).^37^

**Scheme 1:**
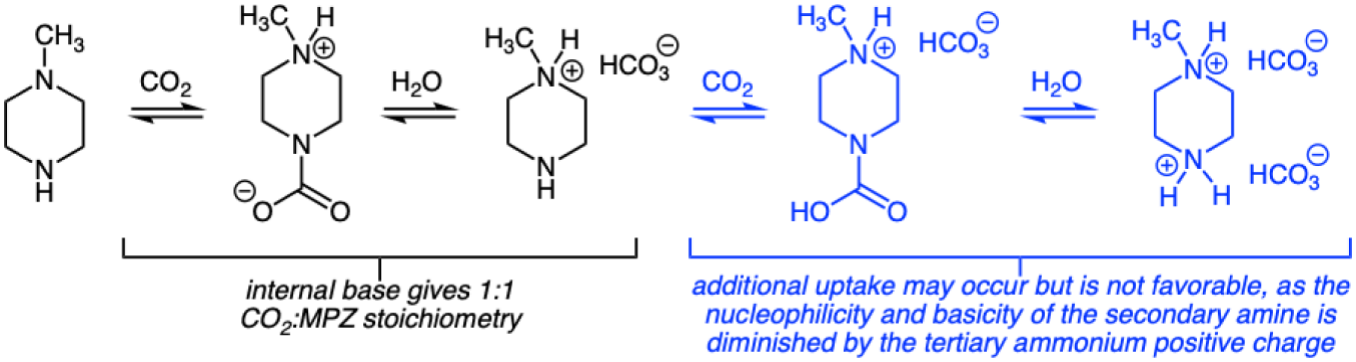
Steps in the Interaction of MPZ with CO_2_ and Water

While aqueous amines benefit from higher CO_2_ uptake compared to nonaqueous amines, they also suffer from high regeneration temperatures and energy penalties, increasing operational costs.^38–40^ Conventional water-lean solvents, on the other hand, offer lower energy regeneration but often at the expense of reduced CO_2_ capacity.^20,41,42^ It was therefore immediately notable to observe that MPZ in 2′HAP provided similar CO_2_ capacities in the absence of added water as either aqueous MEA or aqueous MPZ.^36^ We collected gas–liquid equilibrium data (presented here as absorption isotherms to highlight the pressure-dependent behavior) at concentrations ranging from 10 to 50 wt % MPZ, corresponding to 13.1–57.6 mol-percent (mol %) of MPZ in 2′HAP (Figure 2, Figures S2–S3, Table S1). These systems exhibited excellent uptake characteristics: for example, the 40 wt % MPZ/2′HAP solution absorbed 3.88 mmol CO_2_ per gram solution at 163 kPa, equivalent to 0.93 mol CO_2_ per mol MPZ and 4.09 mol CO_2_ per liter solution.

**Figure 2:**
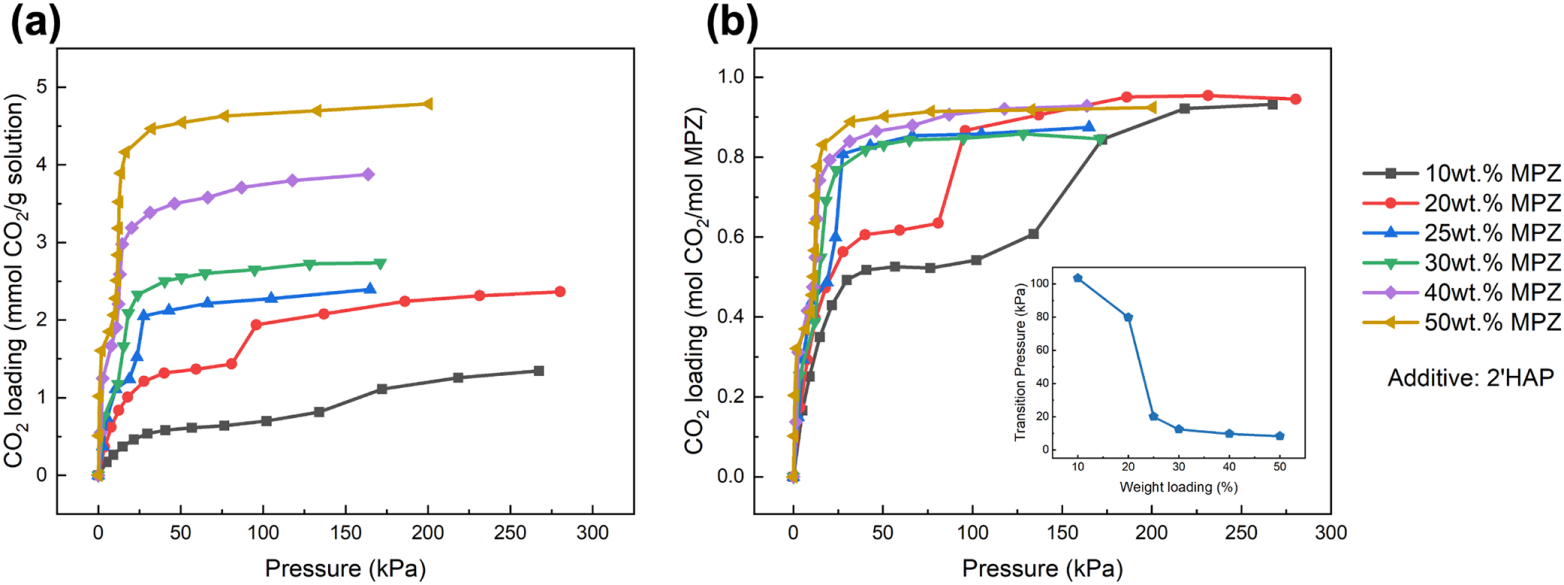
Pressure-dependent CO_2_ isotherms of 10 wt % (13.1 mol % MPZ), 20 wt % (25.4 mol % MPZ), 25 wt % (31.2 mol % MPZ), 30 wt % (36.8 mol % MPZ), 40 wt % (47.6 mol % MPZ), and 50 wt % (57.6 mol % MPZ) of MPZ in 2′HAP at 30 °C. Absorption data are reported in (a) gravimetric units (mmol CO_2_/g solution) and (b) amine efficiency (mol CO_2_/mol MPZ). Inset: transition pressure as a function of MPZ weight percentage. *P*_t_ defined as the first pressure at which the loading exceeds at least 20% above the initial plateau average, indicating the beginning of the transition.

This performance contrasts with the behavior of 1,4-dimethylpiperazine (DMPZ), a control compound with two tertiary amines. In water, DMPZ slowly reacts with CO_2_ via bicarbonate formation, highlighting the pathway’s feasibility but also its kinetic limitations.^43^ However, in 2′HAP, DMPZ exhibited negligible uptake, consistent with the absence of N–H groups required for carbamate formation and the suppression of bicarbonate formation in aprotic media (Figure S4). This dichotomy indicates that the high capacity of MPZ/2′HAP is not due to a conventional bicarbonate mechanism but instead involves a unique pathway where high uptake can be achieved even without bulk water.

Interestingly, all MPZ/2′HAP isotherms displayed reproducible (Figure S3) stepped isotherm behavior—a sharp, nonlinear increase in capacity at a threshold pressure—suggesting a change in absorption mechanism as a function of CO_2_ pressure and apparent cooperative behavior at higher pressures. The threshold step pressure (defined as described in Figure 2, Table S2) decreased with increasing MPZ concentration, with onsets from approximately 103 kPa for 10 wt % MPZ to approximately 8.3 kPa for the 50 wt % MPZ/2′HAP mixture. The cooperative CO_2_ absorption behavior of MPZ/2′HAP solutions was found to persist at different temperatures, moving to higher threshold pressures (and modestly lower peak capacities) at higher temperatures (Figure S5). This likely represents an advantage relative to a standard aqueous-based sorption system (for example, aqueous monoethanolamine) in a temperature-swing adsorption (TSA) process as the solvent can be cycled using lower temperature differentials between absorbing and desorbing similar to what has been shown in adsorption processes based on stepped-isotherms.^44^

The importance of the carbonyl group in the 2′HAP solvent was highlighted by changing the solvent to 2IPP or 2CP, inducing both a drop in maximum capacity (of approximately 28% and 52%, respectively) and the complete (2CP) or nearly complete (2IPP) disappearance of the stepped isotherm (Figure 3, Table S4). Indeed, the presence of only the acetyl group (MPZ/AP) provided good uptake (approximately 90% of the performance of MPZ/2′HAP) but also with a single-step isotherm. The other two hydroxyacetophenone isomers each provided relatively poor uptake (slightly less than 0.5 CO_2_ molecules per MPZ, about half the capacity with AP or 2′HAP), one (3′HAP) exhibiting a stepped isotherm while the other (4′HAP) did not (Figure 4, Figure S6, Table S5).

**Figure 3:**
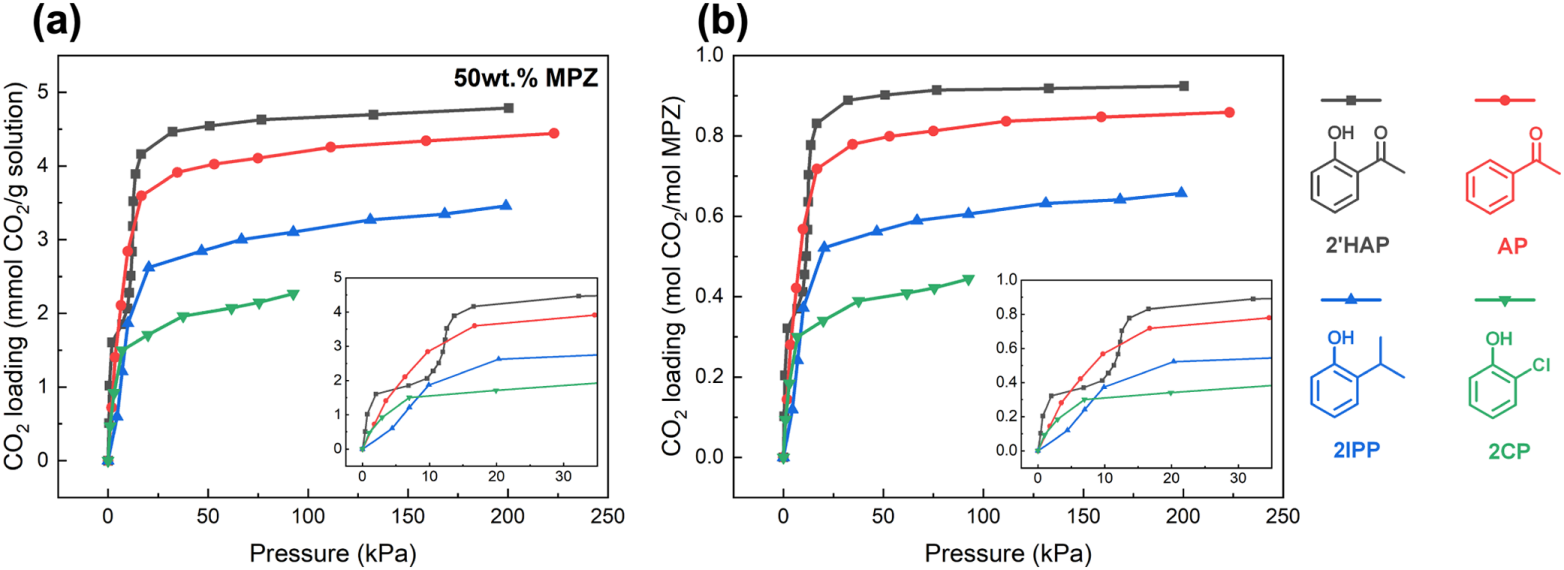
CO_2_ isotherms of 50 wt % MPZ in 2′HAP, AP, 2IPP, and 2CP at 30 °C. Absorption data are shown in (a) gravimetric units (mmol CO_2_/g solution) and (b) amine efficiency (mol CO_2_/mol MPZ). Insets show the isotherms in the low-pressure region.

**Figure 4:**
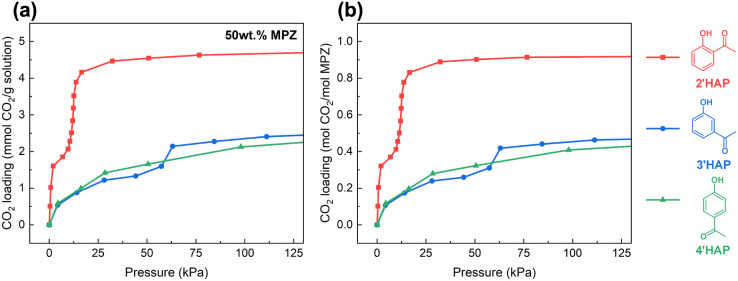
CO_2_ isotherms of 50 wt % of MPZ in 2′HAP, 3′HAP and 4′HAP solutions at 30 °C. Absorption data are shown in (a) gravimetric units (mmol CO_2_/g solution) and (b) amine efficiency (mol CO_2_/mol MPZ).

1-Methylpiperazine proved to be more effective than the more commonly used MEA and the structural analogue MP. MEA provided maximum uptake significantly less than 0.5 equiv of CO_2_ per amine, either alone or in solution with 2′HAP (Figure S7), while MP in 2′HAP reached a CO_2_-per-amine ratio of 0.47 (2.83 mmol CO_2_/g solution), close to the theoretical stoichiometric limit of 0.5 for carbamate formation. (We note that MEA made significant amounts of hydroxyl-stabilized imine with 2′HAP as indicated by NMR and IR spectra (Figures S8, S9), while no reaction of 2′HAP was observed with MP (Figures S10).) MPZ alone gave a single-step absorption isotherm with maximum uptake of 0.6 mol CO_2_ per mol MPZ (Figures S11). To correct for possible loss of amine during this experiment (approximately 30% MPZ was lost under vacuum at room temperature over 5–6 h), MPZ dissolved in the inert solvent IsoparM (a mixture of C_11_–C_16_alkanes) provided a capacity of 0.65 mol CO_2_ per MPZ in a single step (Figure 5, Figures S12, Table S6). In contrast, the use of 1,4-diisopropylbenzene (DIPB) or 2′-methoxyacetophenone (2′MAP) solvents with MPZ each gave much better uptake performance (CO_2_:MPZ stoichiometry ≈ 0.85 in both cases) and clear indications of cooperative behavior (i.e., a stepped isotherm).

**Figure 5:**
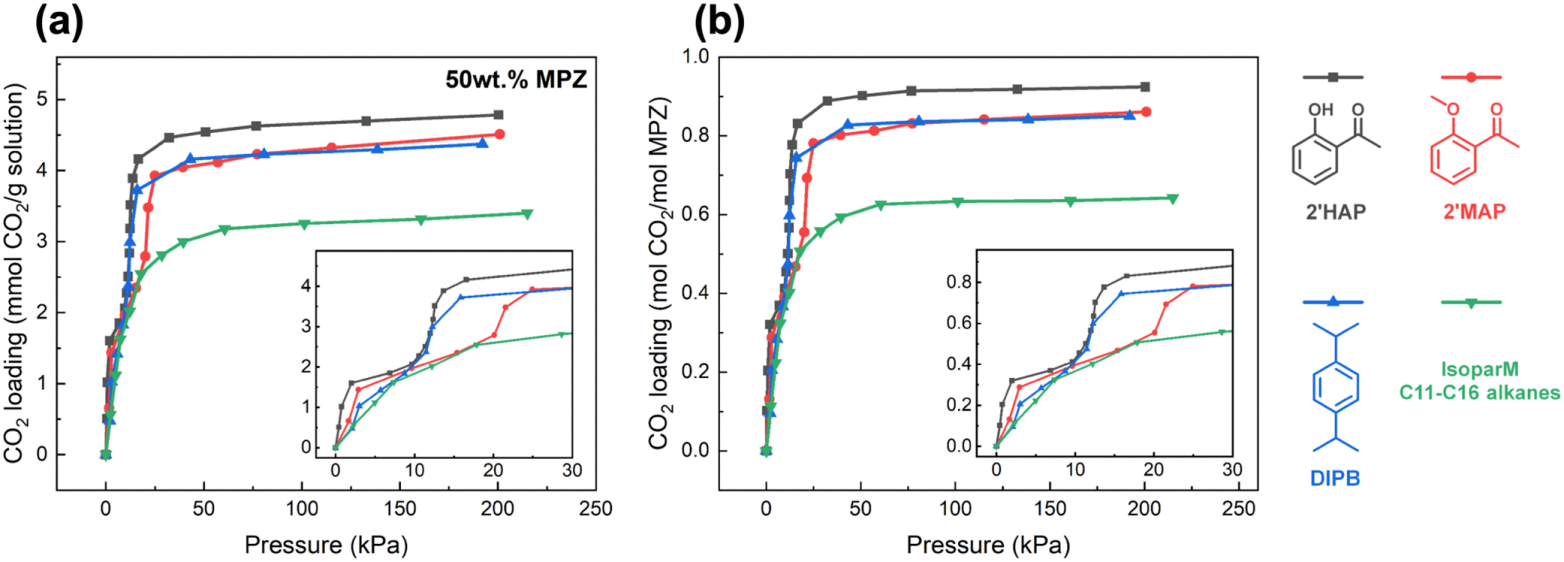
CO_2_ isotherms of 50 wt % of MPZ in 2′HAP, 2′MAP, DIPB and Isopar solutions at 30 °C. Absorption data are shown in (a) gravimetric units (mmol CO_2_/g solution) and (b) amine efficiency (mol CO_2_/mol MPZ).

It should be noted that physical parameters impose some constraints on the choices of bases and solvents, both of which must be liquid, nonvolatile, and miscible at standard operating temperatures (35–55 °C). Thus, piperazine and 2-methylpiperazine were considered but not used as these two solvents did not meet these criteria. Although 3′HAP and 4′HAP are solid at room temperature, they provided liquid solutions with MPZ at a 50/50 weight ratio.

### CO_2_ Capture from Dynamic Breakthrough Experiments

To corroborate the above isotherm results, we performed breakthrough experiments using approximately 10 g of MPZ/2′HAP solutions of varying composition, placed in a glass bubbler and exposed to three different CO_2_ concentrations spanning the range of stepped isotherm (Figure S13; see Methods for detailed information). The results closely mirrored the isotherm data (Figure 6): for example, the 40 wt % MPZ/2′HAP solution demonstrated an absorption capacity of 3.41 mmol CO_2_/g solution at 25% CO_2_, closely matching the isotherm uptake of approximately 3.25 mmol CO_2_/g solution. Furthermore, CO_2_ desorption was both rapid and complete at each CO_2_ concentration. Similarly good matches between isotherm and breakthrough data, as well as well-behaved desorption, were also observed for MPZ/AP solutions that do not exhibit stepped isotherm behavior (Figure S14).

**Figure 6:**
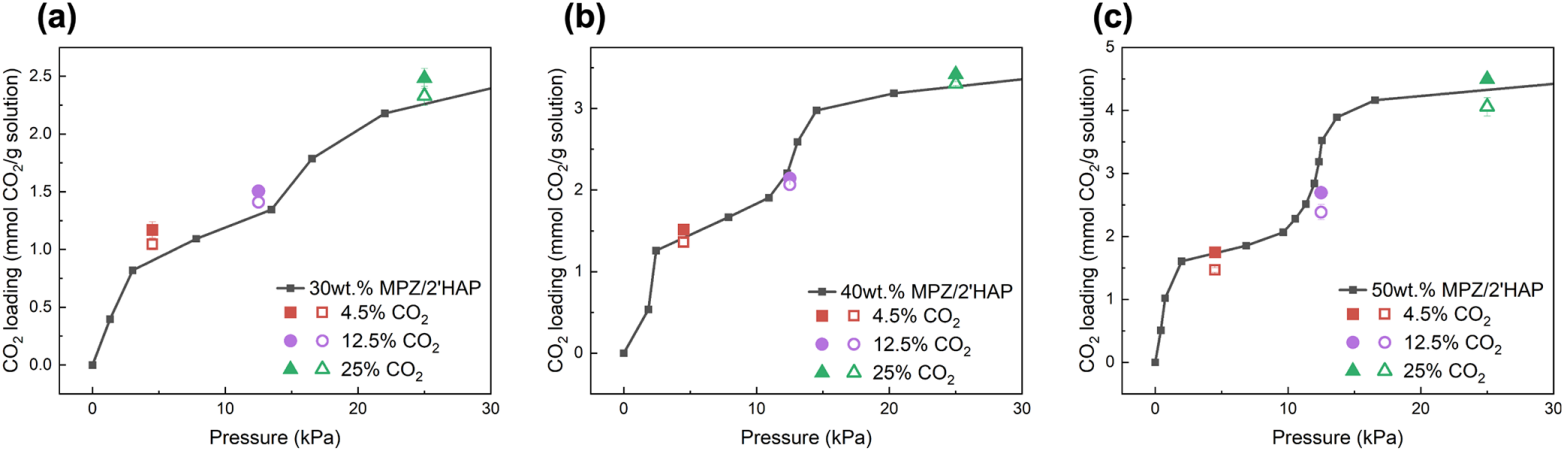
Comparison of (a) 30 wt %, (b) 40 wt %, and (c) 50 wt % of MPZ in 2′HAP breakthrough capacities (measured at 4.5%, 12.5% and 25% CO_2_ feed streams) with absorption isotherms (black points and trace), all at 30 °C. Filled symbols = absorption capacity; empty symbols = desorption capacity. Data are shown in gravimetric units (mmol CO_2_/g solution).

Beyond capacity validation, the breakthrough profiles provide additional insight into the absorption mechanism and phase behavior. At 25% CO_2_, MPZ/2′HAP exhibited a shock-dispersive-shock (S-D-S) wave pattern, in which sharp concentration fronts (“shock waves”) bracket a broadened transition region (“dispersive wave”) in the breakthrough curve. Such S-D-S behavior is commonly associated with stepped isotherms and collective uptake phenomena in solid-state adsorption systems, and arises when small changes in concentration lead to large changes in loading.^27,45,46^ At lower CO_2_ concentrations (4.5% and 12.5%), this feature is absent because the gas-phase partial pressure remains below or near the transition threshold of the stepped isotherm (Figures S15– S17). Furthermore, the S-D-S breakthrough pattern appears only in MPZ mixtures containing additives that promote cooperative stabilization of 1:1 MPZ:CO_2_ adducts (as discussed in the Methods section) such as 2′HAP, AP, 2′MAP, and DIPB (Figures S18– S22). In contrast, systems lacking this stabilization show single-wave profiles (Figure S23).

Phase behavior further modulates these signatures. Gelation or solid precipitation was observed in some systems but occurred at different stages depending on solvent polarity and product solubility. For MPZ/AP, gelation appeared well before saturation at 4.5% CO_2_, while MPZ/2′HAP remained fluid until higher CO_2_ concentrations where S-D-S patterns emerged. The impact of solubility is particularly evident in MPZ/DIPB: at 30 wt % MPZ, the solution exhibited clear S-D-S fronts and high CO_2_ capacity, whereas at 50 wt % MPZ, immediate gelation upon CO_2_ exposure suppressed the second shock and limited overall uptake (Table S11). These observations indicate that, while cooperative carbamic acid stabilization drives high uptake, solubility constraints and phase transitions can override this effect by introducing mass-transfer limitations. Importantly, the desorption profiles show that all absorbed CO_2_ can be fully released below 100 °C, even for systems that undergo gelation or solid formation and at 50 wt % MPZ, offering a significant regeneration advantage over conventional aqueous amines (>120 °C).^47^ This interplay between cooperative absorption and phase behavior underscores the complexity of MPZ-based water-lean solvents and their potential for energy-efficient CO_2_ capture.

### Proposed Mechanism

Table S1 summarizes the observations of uptake and isotherm pressure dependence described above. Figure 7 also contains this information, along with the proposed species and their interconversions relevant to this discussion. Mechanistic consideration of these results incorporates the following assumptions:

- The tertiary amine in MPZ serves as a base, not as a CO_2_-capturing nucleophile.
- The initial carbamic acid adducts of secondary amines require stabilization to effectively sequester CO_2_.
- Protonation at either N atom of MPZ renders the molecule unable to bind CO_2_. If protonated at the tertiary amine, the secondary amine of MPZ is rendered inactive toward CO_2_ since nucleophilicity and basicity are likely to be related, and protonation of the second amine of MPZ is approximately 30,000 times more difficult than the first.^48^
- Phenolate anions are not nucleophilic enough to bind CO_2_, even with a carbonyl nearby to stabilize the adduct.

**Figure 7:**
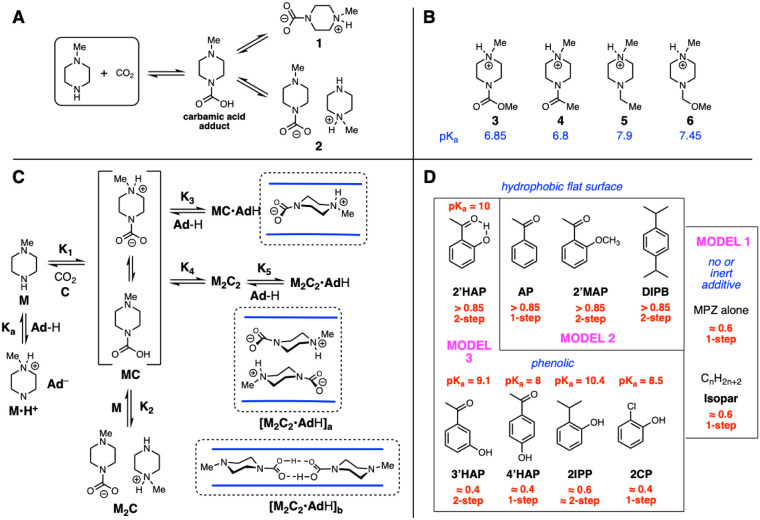
(A) Fundamental types of species formed by MPZ and CO_2_. (B) p*K*_a_ values of the conjugate acids of piperazine derivatives calculated using Advanced Chemistry Development (ACD/Laboratories) software in SciFinder. (C) Proposed equilibria involved in the MPZ-CO_2_ system containing additives (AdH) in which H may or may not be an acidic proton. Blue lines indicate hydrophobic surfaces that can be stabilized by flat hydrophobic additive molecules. (D) Additives (solvents) used in this study with their p*K*_a_ values, maximum CO_2_:MPZ molar ratios, and pressure-dependent isotherm patterns shown in red. The “models” refer to the calculated interplay of different equilibria described below and in Supporting Information.

We therefore propose that the carbamic acid adduct can undergo proton transfer intramolecularly (the second amine center of MPZ abstracting the proton to form zwitterion **1** (Figure 7A), giving a maximum molar ratio CO_2_:MPZ of 1.0) or intermolecularly (a second equivalent of amine giving salt **2**, providing a maximum molar ratio CO_2_:MPZ of 0.5). Therefore, given its maximum molar ratio of ≈0.6 (Figure S11), MPZ alone apparently provides mostly **2** with a minor contribution from **1**. We suggest that the relative instability of **1** reflects deactivation of the tertiary amine by carbonation of the transannular amine. This is supported by calculations of the basicities of model compounds **3**-**6**, showing that secondary amine carbonation or acylation suppresses tertiary amine basicity by 3–10 fold (Figure 7B).

Thus, the role of solvents that boost uptake (AP, 2′HAP, 2′MAP, DIPB) must be to stabilize the carbamic acid, **1**, or to create higher-order aggregates with a 1:1 CO_2_:base ratio. The appearance of 2-step absorption isotherms usually, but not always, correlated with uptake approaching the ideal 1:1 stoichiometry. NMR analysis of materials formed upon the exposure of MPZ/additive mixtures to natural-abundance or ^13^C-enriched CO_2_ is provided in Section S6 of the Supporting Information, along with a discussion of these results. These spectra provide two key pieces of information: the presence of at least two MPZ•CO_2_ species, and their dependence on concentration and the exclusion of other H-bonding molecules. However, no direct insights into detailed structures were obtained from these data due to the dynamic nature of these materials.

Our results are consistent with the following hypotheses. (1) By protonation of MPZ, the most acidic phenolic additives (2CP, 4′HAP, and 3′HAP) diminish the CO_2_ sorption capacity of their mixtures. (2) Stabilization of monocarbamate **(MC)** or dicarbamate **(M**_**2**_ **C**_**2**_) occurs by virtue of favorable hydrophobic interactions with additives that have extended flat structures (2′HAP, 2′MAP, AP, DIPB). This is reminiscent of the hydrophobic component to recognition of glucosyl and galactosyl residues by carbohydrate-binding proteins.^49–51^ (3) Therefore, a 1:1 MPZ:CO_2_ stoichiometry is favored by the formation of adduct structures that are both flat and as nonpolar as possible. The tertiary amine component of MPZ aids in this regard by providing charge balance without steric hindrance. (4) Polarity or H-bond accepting ability is deleterious to such hydrophobic stabilization, supported by two lines of evidence: (i) DMSO-*d*_6_ solvent eliminates the NMR signature of a second MPZ-CO_2_ structure; and (ii) 2′HAP and 2IPP share similar p*K*_a_ values but differ somewhat in their ability to support high-capacity CO_2_ absorption by MPZ. We suggest that this is due to internal H-bonding available in 2′HAP, which makes the OH group less active in external H-bonding than 2IPP. (5) A head-to-tail dimeric structure such as **[M**_**2**_ **C**_**2**_ **•Ad**H]_**a**_ is consistent with the following observations. (i) The additional ^13^C NMR resonances for the MPZ ring appear only at higher concentrations, suggesting that these are due to dimers (or higher-order aggregates, which are not considered here). (ii) Morpholine (MP), which is only 0.6 p*K*_a_ units less basic than MPZ^52^ and could easily adopt the head-to-head dimeric structure of **[M**_**2**_ **C**_**2**_ **•Ad**H]_**b**_, is ineffective (maximum 0.47 equiv of CO_2_ in a single-step isotherm, Figure S7).

### Network Modeling

It is apparent from the experimental results that the nature of the compound in which MPZ is dissolved (the “additive”) makes a considerable difference to the performance of the base in these systems. If the additives were used as solvents in large molar excess relative to MPZ, the relatively simple equilibria of Figure 7A could be used in all cases, accounting for solvent effects by assigning different values of solvent-dependent equilibrium constants. However, the molar amounts of additives and MPZ were not very different in many mixtures tested, and so it should be more accurate to model the effects of additives by positing discrete (and concentration-dependent) interactions between additive and MPZ-CO_2_ species. Furthermore, the hydroxyacetophenones explored here have acidities that are in the range of MPZ basicities, and therefore acid–base equilibria are likely to be important.

Taking into account the possible interactions shown in Figure 7C, three different scenarios of interacting equilibria (Figure 8) are proposed to cover different types of additives: (1) solvent-free or the nonaromatic low-polarity solvent IsoparM, (2) nonphenolic aromatic additives, and (3) phenolic additives. In the first case, only three equilibria were included since the branched hydrocarbon IsoparM is expected to be inert, whereas the possibilities of noncovalent additive interactions and MPZ protonation by phenolic additives added more potential equilibria to Models 2 and 3. These models do not aim to provide precise values for individual equilibrium constants (which cannot be unambiguously determined from the isotherm data alone) but rather to explore how shifts in key equilibria (e.g., carbamate stabilization, dimerization, or amine protonation) could reproduce the range of isotherm shapes and capacities observed experimentally. By varying the relative strengths of these interactions in a physically reasonable range, we can test whether the proposed framework is consistent with the empirical trends across different additive families. It is important to emphasize that these models assume that all steps are at equilibrium and do not account for kinetic differences or nucleation phenomena.

**Figure 8:**
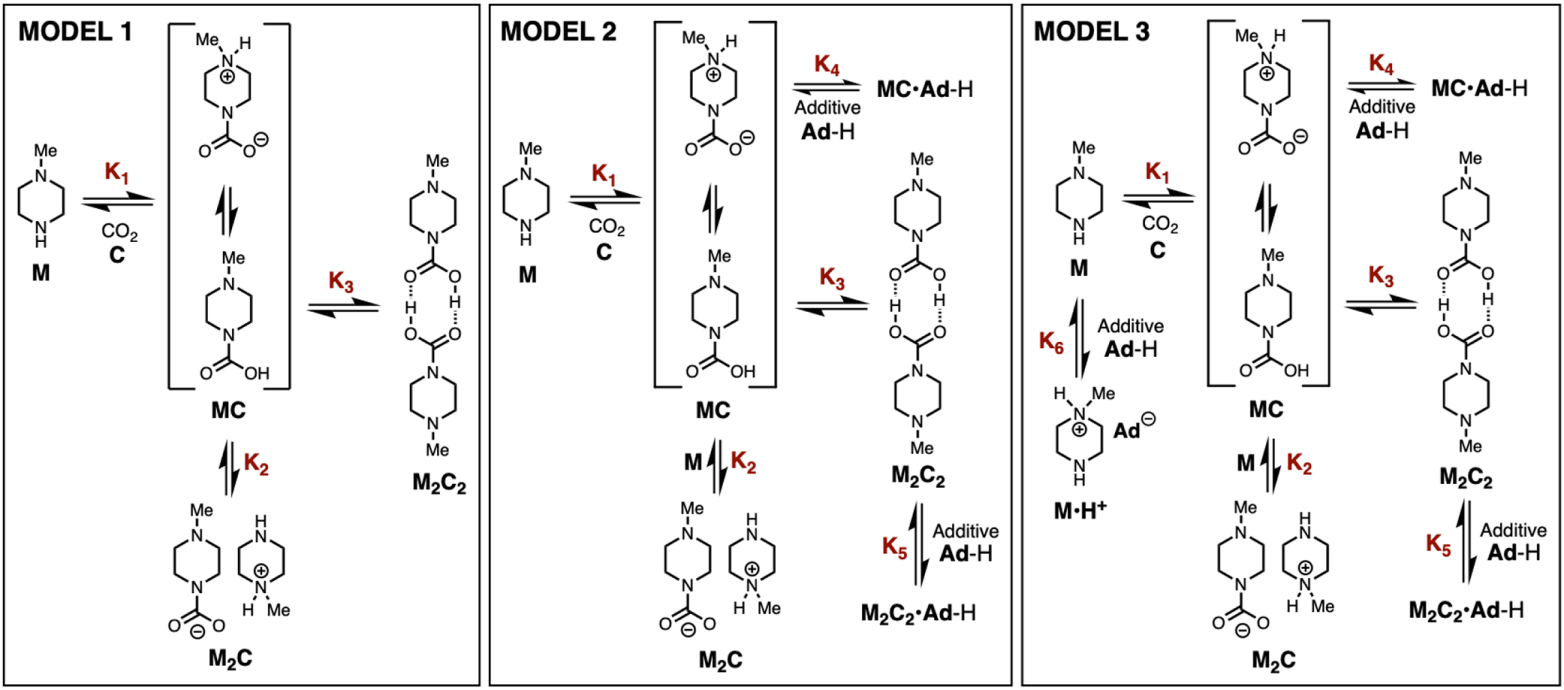
CO_2_ binding equilibria for MPZ under the following conditions: Model 1 = no or inert additive; Model 2 = nonphenolic aromatic additives; Model 3 = phenolic additives.

The interaction of CO_2_ with MPZ in the absence of additives was treated with Model 1, but ignoring K_3_ because of the observed uptake (0.6 equiv. per MPZ) and the expectation that dimerization in this manner is likely to be minimal without an additive. The best approximation to the single-step isotherm behavior with maximum capacity of 0.6 equiv per base was obtained with K_1_ ≈ 50–100 M^–1^ (Figure S38). [These values represent apparent equilibrium constants based on molar concentrations; activity coefficients were assumed to be unity for the purpose of this initial modeling.] Using this K_1_ value as the approximate fundamental association constant of CO_2_ and MPZ, equilibrium CO_2_ adsorption as a function of pressure was then calculated over ranges of the other equilibrium constants using Model 2 (for nonphenolic aromatic additives) and Model 3 (incorporating protonation of MPZ as an additional step for phenolic additives).

For Model 2, a key experimental observation was the transition from a single-step isotherm for AP to two-step isotherms for 2′MAP and DIPB. This behavior could not be produced in predicted CO_2_ pressure-dependent curves using only K_1_ and K_2_, even when adding values for K_6_ (protonation of MPZ by an acidic additive) (Figure S39). The addition of K_3_ alone or K_4_ alone gave hints of two-step patterns, but only in rare combinations (Figure S39). More distinct two-step predictions were produced only when all of the equilibria in Models 2 or 3 were brought into play, in particular when K_2_ and K_4_ were changed in opposite directions from a set of equilibrium constant values that give a single-step high-capacity isotherm, as shown in Figure S40. In other words, an intermediate step is introduced when MPZ (K_2_) and additive (K_4_) compete for association with the carbamate or zwitterionic adduct (**MC**). We suggest that 2′MAP and DIPB act in opposite ways, as one is more polar and the other less polar than AP. Thus, the nonpolar DIPB induces greater association of MPZ with the initial CO_2_ complex, raising K_2_ and inducing a more gradual step profile. In contrast, we suggest that 2′MAP associates with the **MC** complex better than AP, raising K_4_ and giving a more defined step. In general, single-step isotherms are predicted to shift to lower CO_2_ concentrations as the dimerization event (K_3_) is enhanced, as well as the subsequent association with additive (K_5_) (Figure S41).

In the presence of phenolic additives, a key experimental observation is the change from the efficient two-step isotherm with 2′HAP to inefficient 2-step (3′HAP) and 1-step (4′HAP) patterns with the other phenolic ketones. Model 3 reveals two ways this could occur (Figure S42): remove active MPZ by protonation (increase K_6_) or (as above for Model 2) enhance formation of the bimolecular ammonium carbamate **M**_**2**_ **C** (increase K_2_) while disfavoring the association of additive with the unimolecular carbamate **MC** (decrease K_4_). Both factors are likely in play, as 3′HAP and 4′HAP are both more acidic than 2′HAP, and 2′HAP may form uniquely effective interactions with **MC**. The only additive with the same diminished acidity as 2′HAP (2IPP) performs less well (maximum CO_2_:MPZ stoichiometry of 0.6), presumably because its values of K_4_ or K_5_ are lower than for 2′HAP. A variety of factors affect the two-step isotherm behavior produced in the interplay of equilibria in Model 3, some of which are illustrated in Figure S43. We note in that figure that two-step isotherms can occur with the plateau at different degrees of base occupancy, depending largely on the relative values of K_2_ (formation of the **M**_**2**_ **C** complex), K_3_ (formation of the **M**_**2**_ **C**_**2**_ dimer), and K_4_ (stabilization of **MC** by association with additive).

## Conclusions

We describe here the CO_2_ capture performance of nonaqueous mixtures of 1-methylpiperazine with acetophenone and related additives, energized by the observation that the MPZ/2′HAP system exhibits cooperative (stepped) isotherms, enhanced CO_2_ uptake at lower pressures (surpassing the 0.5 amine efficiency threshold), and maintains cooperative absorption even at higher temperatures, which is advantageous for temperature swing absorption (TSA) processes. Breakthrough experiments further validated the system’s reliability, showing consistent CO_2_ absorption and desorption capacities across varying CO_2_ concentrations. Related acetophenone analogs (AP, 3′HAP, 4′HAP) and structurally varied solvents (2′MAP, DIPB, 2IPP, 2CP) were tested for comparison, alongside MEA and MP as a control amine. These studies and accompanying modeling of proposed interlocking equilibria suggest that additive acidity, polarity, and ability to engage in hydrophobic stabilization of various amine-CO_2_ molecular species are important in CO_2_ uptake and isotherm shape. These results demonstrate the scientific viability and mechanistic promise of nonaqueous, stepped-isotherm solvents for CO_2_ capture, motivating further process-level evaluation to assess their engineering and economic performance. The study underscores the importance of solvent-amine interactions and molecular structure in optimizing carbon capture technologies, paving the way for future exploration of next generation water-lean solvents.

While this work focuses on the initial observation and subsequent mechanistic understanding of the cooperative absorption between CO_2_, MPZ, and 2′HAP, more work is needed to translate these findings to practical application. For new solvents to leave the laboratory and be used in the field, engineering-relevant parameters such as foaming potential, oxidative stability, interaction with metals, and the system’s tolerance to water and humidity will be needed. Furthermore, while nonideal intermolecular interactions can potentially suppress amine volatility in these formulated mixtures, future process designs will need to incorporate standard engineering controls, such as water or solvent-wash sections, to strictly manage any residual solvent loss during long-term cycling. We have also not yet considered the potential use of the CO_2_-induced MPZ-2′HAP gelation phenomenon observed here. It may be possible to take advantage of this process to downsize CO_2_ capture equipment (e.g., by filtering out the gels and regenerating those separately from the supernatant solvent).

## Supplementary Material













## Notes

Experimental details, including (i) all isotherms and breakthrough uptake measurements and profiles, (ii) density measurements, (iii) NMR data as a function of composition, concentration, and exposure to ^12^CO_2_ and ^13^CO_2_, (iv) network modeling showing changes in speciation and uptake profiles with defined changes in equilibrium constants, and (v) five movies showing modeling predictions at various combinations of equilibrium constants for Models 1–3, for restricted values of K_1_ (PDF)
Calculated distribution of species in the Model 1 scheme (MOV)
Distributions adding nonzero values for K_3_ (MOV)
Distributions adding nonzero values for K_4_ (MOV)
Distribution of species in the Model 2 scheme (MOV)
Distribution of species in the Model 3 scheme (MOV)
