# Supplementary material for: Liquid-Phase CO 2 Capture by a Nonaqueous Cooperative Absorption Mechanism
Source: J Am Chem Soc. 2026 Jun 4;148(23):24430–40. doi: 10.1021/jacs.6c06590 (PMC13281383; doi:10.1021/jacs.6c06590)
Supplement: Supplementary file 1 [file ja6c06590_si_001.pdf]

Supplementary Information

Liquid-phase CO_2 capture by a non-aqueous cooperative absorption mechanism

Lu Lu[1], Ankana Roy[2†], Saptarshi Chatterjee[3†], Stephen Schuyten[4], M. G. Finn[2]*, Ryan P. Lively[1]*

[1] School of Chemical & Biomolecular Engineering, Georgia Institute of Technology, Atlanta, Georgia 30332, United States

[2] School of Chemistry and Biochemistry, Georgia Institute of Technology, Atlanta, Georgia 30332, United States

[3] Department of Biological Sciences, Virginia Tech, Blacksburg, Virginia 24061, United States

[4] Johnson Matthey Process Technologies Inc., Savannah, Georgia 31408, United States

* corresponding authors: mgfinn@gatech.edu, ryan.lively@chbe.gatech.edu

† these authors contributed equally

Table of Contents

SI.1. Summary of uptake results ... 3
SI.2. CO_2 isotherms of MPZ in different solutions ... 4
SI.2.1. MPZ in aqueous solutions compared with literature data ... 4
SI.2.2. Density and alternate equilibrium representations of MPZ/2'HAP ... 5
SI.2.3. Repeated measurements of CO_2 isotherms of MPZ/2'HAP solutions ... 6
SI.2.4. CO_2 isotherms of DMPZ/2'HAP solution ... 6
SI.2.5. CO_2 isotherms of 40 wt.% MPZ in 2'HAP at different temperatures ... 7
SI.2.6. CO_2 isotherms of MPZ in AP, 3'HAP and 4'HAP solutions ... 7
SI.2.7. CO_2 isotherms of MEA, 40 wt.% MEA/2'HAP and 50wt.% MP/2'HAP ... 8
SI.3. Characterization of control amine (MEA and MP) with 2'HAP ... 9
SI.3.1 ^{1}H NMR and ATR-IR of MEA ... 9
SI.3.2 ^{13}C NMR of MP and MP/2'HAP ... 10
SI.4. Additional CO_2 isotherms of pure MPZ, MPZ in 2'MAP, DIPB and Isopar ... 10
SI.5. CO_2 Breakthrough results of MPZ in different solvents ... 12
SI.5.1 Schematic of liquid breakthrough system ... 12
SI.5.2. Breakthrough uptake of MPZ/AP solutions ... 12
SI.5.3. Breakthrough profiles ... 13
SI.6. NMR analysis ... 22
SI.7. Steady-State Equilibrium Analysis of MPZ–CO_2 Reaction Networks ... 30
SI.7.1 MPZ in Low-Polarity or Solvent-Free Conditions (Model 1) ... 30
SI.7.2 MPZ in non-innocent, non-protic additives (Model 2) ... 32
SI.7.3 MPZ in phenolic additives (Model 3) ... 33
SI.7.4 Numerical implementation for MPZ equilibria ... 35
SI.7.5 Representations of Modeling Results ... 35
SI.8. Isotherm raw data tables ... 41
References ... 49

SI.1. Summary of uptake results.

Table S1 summarizes the results described in the main text and figures, reproduced here (along with the molecular structures) for easier reference.

Table S1. Summary of key parameters of CO_2 uptake. Red text in each composition highlights the variable changed from the reference composition (highlighted in green).

Composition	max capacity (mmol/g)	CO_2/amine molar ratio	isotherm trace	notes
10wt.% MPZ/2'HAP	1.34	≈ 0.93	2-step	P_t decreases with increasing MPZ concentration
20wt.% MPZ/2'HAP	2.37	≈ 0.94	2-step	
25wt.% MPZ/2'HAP	2.4	≈ 0.87	2-step	
30wt.% MPZ/2'HAP	2.74	≈ 0.85	2-step	
40wt.% MPZ/2'HAP	3.88	≈ 0.93	2-step	
50wt.% MPZ/2'HAP	4.79	≈ 0.92	2-step	
pure MPZ	5.8	0.58	1-step	
50wt.% MPZ/2IPP	3.46	0.66	slightly 2-step	
50wt.% MPZ/2CP	2.27	0.44	1-step	
50wt.% MPZ/AP	4.44	0.86	1-step	capacity and step don't always correlate
50wt.% MPZ/3'HAP	2.58	0.48	2-step	
50wt.% MPZ/4'HAP	2.76	0.50	1-step	
50wt.% MPZ/2'MAP	4.51	0.86	2-step	
50wt.% MPZ/DIPB	4.38	0.85	2-step	
50wt.% MPZ/Isopar	3.4	0.64	1-step	
Pure MEA	6.02	0.36	1-step	a dip in the isotherm trace could indicate weak 2-step behavior
40wt.% MEA/2'HAP	1.61	0.23	slightly 2-step	
50wt.% MP/2'HAP	2.84	0.47	1-step	

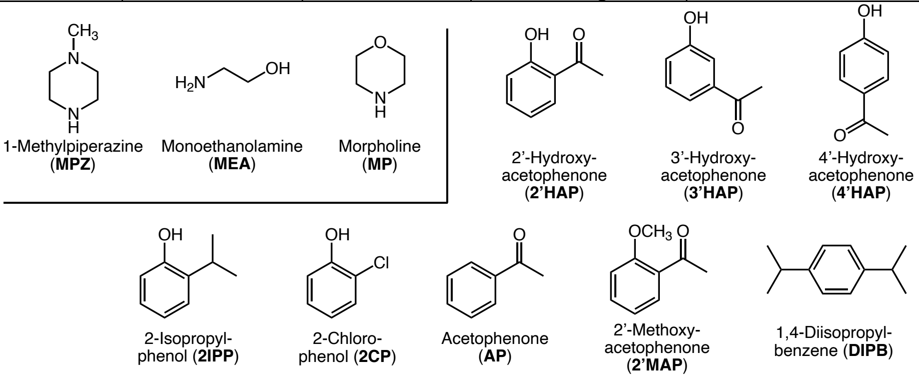

Table S2. Summary of the transition pressure values (Values are approximate averages based on discrete isotherm points; precision is limited by measurement resolution).

composition	average transition pressure (kPa)	run 1	run 2	run 3	run 4
10wt.%MPZ/2'HAP	103.40 ± 1.31	102.07	104.68	103.46	—
20wt.%MPZ/2'HAP	80.17 ± 1.70	80.88	80.76	77.87	—
25wt.%MPZ/2'HAP	20.02 ± 1.08	18.79	20.48	20.8	—
30wt.%MPZ/2'HAP	12.39 ± 0.94	11.8	11.9	13.47	—
40wt.%MPZ/2'HAP	9.70 ± 1.24	10.61	8.54	10.93	8.73
50wt.%MPZ/2'HAP	8.26 ± 1.23	6.85	9.11	8.82	—

SI.2. CO_2 isotherms of MPZ in different solutions

SI.2.1. MPZ in aqueous solutions compared with literature data

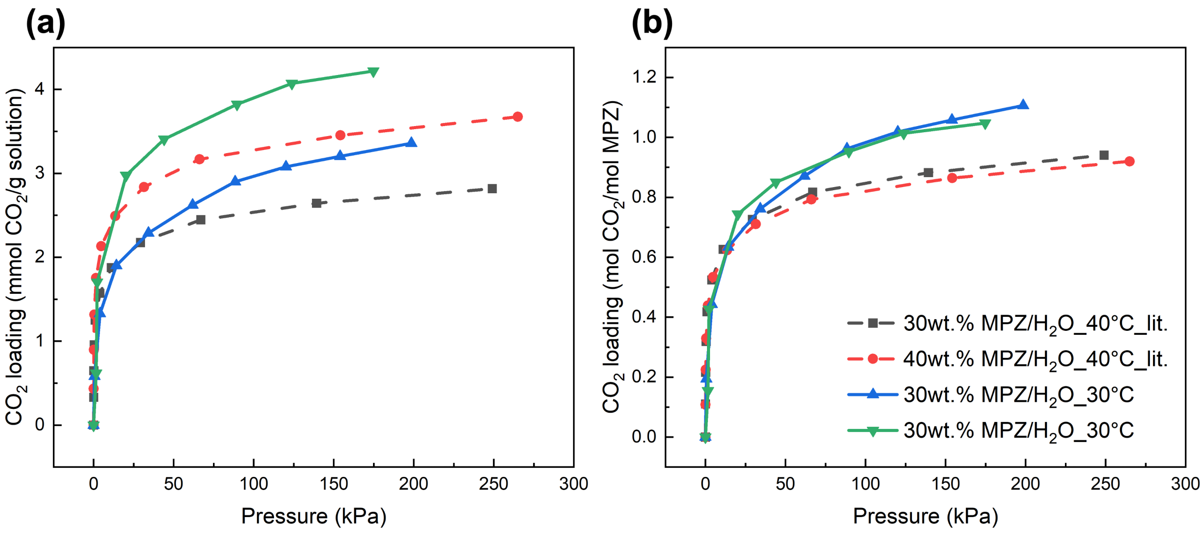

Figure S1. CO_2 isotherms of 30 wt.% and 40 wt.% of aqueous MPZ at 30 °C (measured) and 40 °C (literature[2]). Absorption data are reported in (a) gravimetric units (mmol CO_2/g solution) and (b) amine efficiency (mol CO_2/mol MPZ).

The literature data were obtained at 40 °C, reflecting the typical operating temperature of amine scrubbing towers in industrial applications. To reduce solvent evaporation during the vacuum process of our measurements, all isotherms were measured at 30 °C; our results exhibited a similar trend to previously reported data with higher uptake at the lower temperature, as expected.

SI.2.2. Densities and alternate equilibrium representations for MPZ/2'HAP

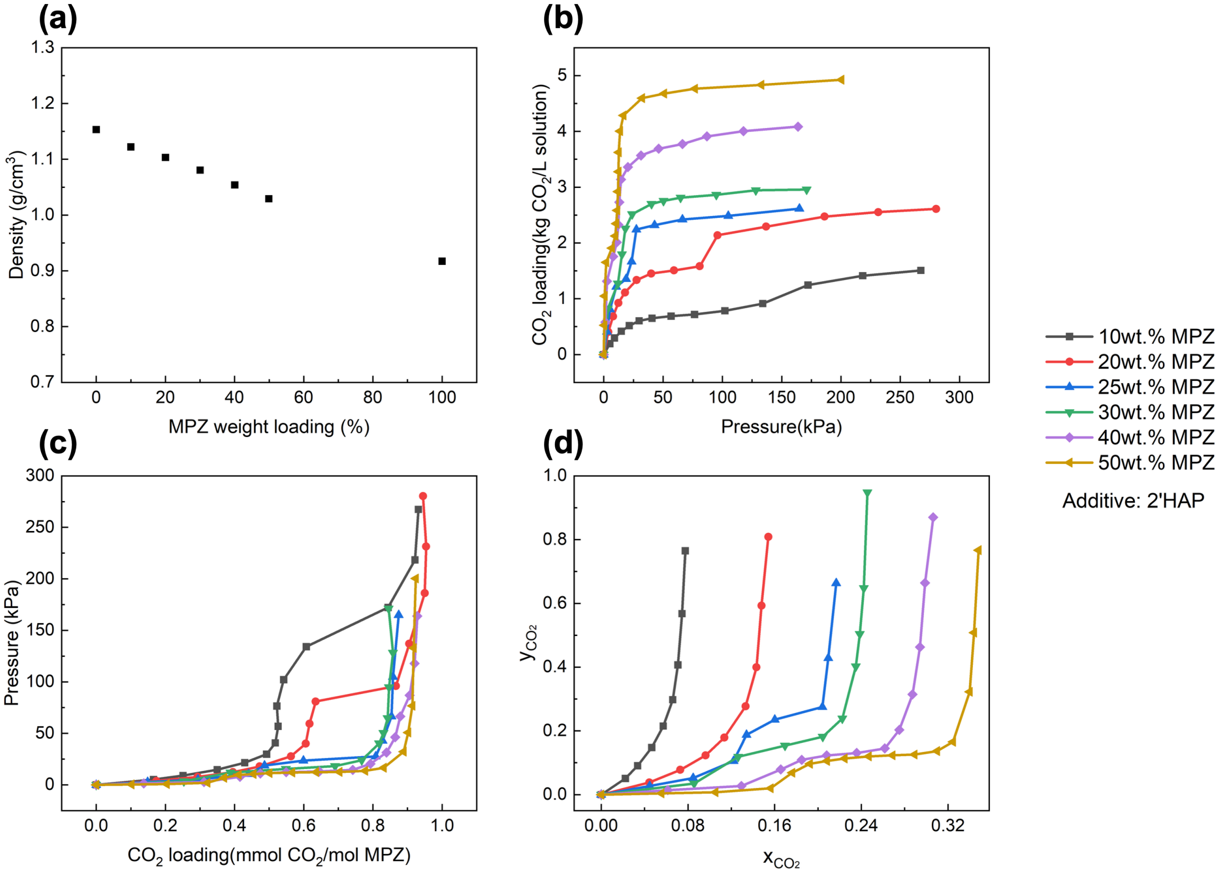

Figure S2. Physical properties and alternate equilibrium representations for MPZ/2'HAP solutions at 30 °C. (a) Density; (b) CO_2 isotherms (reported in mol CO_2/L solution); (c) VLE represented as equilibrium CO_2 pressure versus amine efficiency (mol CO_2/mol MPZ). (d) Standard VLE diagram displaying the gas-phase CO_2 mole fraction (y_{CO_2}) as a function of the liquid-phase CO_2 mole fraction (x_{CO_2}), calculated assuming a constant total system pressure of 1 bar.

SI.2.3. Repeated measurements of CO2 isotherms of MPZ/2'HAP solutions

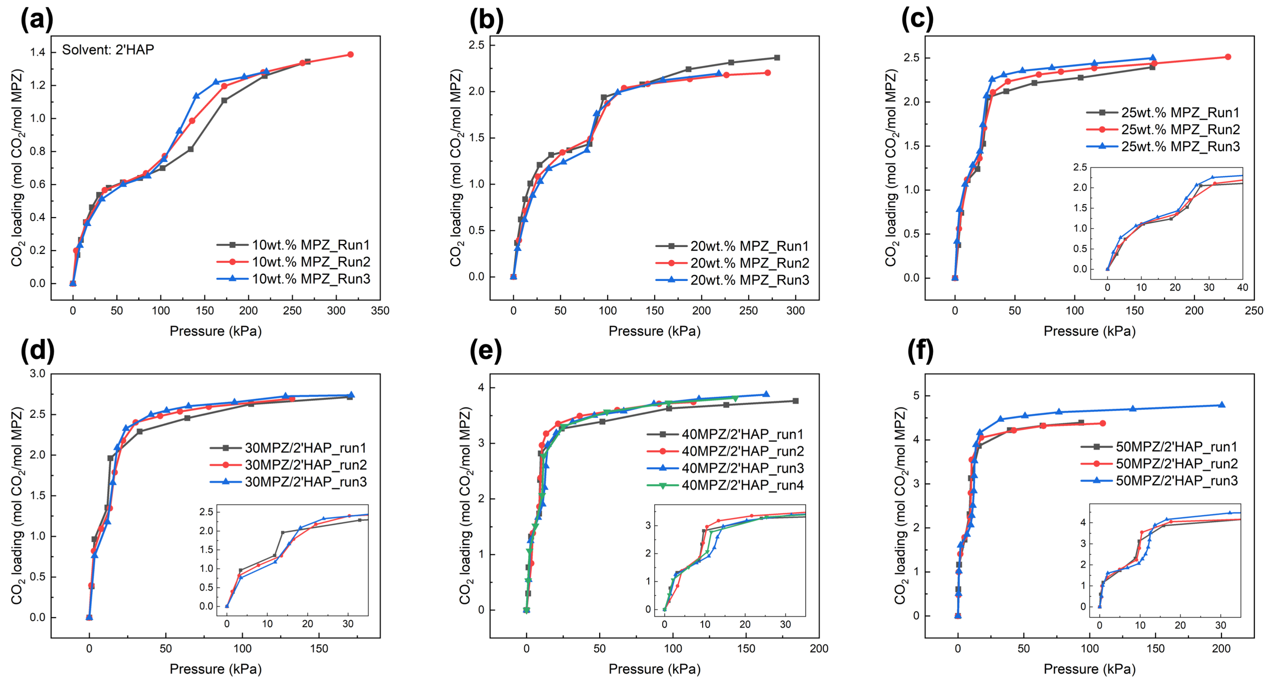

Figure S3. CO_2 isotherms of (a) 10 wt.% MPZ/2'HAP; (b) 20 wt.% MPZ/2'HAP; (c) 25 wt.% MPZ/2'HAP; (d) 30 wt.% MPZ/2'HAP; (e) 40 wt.% MPZ/2'HAP; (f) 50 wt.% MPZ/2'HAP at 30 °C. Absorption data are reported in gravimetric units (mmol CO_2/g solution). Insert: the isotherms at low-pressures regions.

SI.2.4. CO_2 isotherms of DMPZ/2'HAP solution

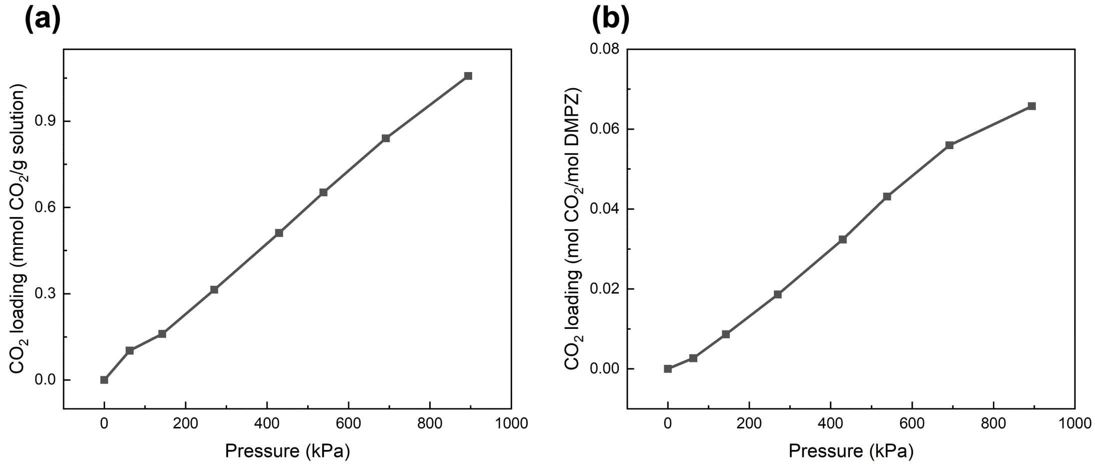

Figure S4. CO_2 isotherms of 50 wt.% DMPZ/2'HAP. Absorption data are reported in (a) gravimetric units (mmol CO_2/g solution) and (b) amine efficiency (mol CO_2/mol DMPZ).

SI.2.5. CO_2 isotherms of 40 wt.% MPZ in 2’HAP at different temperatures

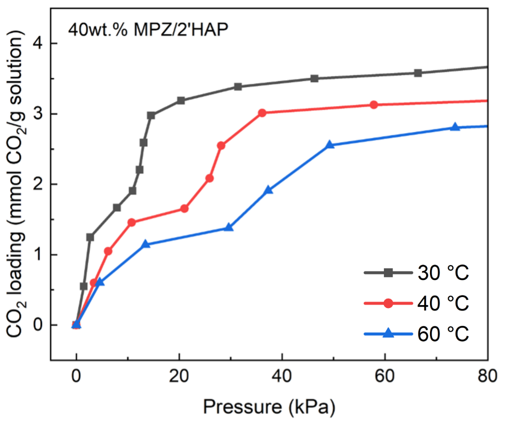

Figure S5. CO_2 isotherm of 40 wt.% MPZ/2’HAP at 30, 40 and 60 °C

SI.2.6. CO_2 isotherms of MPZ in AP, 3’HAP and 4’HAP solutions

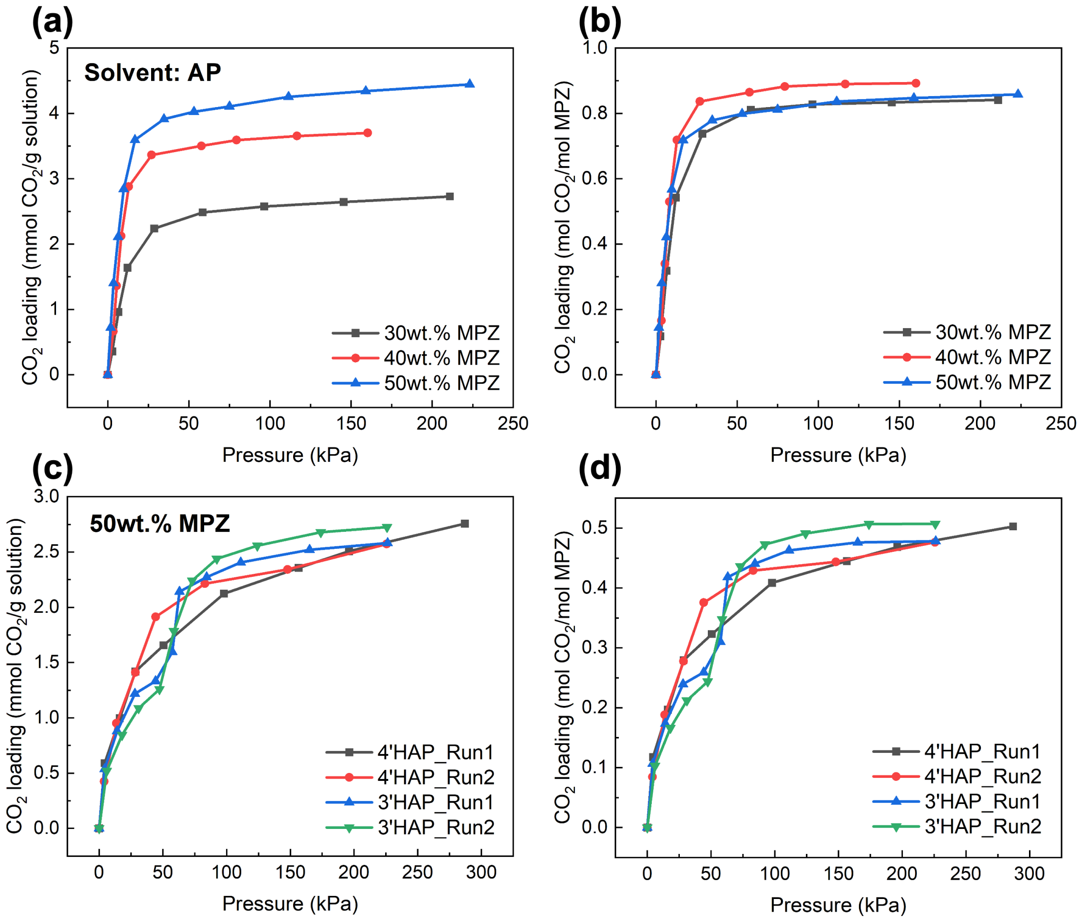

Figure S6. CO_2 isotherms of (a/b) 30, 40 and 50 wt.% of MPZ in AP; (c/d) 50 wt.% of MPZ in 3'HAP and 4'HAP solutions at 30℃. Absorption data are reported (a/c) in gravimetric units, i.e. mmol CO_2/g solution; (b/d) in amine efficiency, i.e. mol CO_2/mol MPZ.

SI.2.7. CO_2 isotherms of MEA, 40 wt.% MEA/2'HAP and 50wt.% MP/2'HAP

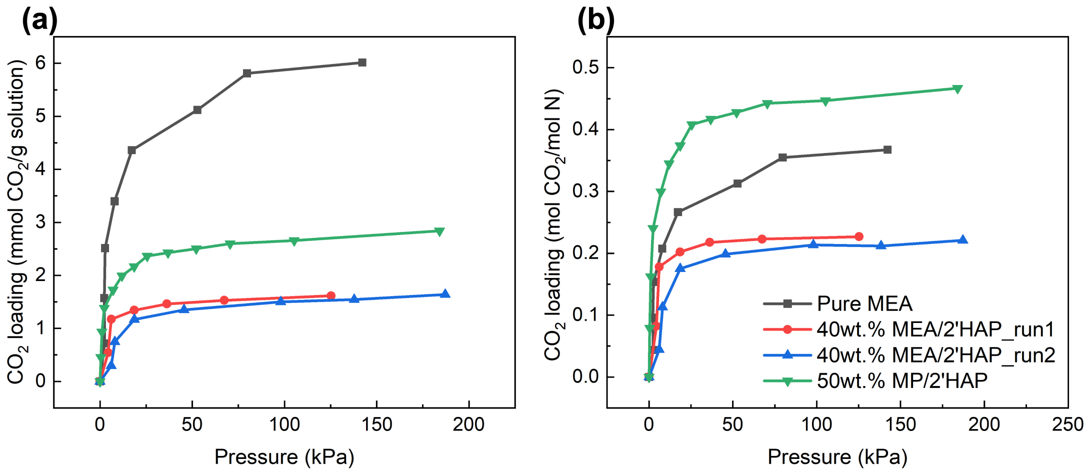

Figure S7. CO_2 isotherms of MEA, 40 wt.% MEA/2'HAP and 50MP/2'HAP solutions at 30℃. Absorption data are reported in (a) gravimetric units (mmol CO_2/g solution) and (b) amine efficiency (mol CO_2/mol MPZ).

SI.3. Characterization of control amine (MEA and MP) with 2'HAP

SI.3.1 ¹H NMR and ATR-IR of MEA

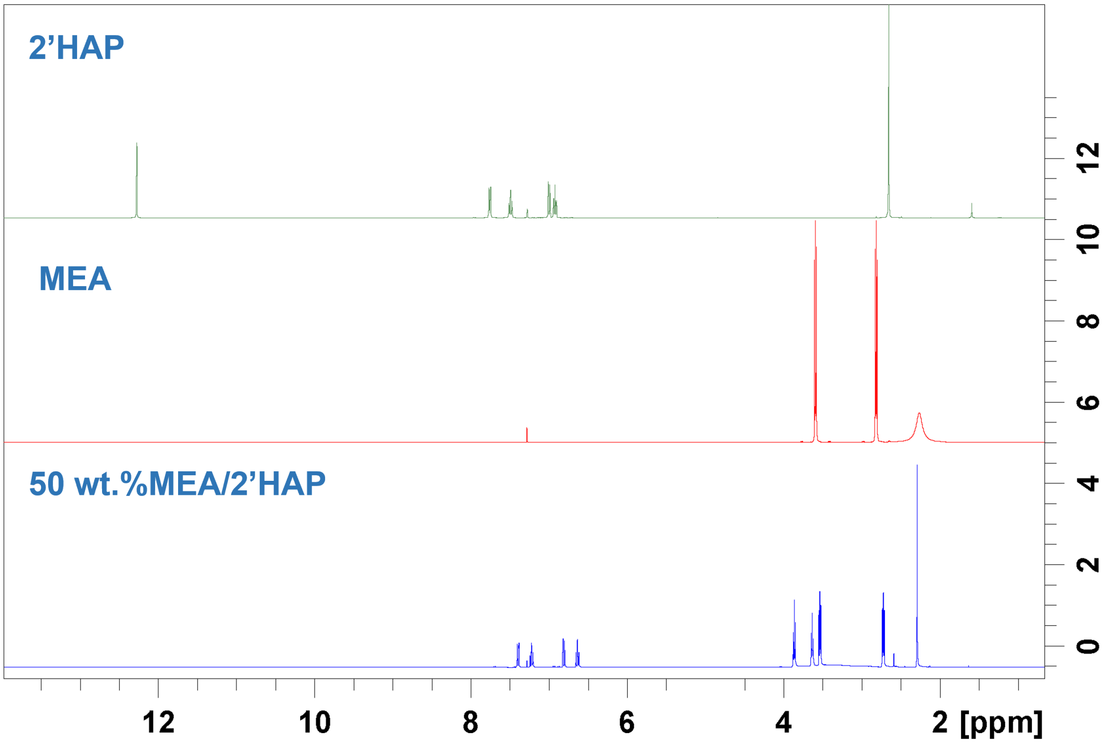

Figure S8. ^{1}H NMR of 2'HAP, MEA and 50 wt.% MEA/2'HAP in $CDCl_3$.

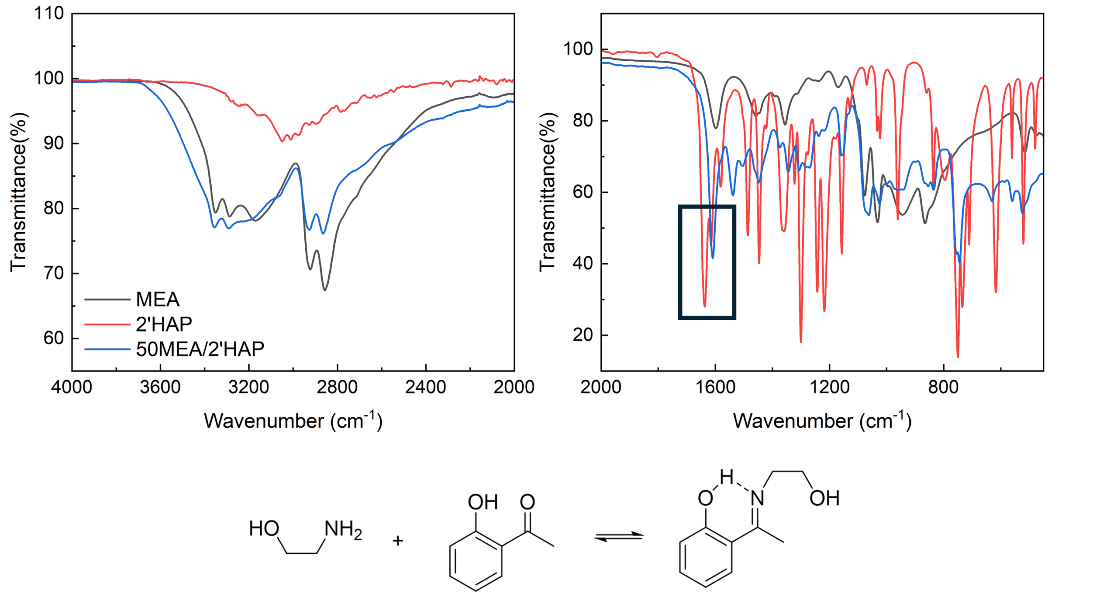

Figure S9. ATR-IR of 2'HAP, MEA and 50 wt.% MEA/2'HAP and the reaction path of MEA with 2'HAP.

SI.3.2 ^{13}C NMR of MP and MP/2'HAP

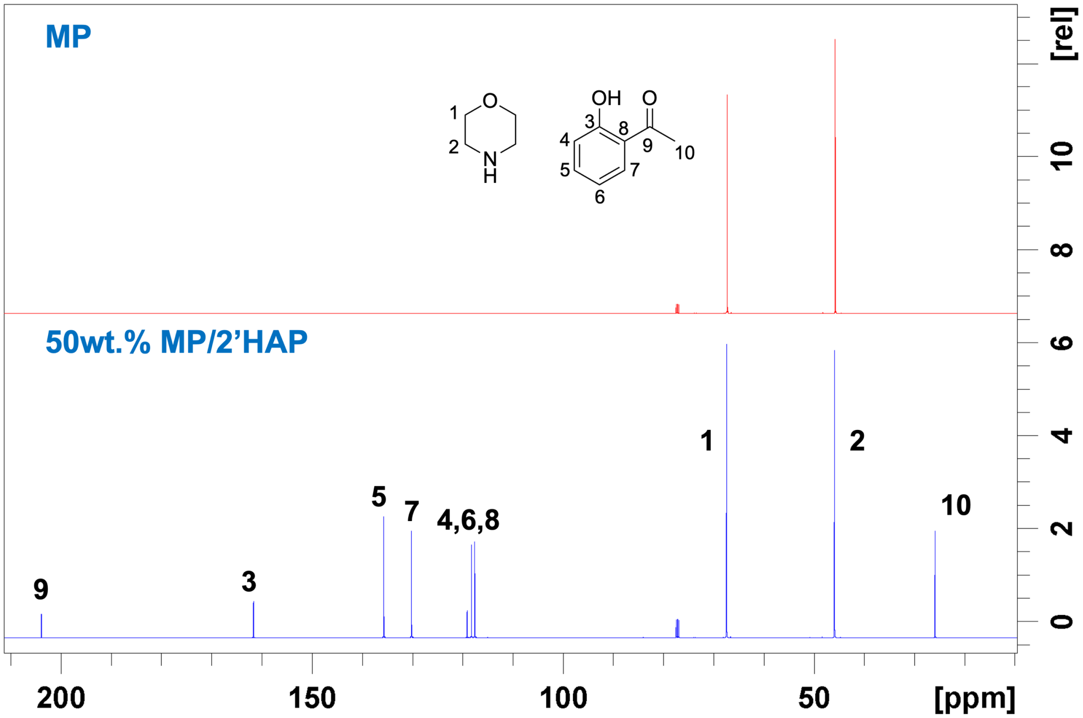

Figure S10. ^{13}C NMR of MP and 50 wt.% MP/2'HAP in $CDCl_3$.

SI.4. Additional CO_2 isotherms of pure MPZ, MPZ in 2'MAP, DIPB and Isopar

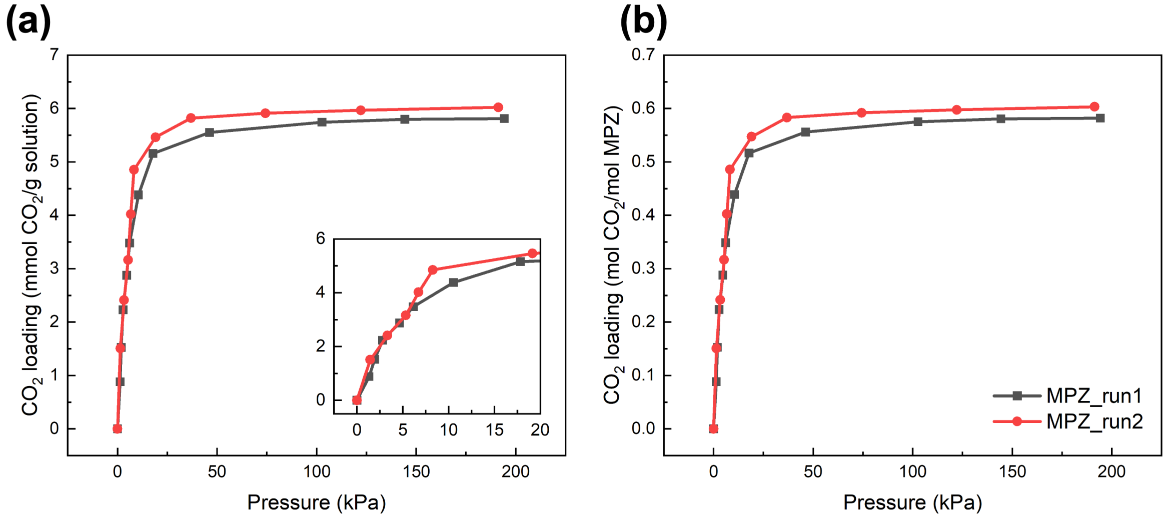

Figure S11. CO_2 isotherms pure MPZ at 30℃. Absorption data are reported in (a) gravimetric units (mmol CO_2/g solution) and (b) amine efficiency (mol CO_2/mol MPZ).

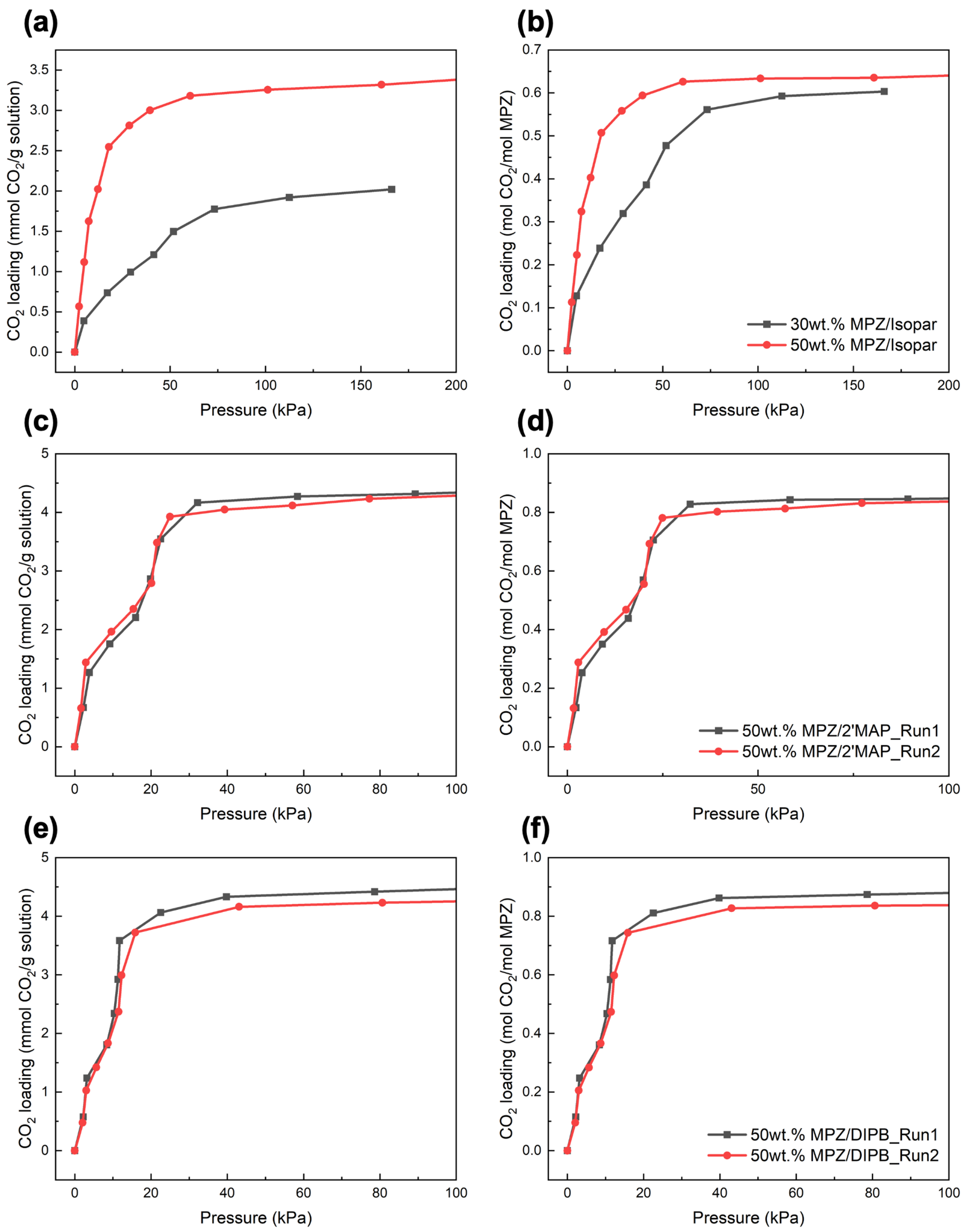

Figure S12. CO_2 isotherms of (a/b) 30 and 50 wt.% of MPZ in Isopar; (c/d) 50 wt.% of MPZ in 2'MAP; (e/f) 50 wt.% of MPZ in DIPB at 30℃. Absorption data are reported in (a/c/e) gravimetric units (mmol CO_2/g solution) and (b/d/f) amine efficiency (mol CO_2/mol MPZ).

SI.5. CO_2 Breakthrough results of MPZ in different solvents

SI.5.1 Schematic of liquid breakthrough system

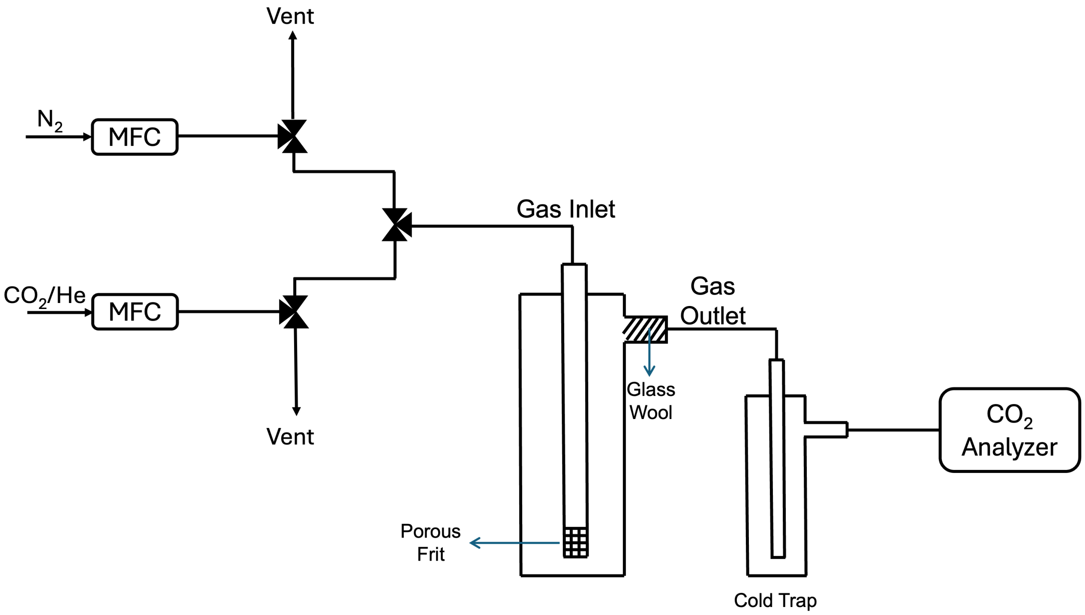

Figure S13. Schematic of liquid breakthrough system.

SI.5.2. Breakthrough uptake of MPZ/AP solutions

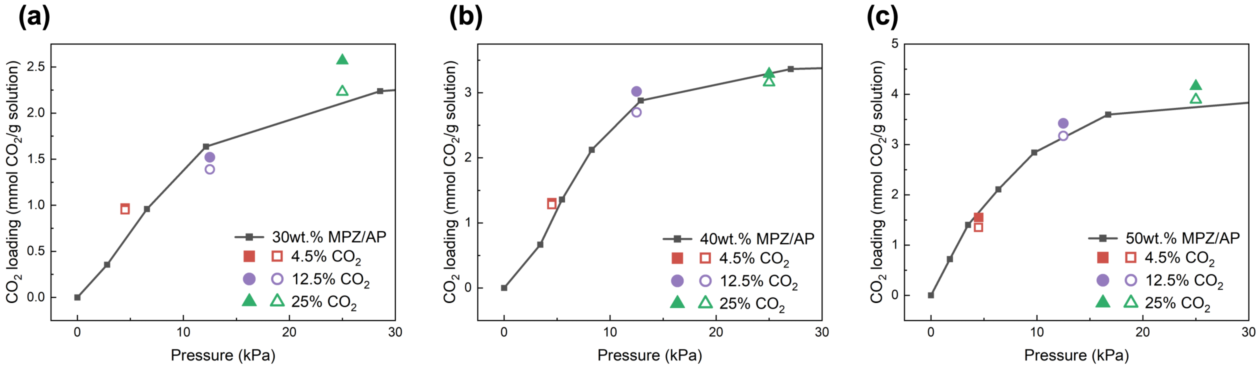

Figure S14. Comparison of (a) 30 wt.%, (b) 40 wt.%, and (c) 50 wt.% of MPZ in AP breakthrough capacity with isotherms at 30℃ with 4.5%, 12.5% and 25% CO_2. Filled symbols = absorption capacity; empty symbols = desorption capacity. Data are shown in gravimetric units (mmol CO_2/g solution).

SI.5.3. Breakthrough profiles

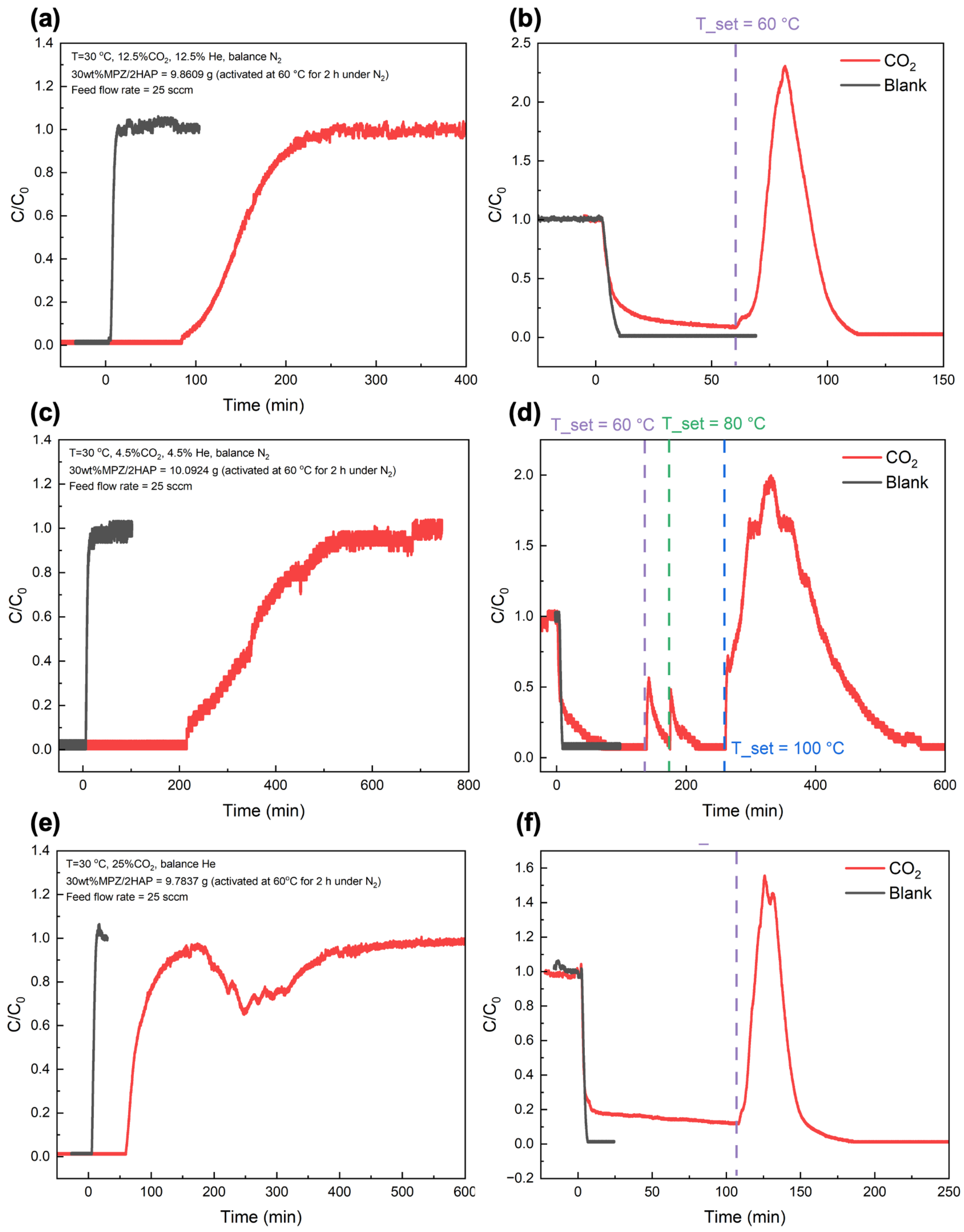

Figure S15. Breakthrough profile of 30 wt.% MPZ/2'HAP with CO2 concentrations of 12.5%, 4.5% and 25%. (a/c/e) absorption; (b/d/f) desorption. Temperature = 30℃, Pressure = 1 atm.

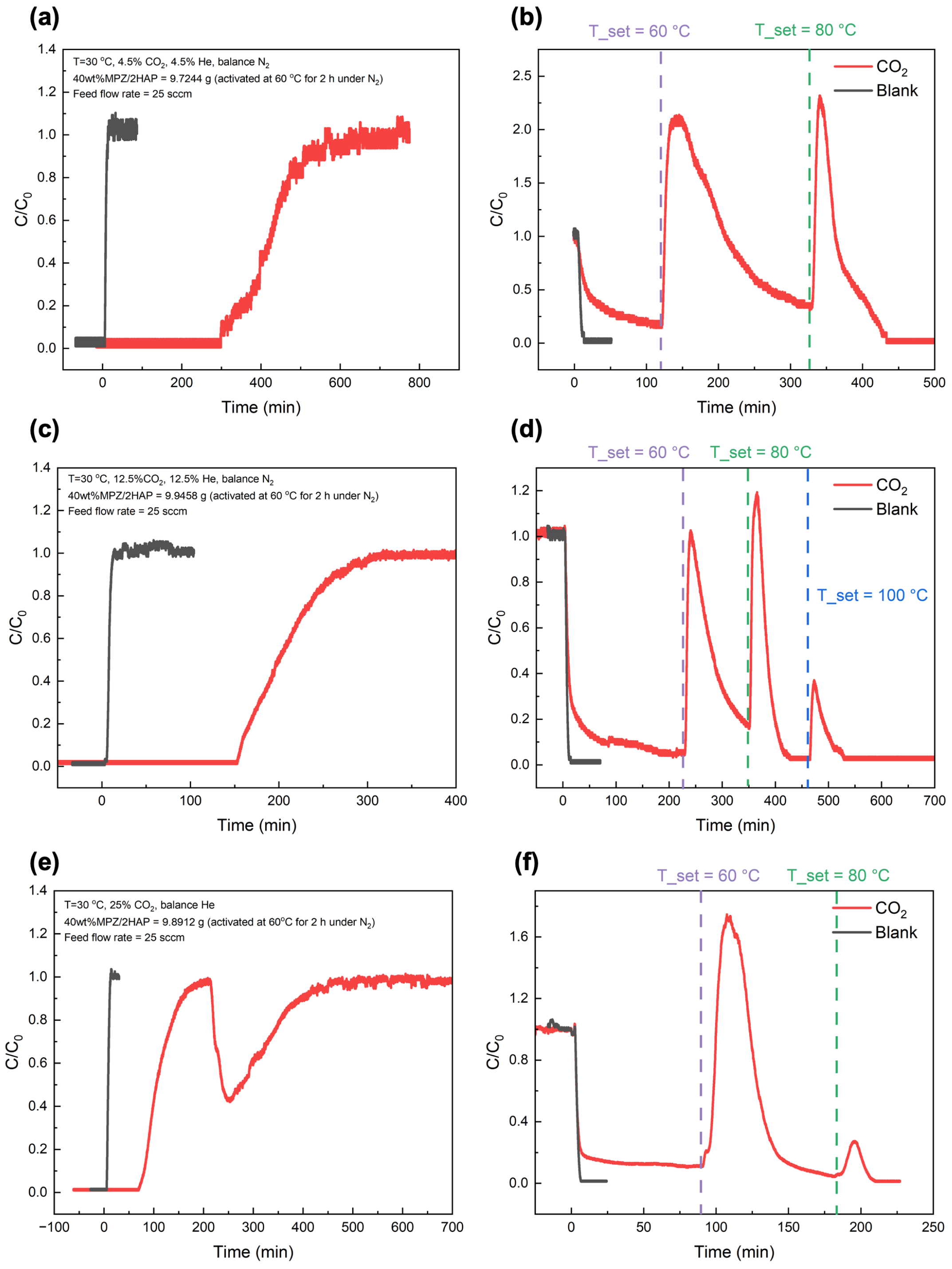

Figure S16. Breakthrough profile of 40 wt.% MPZ/2'HAP with CO_2 concentrations of 4.5%, 12.5% and 25%. (a/c/e) absorption; (b/d/f) desorption. Temperature = 30℃, Pressure = 1 atm.

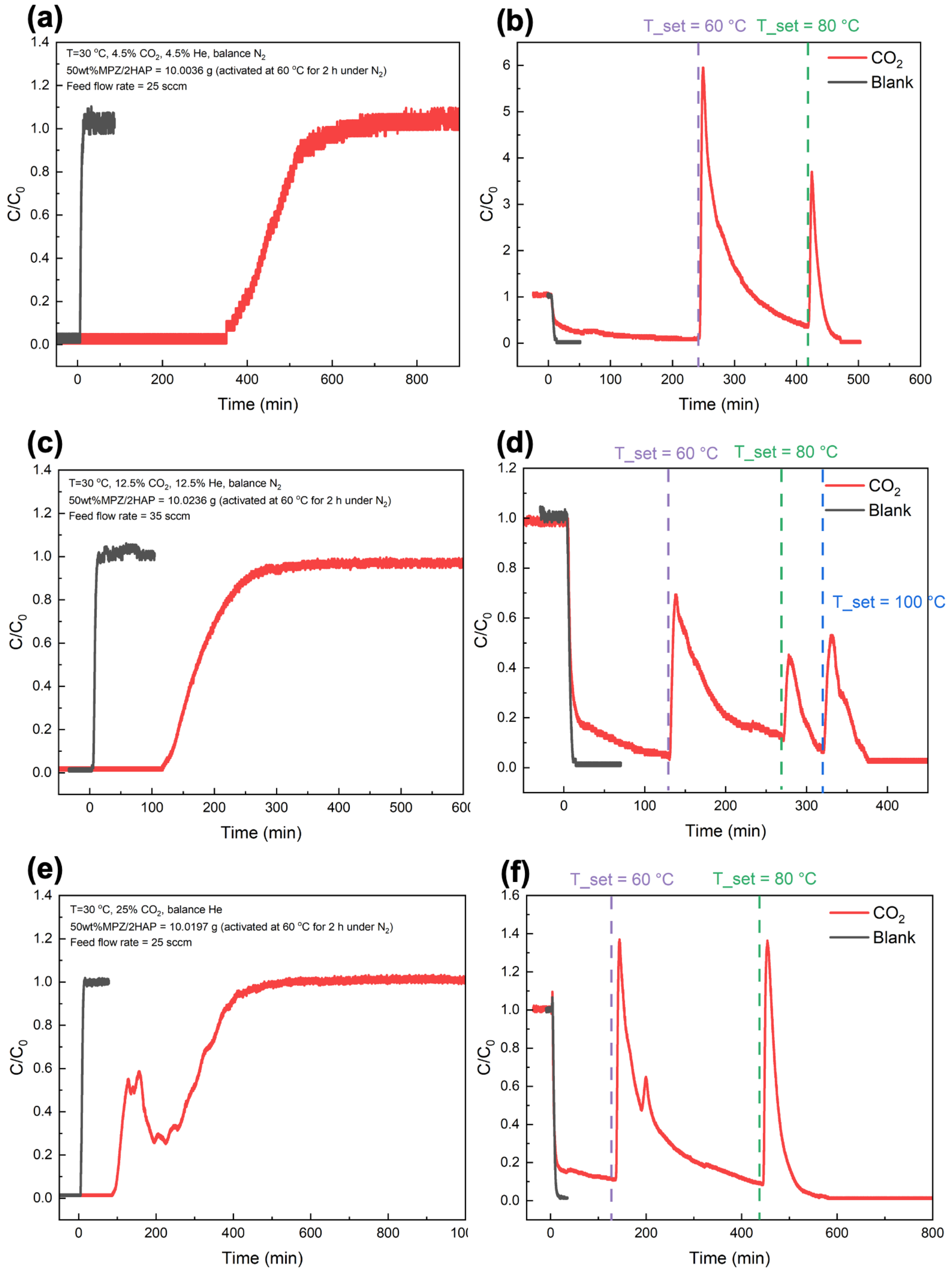

Figure S17. Breakthrough profile of 50 wt.% MPZ/2'HAP with CO_2 concentrations of 4.5%, 12.5% and 25%. (a/c/e) absorption; (b/d/f) desorption. Temperature = 30 30℃, Pressure = 1 atm.

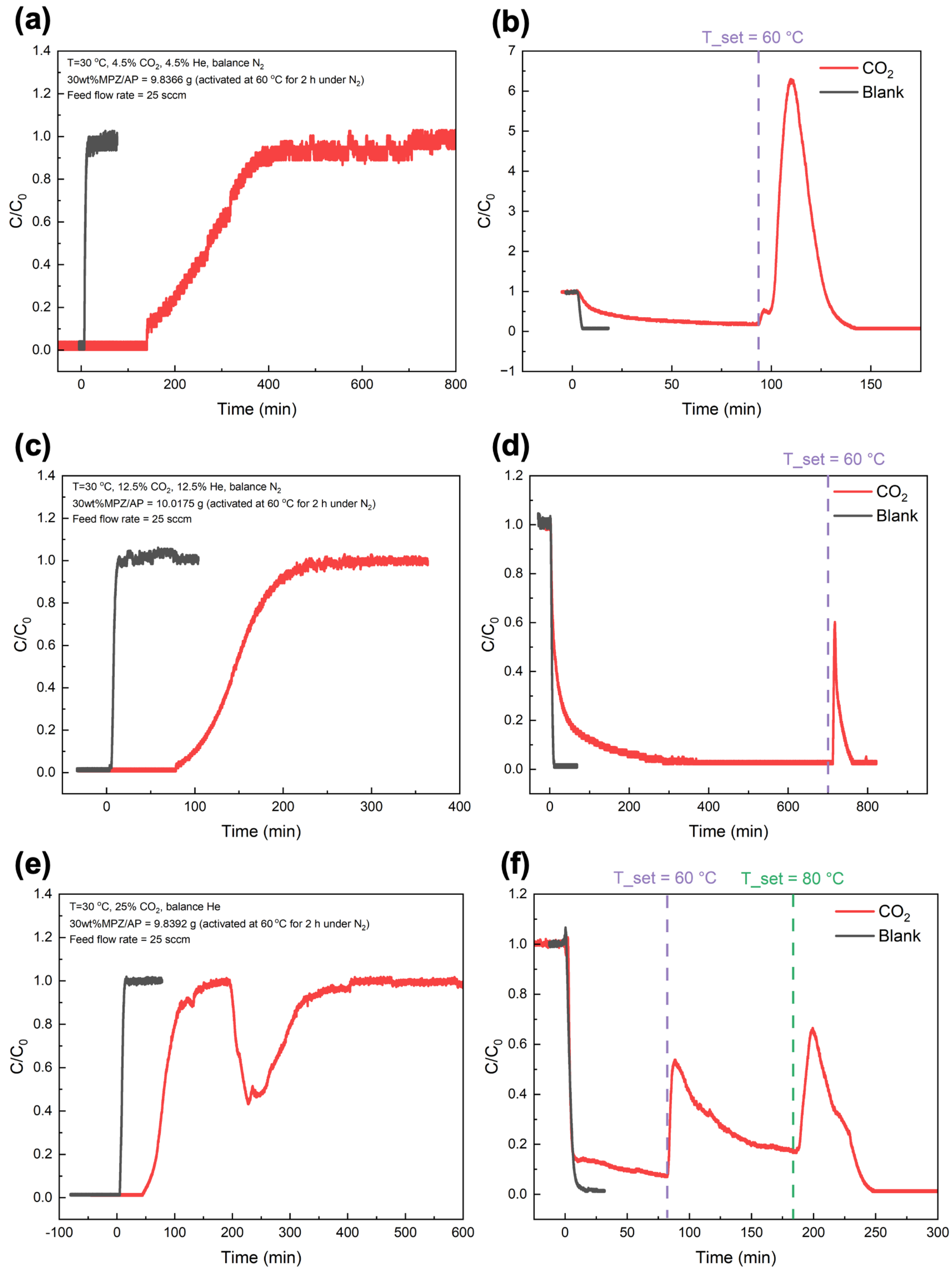

Figure S18. Breakthrough profile of 30 wt.% MPZ/AP with CO_2 concentrations of 4.5%, 12.5% and 25%. (a/c/e) absorption; (b/d/f) desorption. Temperature = 30 30℃, Pressure = 1 atm.

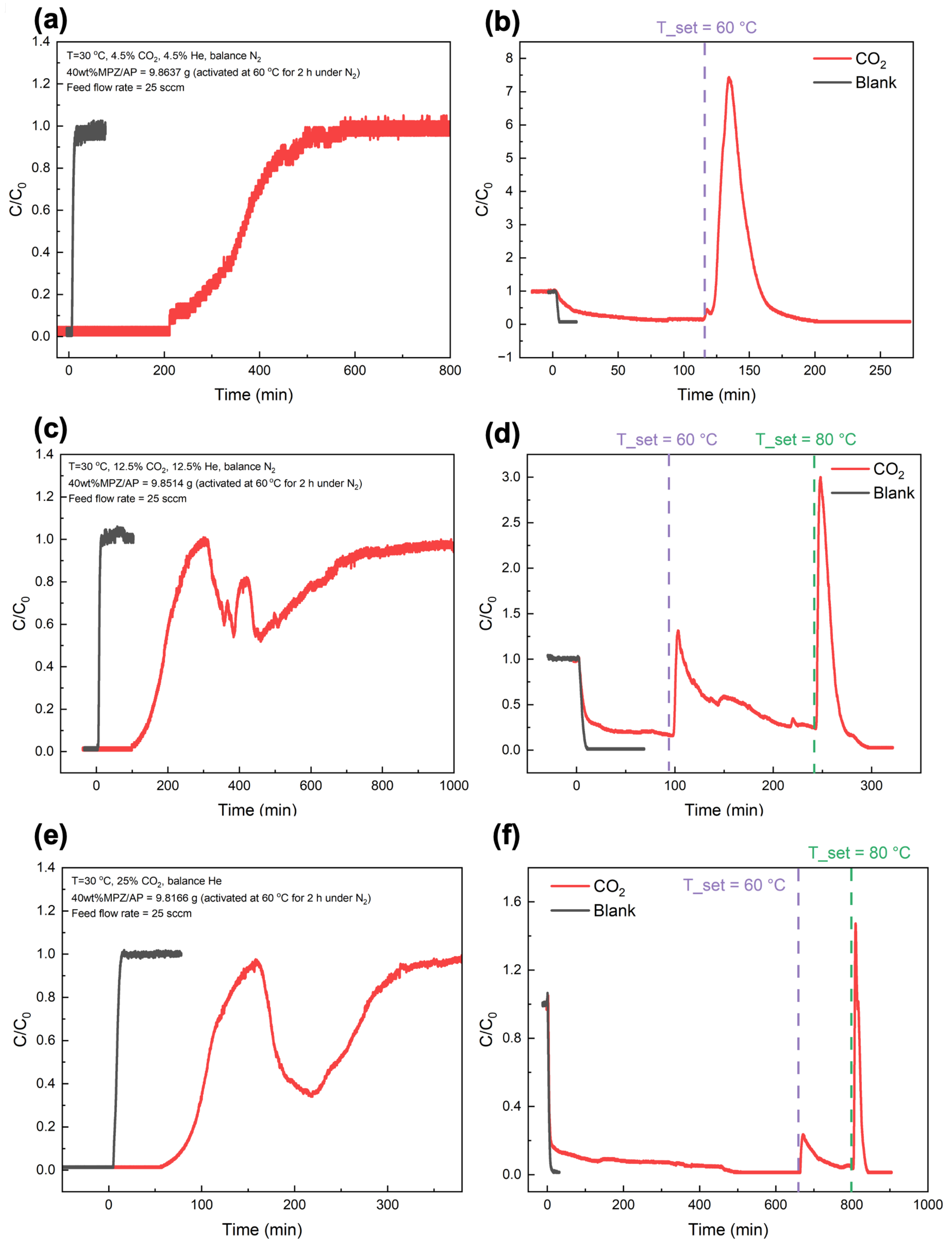

Figure S19. Breakthrough profile of 40 wt.% MPZ/AP with CO_2 concentrations of 4.5%, 12.5% and 25%. (a/c/e) absorption; (b/d/f) desorption. Temperature = 30℃, Pressure = 1 atm.

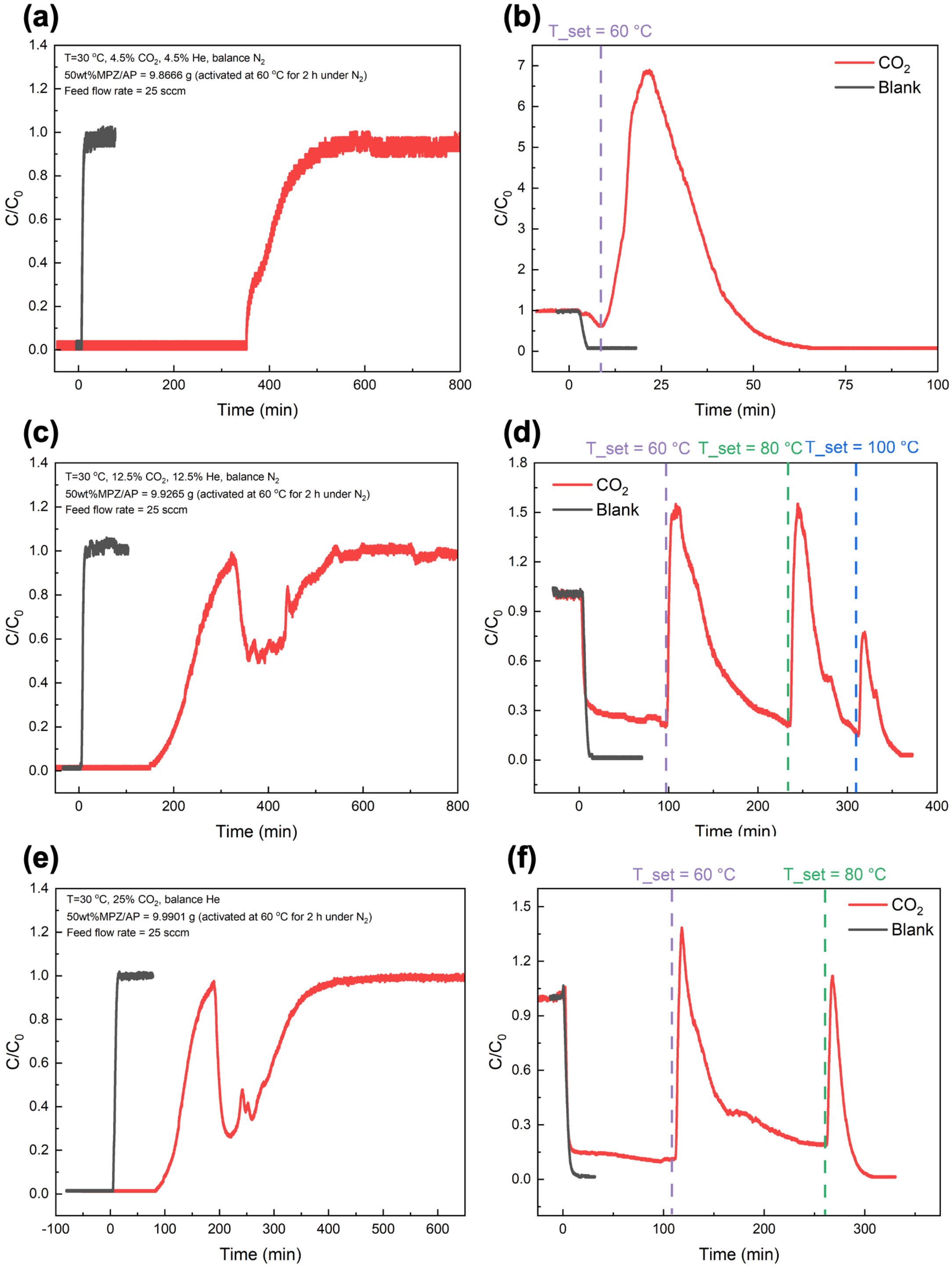

Figure S20. Breakthrough profile of 50 wt.% MPZ/AP with CO_2 concentrations of 4.5%, 12.5% and 25%. (a/c/e) absorption; (b/d/f) desorption. Temperature = 30℃, Pressure = 1 atm.

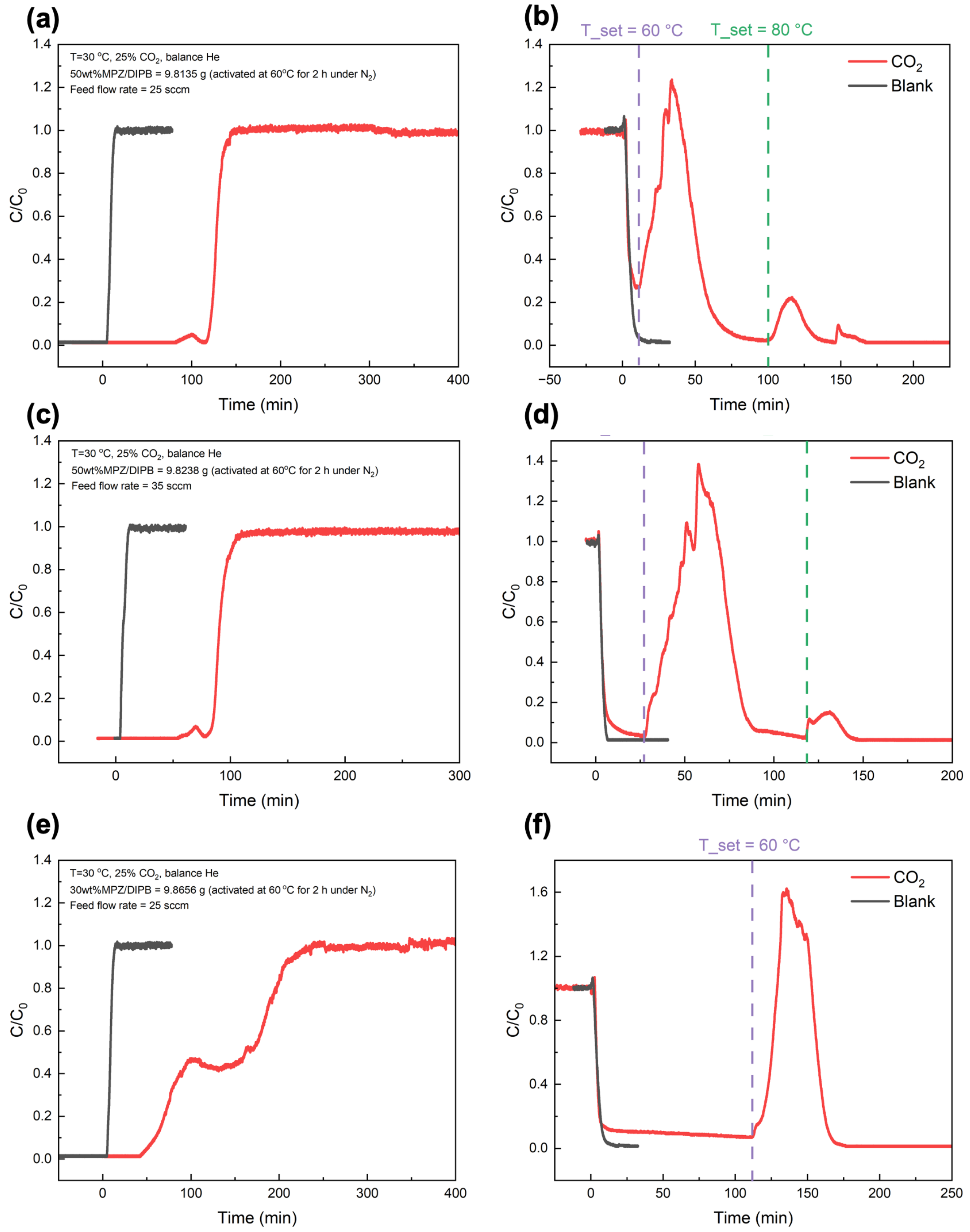

Figure S21. Breakthrough profile of 30 and 50 wt.% MPZ/DIPB with 25% CO_2 concentrations. (a/c/e) absorption; (b/d/f) desorption. Temperature = 30℃, Pressure = 1 atm.

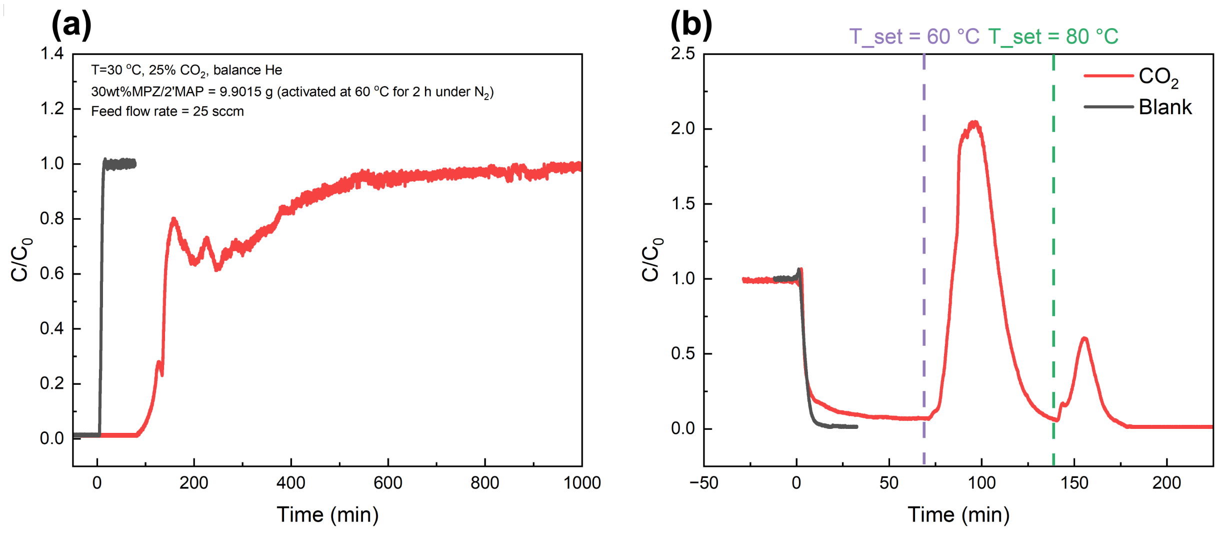

Figure S22. Breakthrough profile of 50 wt.% MPZ/2'MAP with 25% CO_2 concentrations. (a) absorption; (b) desorption. Temperature = 30℃, Pressure = 1 atm.

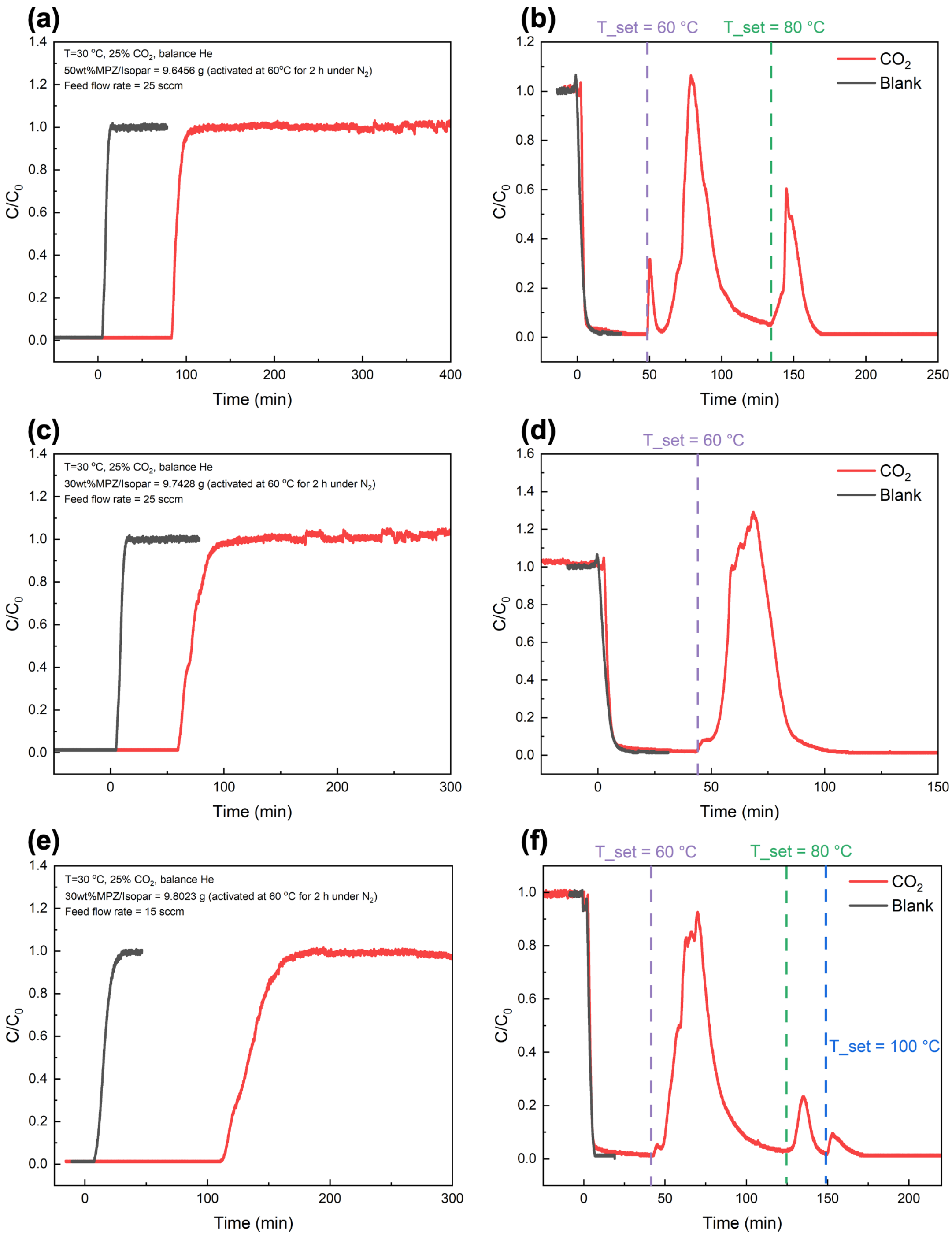

Figure S23. Breakthrough profile of 30 and 50 wt.% MPZ/Isopar with 25% CO_2 concentrations. (a/c/e) absorption; (b/d/f) desorption. Temperature = 30℃, Pressure = 1 atm.

SI.6. NMR analysis

One- and two-dimensional NMR of MPZ confirmed the expected ^{1}H and ^{13}C peak assignments for this molecule (**Figure S24**). Those assignments – particularly the more downfield position of the C3 methylene relative to C2 – aided the interpretation of the spectra obtained for mixtures in the presence of ^{13}C-enriched and non-enriched CO_2.

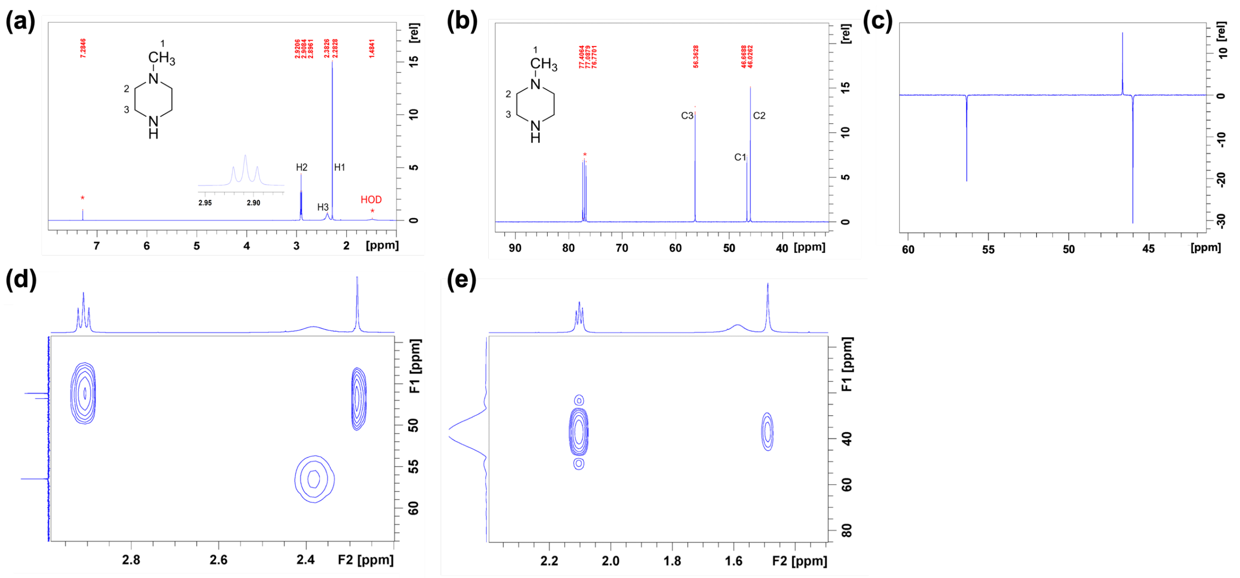

Figure S24. (a) ^{1}H NMR spectrum of 0.2 M MPZ in $CDCl_3$; (b) ^{13}C NMR spectrum of 0.2 M MPZ in $CDCl_3$; (c) DEPT135 ^{13}C NMR spectrum of 0.2 M MPZ in $CDCl_3$; (d) ^{1}H – ^{13}C HSQC spectrum of 6 M MPZ in $CDCl_3$; (e) ^{1}H – ^{15}N HMBC spectrum of 6 M MPZ in $CDCl_3$.

Figures S25-S37 show ^{13}C NMR spectra for various MPZ/additive mixtures before and after exposure to $^{12}CO_2$ or $^{13}CO_2$. In most cases, the solid/liquid mixture or gel-like material formed after exposure to CO_2 was removed and dissolved in $CDCl_3$ for NMR analysis. **Figure S29** (of an MPZ/2'HAP material) shows that MPZ and MPZ-CO_2 adducts are primarily sequestered in the gel/solid phase after CO_2 exposure, as the liquid phase was mostly 2'HAP.

Features common to most of these data include the appearance of a single new and strong ^{13}C signal at approximately 161-162 ppm upon exposure to $^{13}CO_2$. The invariant nature of the chemical shift of this signal suggests that the same species is formed in all cases, presumably some form of the carbamate monomer (MC) or dimer (M_2C_2). The C3 (≈ 54 ppm) resonance of MPZ was found to split into two broader signals upon CO_2 binding in a pattern that correlated well with performance (**Figure S25**). Thus, 50 wt.% mixtures of MPZ with AP, 2'HAP, 2'MAP, and DIPB all exhibited very broad signals of approximately equal intensity after CO_2 treatment, and these preparations absorbed nearly the maximum amount of CO_2 (0.85-0.95 equiv. relative to MPZ) in a two-step pressure-dependent pattern.

In contrast, 50 wt.% mixtures of MPZ with 3'HAP, 4'HAP, and 2CP exhibited moderately-broadened C3 signals of unequal intensity (>3:1, with the upfield signal being stronger), and these materials absorbed less than 0.5 equivalents of CO_2 per MPZ molecule. This spectral feature of MPZ/2IPP was intermediate, showing the narrower signals but in more equal intensities, and its performance was intermediate as well (0.66 equiv. CO_2 per MPZ). Neat MPZ was an exception, showing an NMR pattern (two broad peaks near 53 ppm of equal intensity) characteristic of high-capacity formulations, but molar uptake capacity only

slightly better than 0.5 equiv. CO_2 per MPZ. It is worth noting that only moderate peak broadening, and no peak splitting, was observed for 10wt.% MPZ in 2’HAP, even though this mixture provided efficient CO_2 sorption per unit base. These observations suggest that C3 peak broadening and splitting may be due to a bimolecular (or higher-order) interaction, suggesting that one of the species observed is an intermolecular complex. Note that separate signals for protonated MPZ-H^+ and the freebase MPZ are not seen in the NMR spectra due to rapid proton exchange.

The appearance of the C2 methylene signal (near 45 ppm) after CO_2 binding did not correlate at all with capacity or pressure dependence. Several preparations (50 wt.% MPZ in 2’HAP, 2IPP, 3’HAP, 4’HAP, and 2CP) all showed a second upfield peak of minor, but varying, intensity, but these mixtures exhibited divergent performance. The same can be said about those mixtures that showed a single, moderately broadened, ^{13}C resonance for this site (MPZ neat and 50 wt.% in AP, 2’MAP, and DIPB).

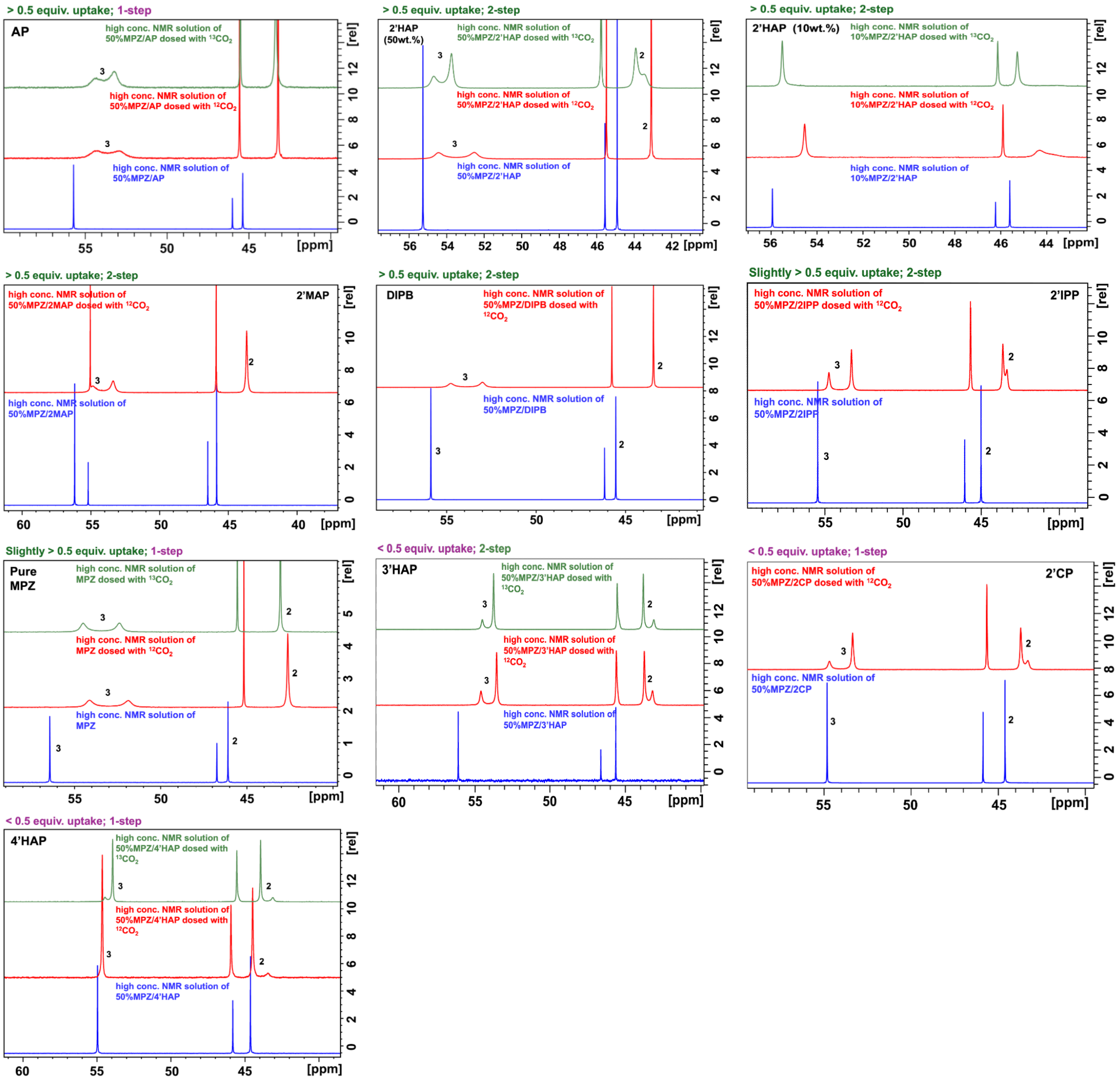

Figure S25. Expanded views of the upfield region of ^{13}C NMR spectra of MPZ/additive mixtures before and after exposure to 1 atm. CO_2 (taken from Figures S16-S26). The additive is indicated in black text in the upper left- or right-hand corner of each plot. Above each plot is a summary of the performance of that mixture: "equiv. uptake" referring to the molar ratio of trapped CO_2 vs. MPZ at the highest CO_2 concentration tested (determined by isotherm measurements); "step" referring to whether or not the uptake-vs-pressure isotherm trace appears as a single step curve or an S-shaped (two-step) curve. "High" concentration is approximately 5M in $CDCl_3$.

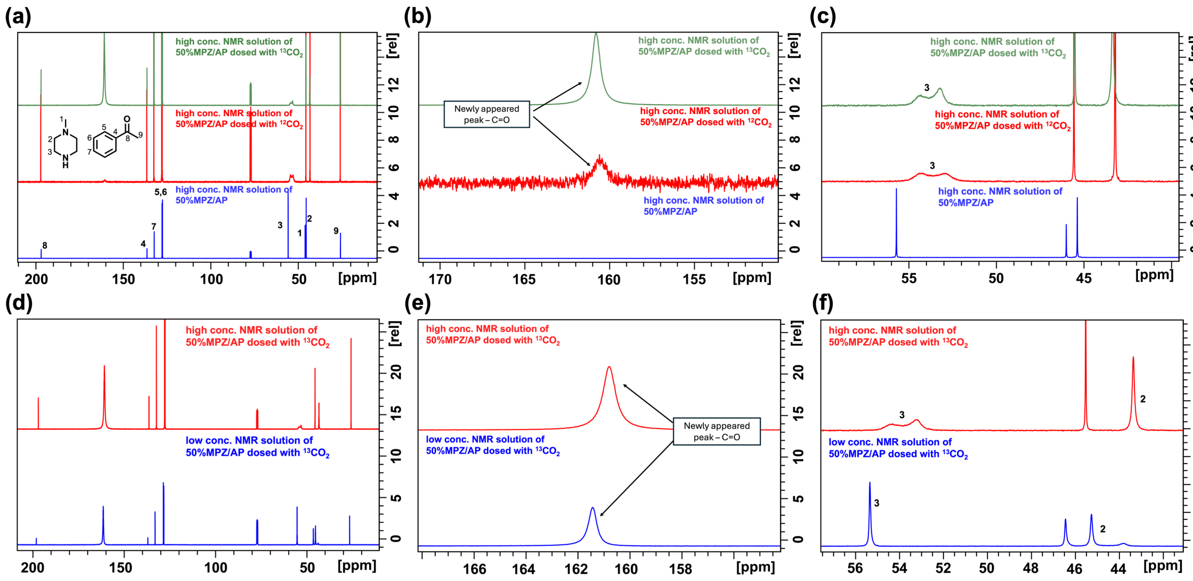

Figure S26. ^{13}C NMR spectra of (a) 50 wt.% MPZ/AP before and after dosing with $^{12}CO_2$/$^{13}CO_2$ in $CDCl_3$. (b,c) Zoomed-in spectra from panel (a). (d) Low and high concentrations of 50 wt.% MPZ/AP dosed with $^{13}CO_2$ in $CDCl_3$. (e,f) Zoomed-in spectra from panel (d). "Low" and "high" concentrations are approximately 1M and 5M solutions in $CDCl_3$, respectively.

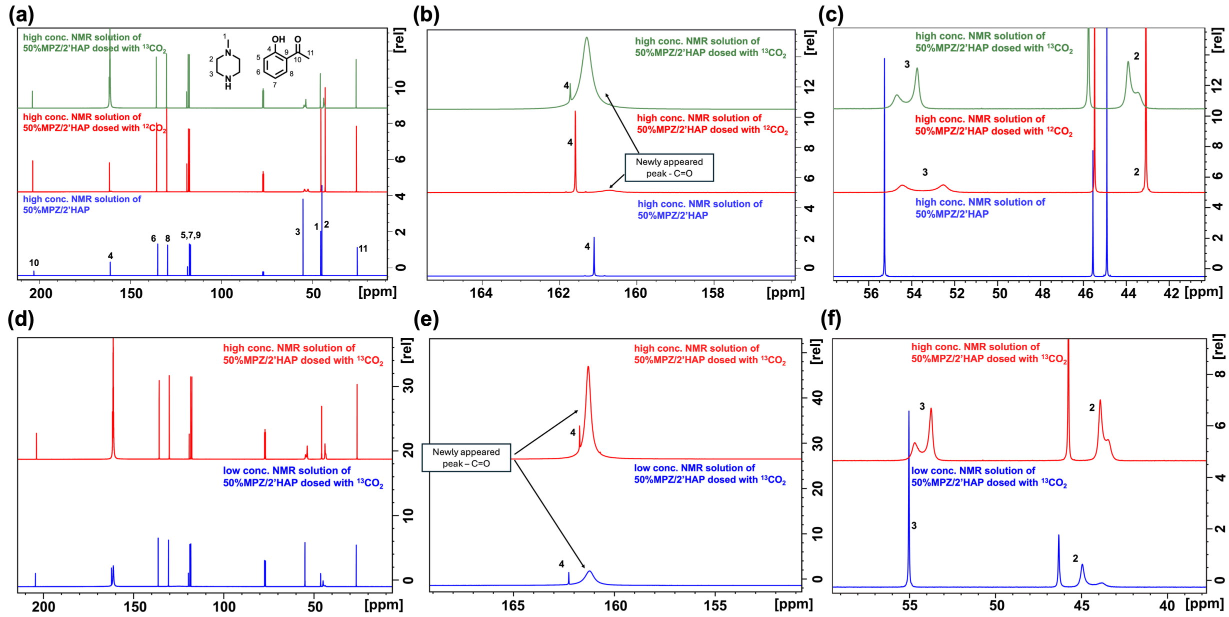

Figure S27. ^{13}C NMR spectra of (a) 50 wt.% MPZ/2'HAP before and after dosing with $^{12}CO_2$/$^{13}CO_2$ in $CDCl_3$. (b,c) Zoomed-in spectra from panel (a). (d) Low and high concentrations of 50 wt.% MPZ/2'HAP dosed with $^{13}CO_2$ in $CDCl_3$. (e,f) Zoomed-in spectra from panel (d). "Low" and "high" concentrations are approximately 1M and 5M solutions in $CDCl_3$, respectively. The spectra in panel (a) do not change at temperatures down to –50°C in $CDCl_3$ (not shown).

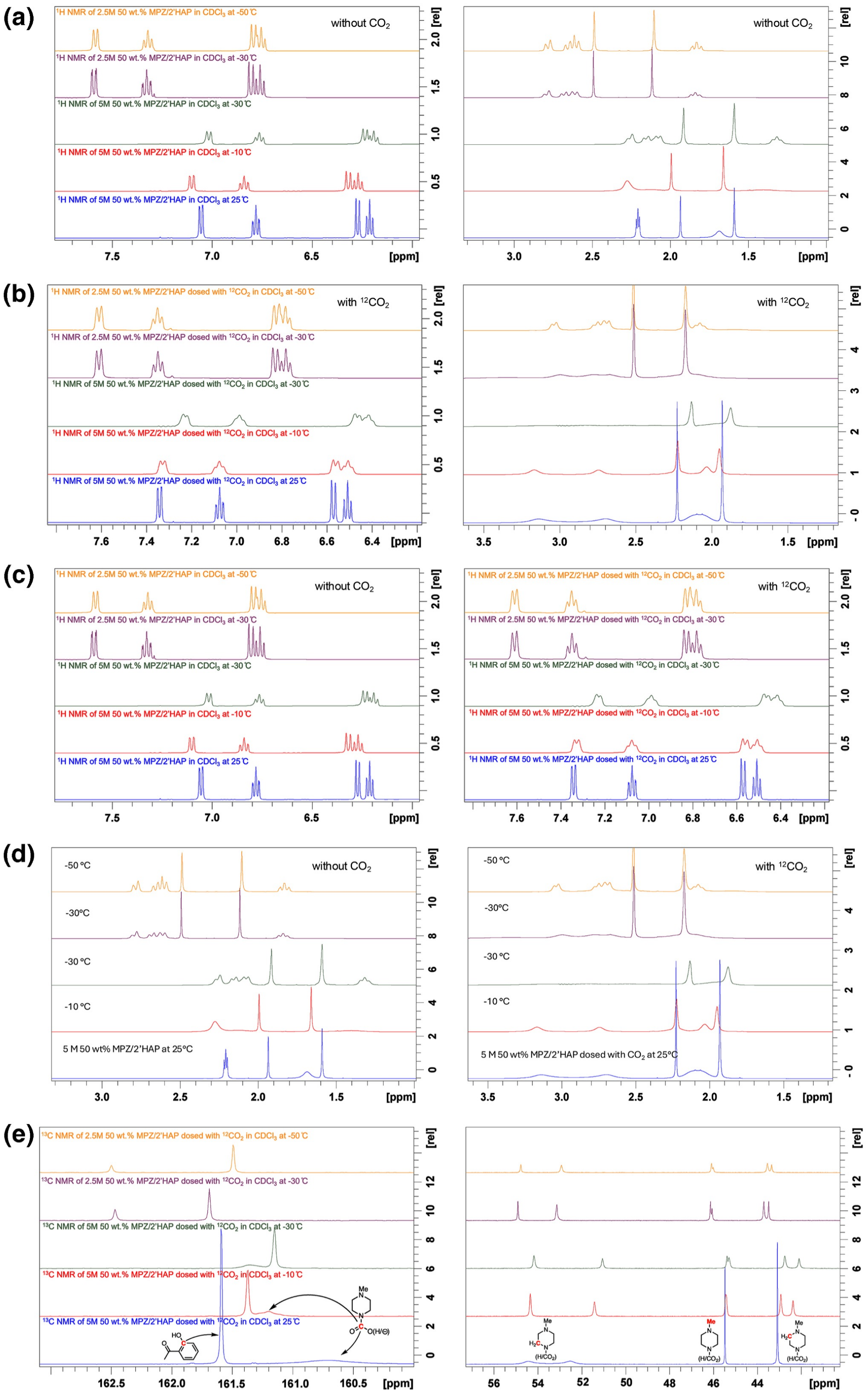

Figure S28. Low-temperature ^{1}H and ^{13}C NMR spectra in the presence and absence of CO_2.

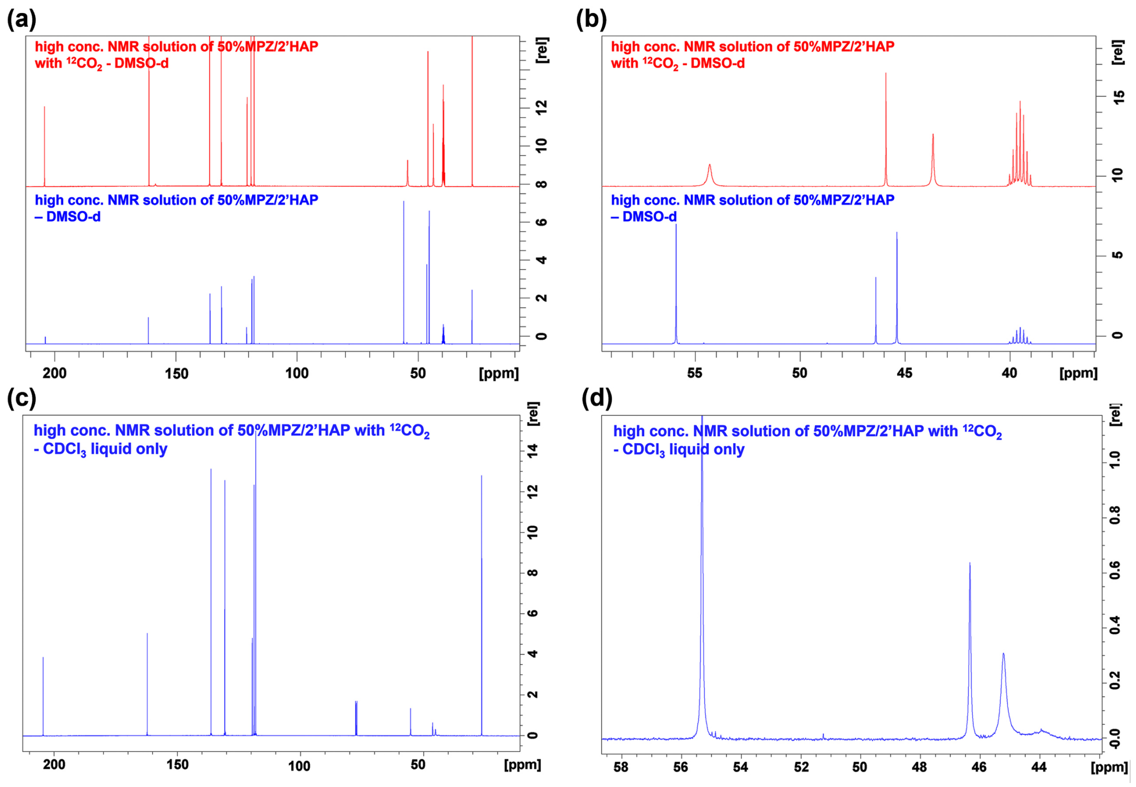

Figure S29. ^{13}C NMR spectra of high concentration (≈5 M in $CDCl_3$) of 50 wt.% MPZ/2'HAP (a) before and after dosing with $^{12}CO_2$ in DMSO-d_6. (b) Zoomed-in spectra from panel (a). (c) Liquid phase of this mixture after dosing with $^{12}CO_2$ in $CDCl_3$. (d) Zoomed-in spectra from panel (c).

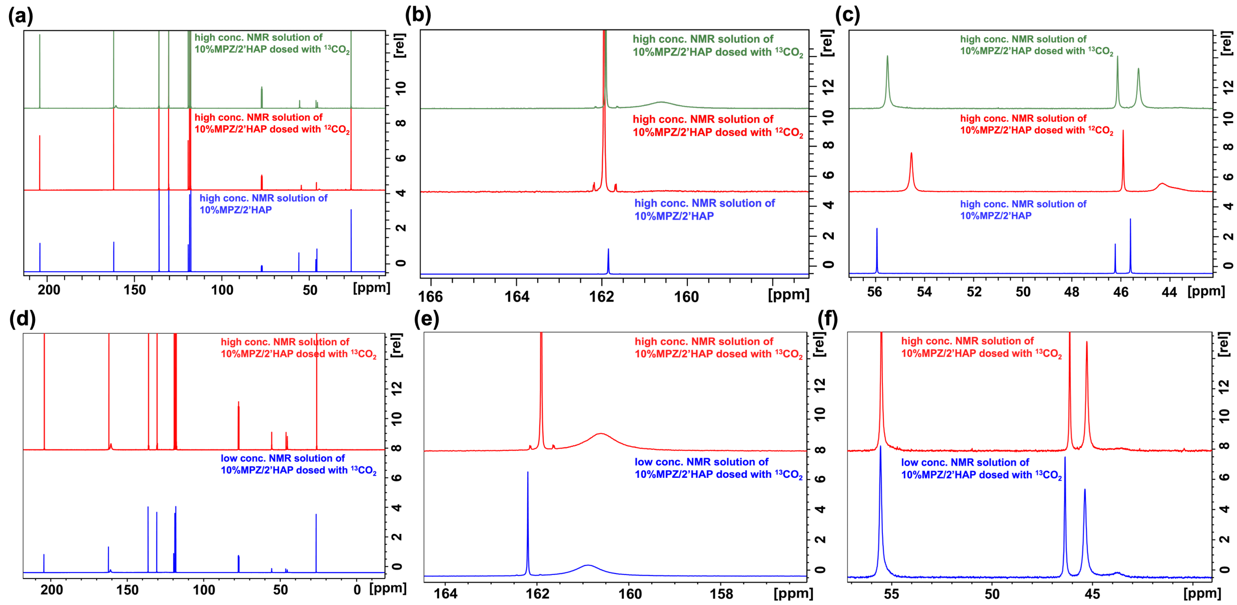

Figure S30. ^{13}C NMR spectra of (a) 10 wt.% MPZ/2'HAP before and after dosing with $^{12}CO_2$/$^{13}CO_2$ in $CDCl_3$. (b/c) Zoomed-in spectra from panel (a). (d) Low and high concentrations of 10 wt.% MPZ/2'HAP dosed with $^{13}CO_2$ in $CDCl_3$. (e/f) Zoomed-in spectra from panel (d). "Low" and "high" concentrations are approximately 1M and 5M solutions in $CDCl_3$, respectively.

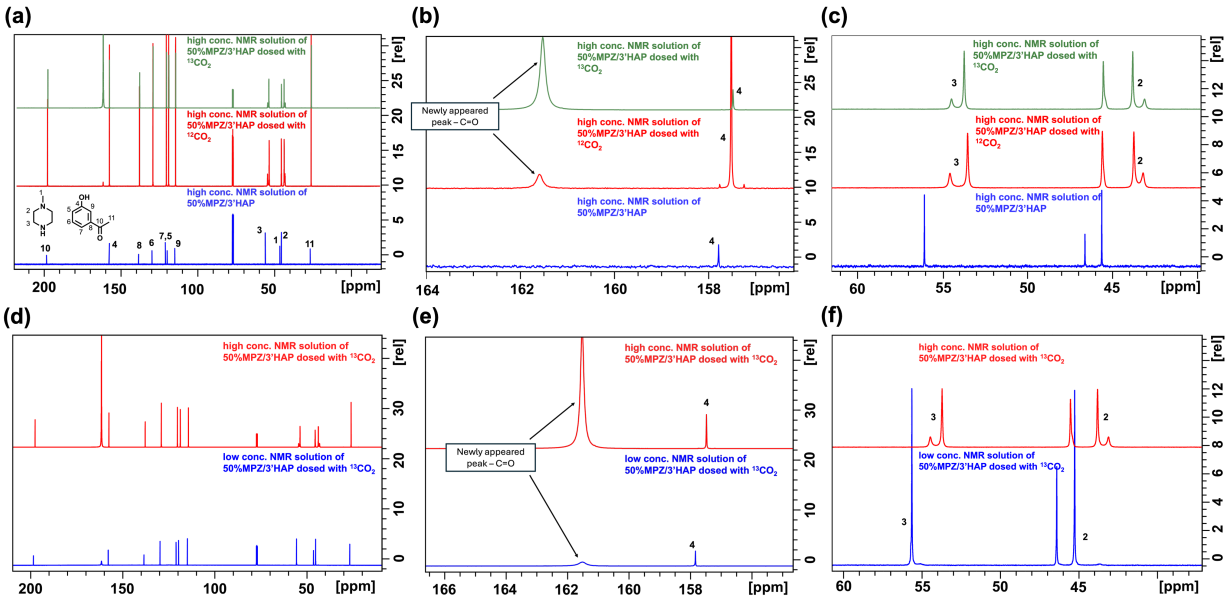

Figure S31. ^{13}C NMR spectra of (a) 50 wt.% MPZ/3'HAP before and after dosing with $^{12}CO_2$/$^{13}CO_2$ in $CDCl_3$. (b/c) Zoomed-in spectra from panel (a). (d) Low and high concentrations of 50 wt.% MPZ/3'HAP dosed with $^{13}CO_2$ in $CDCl_3$. (e/f) Zoomed-in spectra from panel (d). "Low" and "high" concentrations are approximately 1M and 5M solutions in $CDCl_3$, respectively.

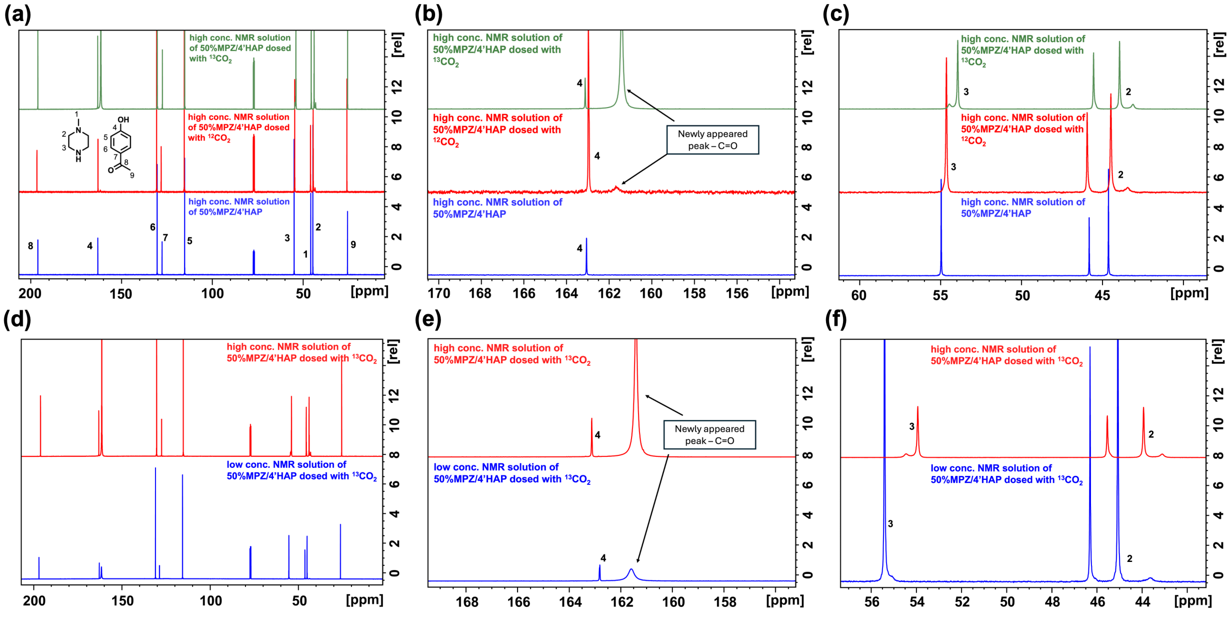

Figure S32. ^{13}C NMR spectra of (a) 50 wt.% MPZ/4'HAP before and after dosing with $^{12}CO_2$/$^{13}CO_2$ in $CDCl_3$. (b/c) Zoomed-in spectra from panel (a). (d) Low and high concentrations of 50 wt.% MPZ/4'HAP dosed with $^{13}CO_2$ in $CDCl_3$. (e/f) Zoomed-in spectra from panel (d). "Low" and "high" concentrations are approximately 1M and 5M solutions in $CDCl_3$, respectively.

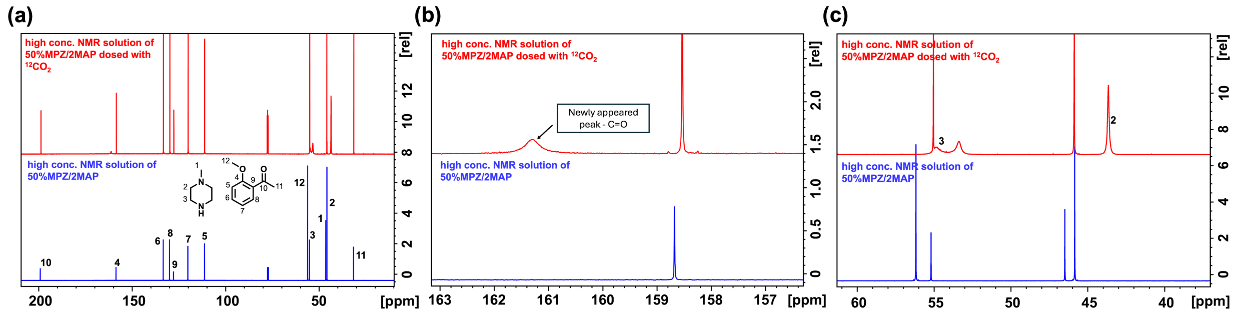

Figure S33. ^{13}C NMR spectra of (a) 50 wt.% MPZ/2'MAP before and after dosing with $^{12}CO_2$ in $CDCl_3$. (b,c) Zoomed-in spectra from panel (a).

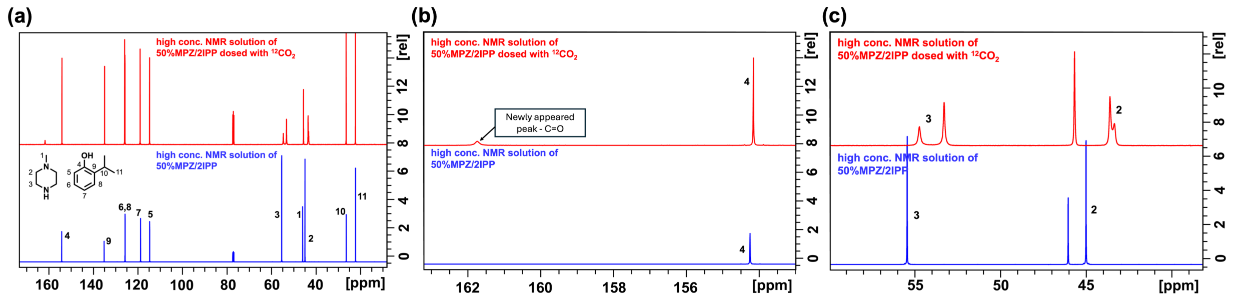

Figure S34. ^{13}C NMR spectra of (a) 50 wt.% MPZ/2IPP before and after dosing with $^{12}CO_2$ in $CDCl_3$. (b/c) Zoomed-in spectra from panel (a).

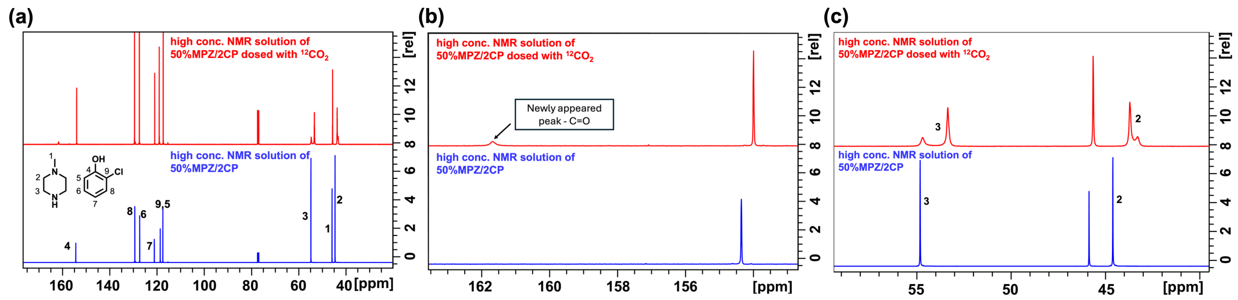

Figure S35. ^{13}C NMR spectra of (a) 50 wt.% MPZ/2CP before and after dosing with $^{12}CO_2$ in $CDCl_3$. (b/c) Zoomed-in spectra from panel (a).

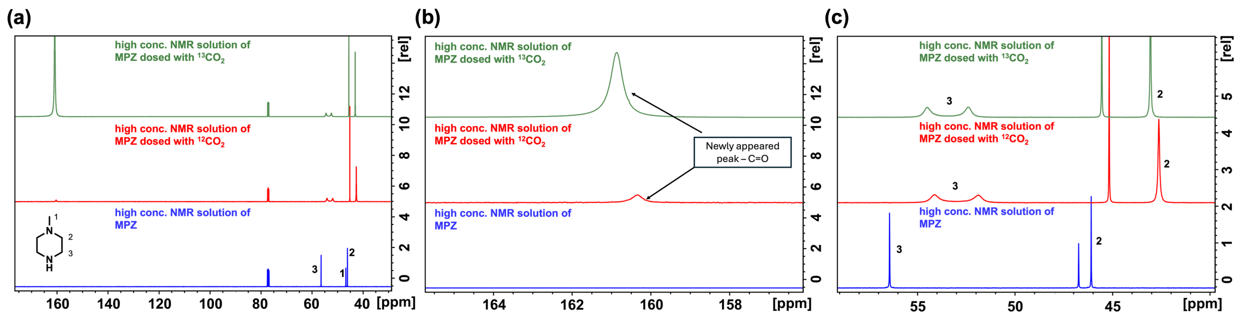

Figure S36. ^{13}C NMR spectra of (a) MPZ before and after dosing with $^{12}CO_2$/$^{13}CO_2$ in $CDCl_3$. (b/c) Zoomed-in spectra from panel (a).

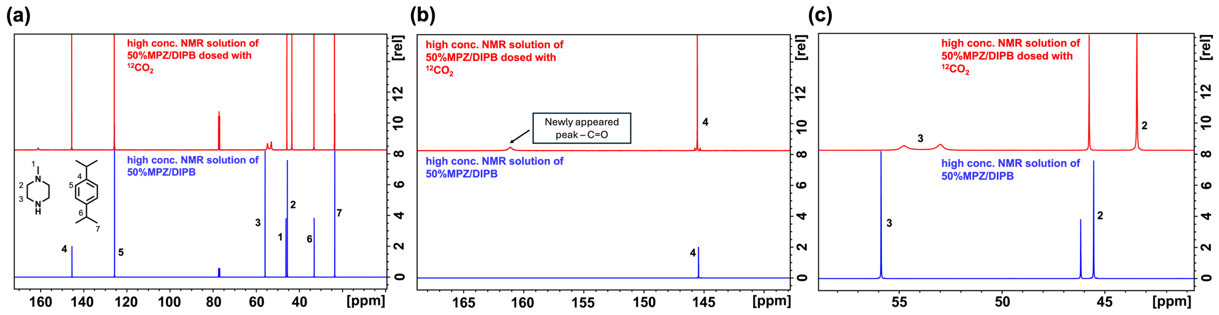

Figure S37. ^{13}C NMR spectra of (a) 50 wt.% MPZ/DIPB before and after dosing with $^{12}CO_2$ in $CDCl_3$. (b/c) Zoomed-in spectra from panel (a).

SI.7. Steady-State Equilibrium Analysis of MPZ–CO_2 Reaction Networks

SI.7.1 MPZ in Low-Polarity or Solvent-Free Conditions (Model 1)

Here we consider the reversible interactions shown in Model 1 in Fig. 8, which involve only MPZ and CO_2, appropriate for the neat base or solvents that are expected to be inert.

We consider the following reversible reactions (**Fig. 8**); the chemical species in bold represent various conjugates of M with C:

$$M + C_0 \rightleftharpoons \boldsymbol{MC} \quad \text{with } K_1 \tag{A1}$$

$$M + \boldsymbol{MC} \rightleftharpoons \boldsymbol{M_2C} \quad \text{with } K_2 \tag{A2}$$

$$\boldsymbol{MC} + \boldsymbol{MC} \rightleftharpoons \boldsymbol{M_2C_2} \quad \text{with } K_3 \tag{A3}$$

$$[C] = [C]_0 = \text{externally maintained constant through continuous } CO_2 \text{ flow}$$

To define the system in terms of [M] and $[C]_0$, from Eq. A1-A3 we obtain,

$$\boldsymbol{MC} = K_1 M C_0 \ = \ \alpha M \tag{A4}$$

$$\boldsymbol{M_2C} = K_2 M \cdot \boldsymbol{MC} = K_2 K_1 M^2 C_0 \ = \ \beta M^2 \tag{A5}$$

$$\boldsymbol{M_2C_2} = K_3 (\boldsymbol{MC})^2 = K_3 K_1^2 M^2 C_0^2 \ = \ \gamma M^2 \tag{A6}$$

Since the total concentration of M (M_{tot}) is constant, at equilibrium,

$$M_{\text{tot}} = M + \boldsymbol{MC} + 2\boldsymbol{M_2C} + 2\boldsymbol{M_2C_2} \tag{A7}$$

Plugging in the expressions of **MC**, $\boldsymbol{M_2C}$, $\boldsymbol{M_2C_2}$ from Eq. A4-A6 into Eq. A7, we obtain,

$$2(\beta+\gamma)M^2 \ + \ M(1+\alpha) - M_{tot} = 0 \tag{A8}$$

This is a quadratic equation in M with

$$A_1 M^2 + A_2 M + A_3 = 0 \tag{A9}$$

where,

$$A_1 = 2(\beta + \gamma) \tag{A10}$$

$$A_2 = (1 + \alpha) \tag{A11}$$

$$A_3 = -M_{\text{tot}} \quad (A12)$$

Since, Eq. A9 is quadratic in *M*, it has 2 roots, of which only the real positive value is of interest. Solving Eq. A9 yields,

$$M = \frac{-A_2 + \sqrt{\left(A_2{}^2 - 4A_1A_3\right)}}{2A_1} \text{ (considering positive root only)} \quad (A13)$$

Plugging in expressions of A_1, A_2, and A_3 from Eq. A10-A12 into Eq. A13, we obtain,

$$M = \frac{-(1+\alpha) + \sqrt{(1+\alpha)^2 + 8(\beta+\gamma)M_{tot}}}{4(\beta+\gamma)}$$
$$= \frac{-C_0K_1 - 1 + \sqrt{M_{\text{tot}}(8K_3C_0^2K_1^2 + 8K_2C_0K_1) + (C_0K_1+1)^2}}{4K_3C_0^2K_1^2 + 4K_2C_0K_1} \quad (A14)$$

Now plugging in the expression of *M* from Eq. A14 into the expression of *MC* in Eq. A4, we obtain,

$$[\boldsymbol{MC}] = K_1MC_0 = K_1C_0\frac{-C_0K_1 - 1 + \sqrt{M_{\text{tot}}(8K_3C_0^2K_1^2 + 8K_2C_0K_1) + (C_0K_1+1)^2}}{4K_3C_0^2K_1^2 + 4K_2C_0K_1}$$
$$= \frac{-C_0K_1 - 1 + \sqrt{M_{\text{tot}}(8K_3C_0^2K_1^2 + 8K_2C_0K_1) + (C_0K_1+1)^2}}{4K_3K_1C_0 + 4K_2} \quad (A15)$$

Similarly plugging in the expression of *M* from Eq. A14 into the expression of M_2C in Eq. A5, we obtain,

$$[\boldsymbol{M_2C}] = \frac{C_0K_1K_2\left(-C_0K_1 - 1 + \sqrt{M_{\text{tot}}(8K_3C_0^2K_1^2 + 8K_2C_0K_1) + (C_0K_1+1)^2}\right)^2}{(4K_3C_0^2K_1^2 + 4K_2C_0K_1)^2} \quad (A16)$$

Finally, M_2C_2 reads as,

$$[\boldsymbol{M_2C_2}] = \frac{C_0^2K_1^2K_3\left(-C_0K_1 - 1 + \sqrt{M_{\text{tot}}(8K_3C_0^2K_1^2 + 8K_2C_0K_1) + (C_0K_1+1)^2}\right)^2}{(4K_3C_0^2K_1^2 + 4K_2C_0K_1)^2} \quad (A17)$$

Next, we determine at what C_0 concentration, M_2C is maximized. This is obtained by setting,

$$\frac{d[\boldsymbol{M_2C}]}{dC_0} = \frac{d}{dC_0}(K_2K_1M^2C_0) = 0 \quad (A18)$$

Under the assumption $K_3 = 0$,

$$\frac{d[\boldsymbol{M_2C}]}{dC_0} = \left(\frac{K_1 - \frac{2K_1(C_0K_1+1)+8K_1K_2M_{\text{tot}}}{2\sqrt{(C_0K_1+1)^2+8C_0K_1K_2M_{\text{tot}}}}}{8C_0K_1K_2}\right)\left(C_0K_1 - \sqrt{(C_0K_1+1)^2+8C_0K_1K_2M_{\text{tot}}} + 1\right) - \frac{\left(C_0K_1 - \sqrt{(C_0K_1+1)^2+8C_0K_1K_2M_{\text{tot}}} + 1\right)^2}{16C_0^2K_1K_2} \quad (A19)$$

Setting $\frac{d[M_2C]}{dC_0} = 0$, we find that M_2C maximizes at a critical value of

$$C_0 = \frac{1}{K_1} \quad \text{(A20)}$$

Net CO_2 uptake

For specific values of K_1, K_2, K_3 and C_0, the net CO_2 uptake at equilibrium is given by,

$$Uptake_{\text{net}} = \boldsymbol{MC}|_{C=C_0} + \boldsymbol{M_2C}|_{C=C_0} + \boldsymbol{2M_2C_2}|_{C=C_0} \quad (A21)$$

SI.7.2 MPZ in non-innocent, non-protic additives (Model 2)

The additives 2'-methoxyacetophenone (2'MAP) and 1,4-diisopropylbenzene (DIPB) provided high-capacity two-phase sorption behavior, but lack an acidic proton. We therefore modeled this type of system using the reversible interactions shown in Model 2 in **Fig. 8**.

We consider the following reversible reactions (**Fig. 8**):

$$M + C_0 \rightleftharpoons \boldsymbol{MC} \quad \text{with } K_1 \quad (B1)$$

$$M + \boldsymbol{MC} \rightleftharpoons \boldsymbol{M_2C} \quad \text{with } K_2 \quad (B2)$$

$$\boldsymbol{MC} + \boldsymbol{MC} \rightleftharpoons \boldsymbol{M_2C_2} \quad \text{with } K_3 \quad (B3)$$

$$\boldsymbol{MC} + H \rightleftharpoons \boldsymbol{MCH} \quad \text{with } K_4 \quad (B4)$$

$$\boldsymbol{M_2C_2} + H \rightleftharpoons \boldsymbol{M_2C_2H} \quad \text{with } K_5 \quad (B5)$$

<u>*Step 1: Define Complex Species in terms of M, H, and C_0*</u>

Using equilibrium relationships, from Eq. B1-B3 and Eq. B4-B5, we obtain,

$$\boldsymbol{MC} = K_1MC_0 = \alpha M \quad (B6)$$

$$\boldsymbol{M_2C} = K_2M \cdot \boldsymbol{MC} = K_2K_1M^2C_0 = \beta M^2 \quad (B7)$$

$$\boldsymbol{M_2C_2} = K_3(\boldsymbol{MC})^2 = K_3K_1^2M^2C_0^2 = \gamma M^2 \quad (B8)$$

$$\boldsymbol{MCH} = K_4.\boldsymbol{MC}.H = K_4K_1MC_0H = \delta MH \quad (B9)$$

$$\boldsymbol{M_2C_2H} = K_5.\boldsymbol{M_2C_2}.H = K_5K_3(\boldsymbol{MC})^2H = K_5K_3K_1^2M^2C_0^2H = \zeta M^2H \quad (B10)$$

Step 2: Impose the constraints that total [M] and total [H] are constant

Let the starting concentration of M be M_{tot}. Then, at equilibrium,

$$M_{\mathrm{tot}} = M + \boldsymbol{MC} + 2\boldsymbol{M_2C} + 2\boldsymbol{M_2C_2} + \boldsymbol{MCH} + 2\boldsymbol{M_2C_2H} \qquad (B11)$$

Plugging in the expressions of **MC**, **MCH**, $\mathbf{M_2C}$, $\mathbf{M_2C_2}$, and $\mathbf{M_2C_2H}$ from Eq. B6-B10 into Eq. B11 we obtain,

$$M + \alpha M + 2\beta M^2 + 2\gamma M^2 + \delta MH + 2\zeta M^2 H - M_{tot} = 0 \qquad (B12)$$

Similarly, Let the starting concentration of H be $H_{tot.}$ Then, at equilibrium, using Eq. B9-B10,

$$H_{\mathrm{tot}} = H + \boldsymbol{MCH} + \boldsymbol{M_2C_2H} = H + \delta MH + \zeta M^2 H \qquad (B13)$$

Upon algebraic manipulation, from Eq. B13 we obtain,

$$H = \frac{H_{tot}}{1 + M(\delta + \zeta M)} \qquad (B14)$$

Substituting H from Eq. B14 into Eq. B12, and rearranging, we get a 4th order polynomial in M,

$$A_4M^4 + A_3M^3 + A_2M^2 + A_1M + A_0 = 0 \qquad (B15)$$

where,

$$A_4 = 2\zeta(\beta + \gamma) \qquad (B16)$$

$$A_3 = 2\delta(\beta + \gamma) + \zeta(1 + \alpha) \qquad (B17)$$

$$A_2 = 2(\beta + \gamma) + \delta(1 + \alpha) - \zeta M_{tot} + 2\zeta H_{tot} \qquad (B18)$$

$$A_1 = (1 + \alpha) + \delta(H_{tot} - M_{tot}) \qquad (B19)$$

$$A_0 = -M_{tot} \qquad (B20)$$

Eq. B15 is quartic in M, thus has 4 roots. We are only interested in the real positive roots of M.

For specific values of equilibrium constants K's and C_0, the net CO_2 uptake at equilibrium is given by

$$Uptake_{net} = \boldsymbol{MC}|_{C=C_0} + \boldsymbol{M_2C}|_{C=C_0} + 2\boldsymbol{M_2C_2}|_{C=C_0} + 2\boldsymbol{M_2C_2H}|_{C=C_0} + \boldsymbol{MCH}|_{C=C_0} \qquad (B21)$$

SI.7.3 MPZ in phenolic additives (Model 3)

It is apparent from our experimental results that the nature of the compound in which MPZ is dissolved (the "additive") makes a considerable difference to the performance of the base in these systems. If the additives were really used in large molar excess relative to MPZ, we could model these systems in the same manner as above and simply assign different values of equilibrium constants, which would indeed be expected to be solvent-dependent. However, the molar amounts of additives and MPZ were not very different in many mixtures tested, and so it should be more accurate to model the effects of additives by positing discrete (and concentration-dependent) stabilizing interactions between additive and MPZ-CO_2 species. Furthermore, the hydroxyacetophenones explored here have acidities that are in the range of MPZ basicities, and therefore acid-base equilibria are likely to be important. We therefore consider the reversible interactions shown in Model 3 in Fig. 8.

We consider the following reversible reactions (Figure **8**).

$$M + C_0 \rightleftharpoons \boldsymbol{MC} \quad \text{with } K_1 \tag{C1}$$

$$M + \boldsymbol{MC} \rightleftharpoons \boldsymbol{M_2C} \quad \text{with } K_2 \tag{C2}$$

$$\boldsymbol{MC} + \boldsymbol{MC} \rightleftharpoons \boldsymbol{M_2C_2} \quad \text{with } K_3 \tag{C3}$$

$$\boldsymbol{MC} + H \rightleftharpoons \boldsymbol{MCH} \quad \text{with } K_4 \tag{C4}$$

$$\boldsymbol{M_2C_2} + H \rightleftharpoons \boldsymbol{M_2C_2H} \quad \text{with } K_5 \tag{C5}$$

$$M + H \rightleftharpoons \boldsymbol{MH} \quad \text{with } K_6 \tag{C6}$$

Step 1: Define Complex Species in terms of M, H, and C_0

Using equilibrium relationships, from Eq. C1-C3 and Eq. C4-C5, we obtain,

$$\boldsymbol{MC} = K_1 M C_0 \ = \ \alpha M \tag{C7}$$

$$\boldsymbol{M_2C} = K_2 M \cdot \boldsymbol{MC} = K_2 K_1 M^2 C_0 \ = \ \beta M^2 \tag{C8}$$

$$\boldsymbol{M_2C_2} = K_3 (\boldsymbol{MC})^2 = K_3 K_1^2 M^2 C_0^2 \ = \ \gamma M^2 \tag{C9}$$

$$\boldsymbol{MCH} = K_4 . \boldsymbol{MC} . H = K_4 K_1 M C_0 H \ = \ \delta MH \tag{C10}$$

$$\boldsymbol{M_2C_2H} = K_5 . \boldsymbol{M_2C_2} . H = \ K_5 K_3 (\boldsymbol{MC})^2 H = K_5 K_3 K_1^2 M^2 C_0^2 H \ = \ \zeta M^2 H \tag{C11}$$

$$\boldsymbol{MH} = K_6 . M . H \tag{C12}$$

Step 2: Impose the constraints that total [M] and total [H] are constant

Let the starting concentration of M be M_{tot}. Then, at equilibrium,

$$M_{\text{tot}} = M + \boldsymbol{MC} + 2\boldsymbol{M_2C} + 2\boldsymbol{M_2C_2} + \boldsymbol{MCH} + 2\boldsymbol{M_2C_2H} \ + \ \boldsymbol{MH} \tag{C13}$$

Plugging in the expressions of **MC**, **MCH**, **M_2C**, **M_2C_2**, and **M_2C_2H** from Eq. C7-C12 into Eq. C13 we obtain,

$$M + \alpha M + 2\beta M^2 + 2\gamma M^2 + \delta MH + 2\zeta M^2 H \ + \ K_6 MH \ - \ M_{tot} \ = \ 0 \tag{C14}$$

Similarly, Let the starting concentration of H be $H_{tot.}$ Then, at equilibrium, using Eq. C10-C12,

$$H_{\text{tot}} = H + \boldsymbol{MH} + \boldsymbol{MCH} + \boldsymbol{M_2C_2H} = H \ + K_6 MH \ + \delta MH + \ \zeta M^2 H \tag{C15}$$

Upon algebraic manipulation, from Eq. C15 we obtain,

$$H = \frac{H_{tot}}{1 + (\delta \ + K_6) M \ + \ \zeta M^2} \tag{C16}$$

Substituting H from Eq. C16 into Eq. C13-C14, and rearranging, we obtain a 4th order polynomial in M,

$$A_4 M^4 + A_3 M^3 + A_2 M^2 + A_1 M + A_0 = 0 \tag{C17}$$

where,

$$A_4 = 2\zeta(\beta + \gamma) \tag{C18}$$

$$A_3 = 2(\delta + K_6)(\beta + \gamma) + \zeta(1 + \alpha) \quad (C19)$$

$$A_2 = 2(\beta + \gamma) + (\delta + K_6)(1 + \alpha) - \zeta M_{tot} + 2\zeta H_{tot} \quad (C20)$$

$$A_1 = (1 + \alpha) + (\delta + K_6)(H_{tot} - M_{tot}) \quad (C21)$$

$$A_0 = -M_{tot} \quad (C22)$$

Eq. C17 is quartic in *M*, thus has 4 roots. We are only interested in the real positive roots of *M*.

For specific values of equilibrium constants *K*'s and C_0, the net CO_2 uptake at equilibrium is given by

$$Uptake_{net} = \boldsymbol{MC}|_{C=C_0} + \boldsymbol{M_2C}|_{C=C_0} + 2\boldsymbol{M_2C_2}|_{C=C_0} + 2\boldsymbol{M_2C_2H}|_{C=C_0} + \boldsymbol{MCH}|_{C=C_0} \quad (C23)$$

SI.7.4 Numerical implementation for MPZ equilibria

The equilibrium equations for free MPZ concentration in different network models were solved numerically in MATLAB R2024a (The MathWorks, Natick, MA, USA) using a hybrid global–local strategy. A genetic algorithm (GA) was first applied over segmented intervals of the feasible domain [0, M_{tot}] to minimize the residual $|f(M)| = |A_n M^n + A_{n-1} M^{n-1} + \cdots + A_0|$, identifying candidate roots. These candidates were refined using the **fzero** function to obtain high-precision solutions. When GA was unavailable or failed to converge, fallback options included bracketed **fzero**, local minimization via **fmincon**, and dense grid scanning to ensure robustness. Only real, positive roots with sufficiently low residuals were considered. For quadratic cases (n = 2), numerical roots were benchmarked against analytical closed-form solutions to validate accuracy. In Figure **S38**, we used a hybrid global-local optimization approach to fit Model 1 to the experimental isotherms. We implemented MATLAB's particle swarm optimization with hybrid local refinement via fmincon. The optimization was repeated from multiple randomized initial swarms to ensure robustness of the fitted parameters.

SI.7.5 Representations of Modeling Results

Supplementary Movie 1 shows the calculated distribution of species in the Model 1 scheme (**Fig. 8**), but including values of K_6. Supplementary Movies 2a and 2b show these distributions adding nonzero values for K_3 and K_4, respectively. For all three movies, the equations of Model 3 are employed, setting the omitted equilibria to constant values of 10^{-16} as a numerical representation of zero.

Supplementary Movies 3 and 4 show the calculated distribution of species in the Model 2 and Model 3 schemes (**Fig. 8**) for sets of equilibria varying by a factor of 10 over the following ranges:

$K_1 = 80$, $K_2 = 1 - 10^6$, $K_3 = 1 - 10^6$, $K_4 = 1 - 10^6$, $K_5 = 1 - 10^6$, $K_6 = 1\text{-}100$

For all movies, each frame shows a different combination of equilibrium constants; paging through the frames from start to finish shows how the uptake and speciation patterns vary as K_{eq} values change one at a time.

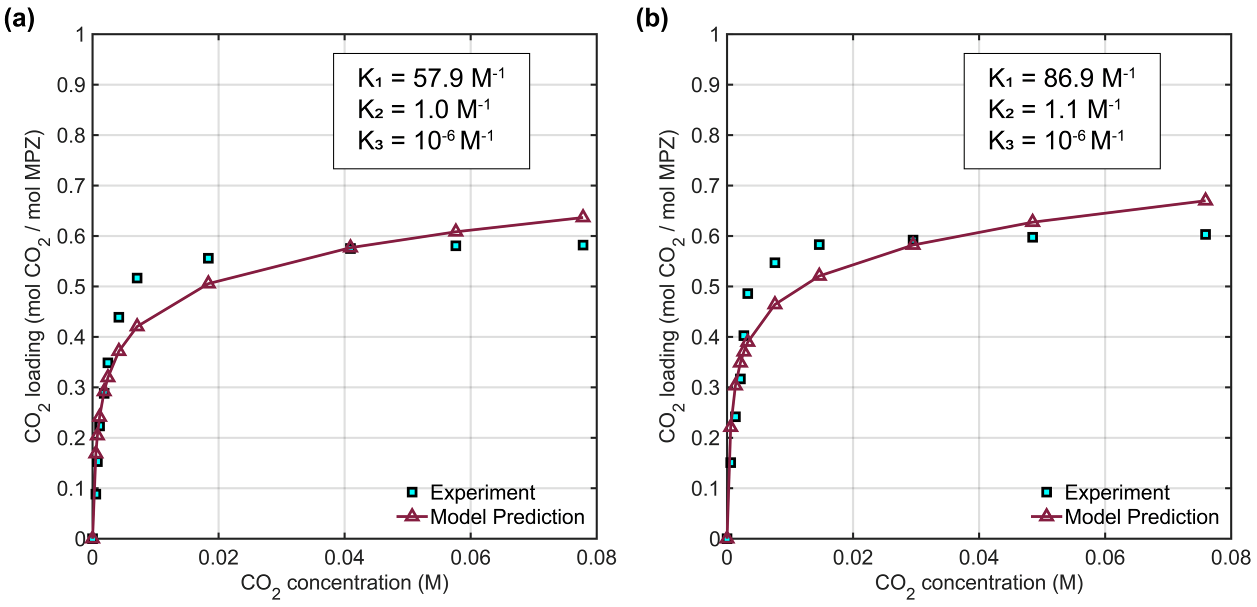

Figure S38. Predicted isotherm plots that fit best to the experimental data (independent runs of Model 1, with K_3 set to 10^{-6} across two experimental replicates).

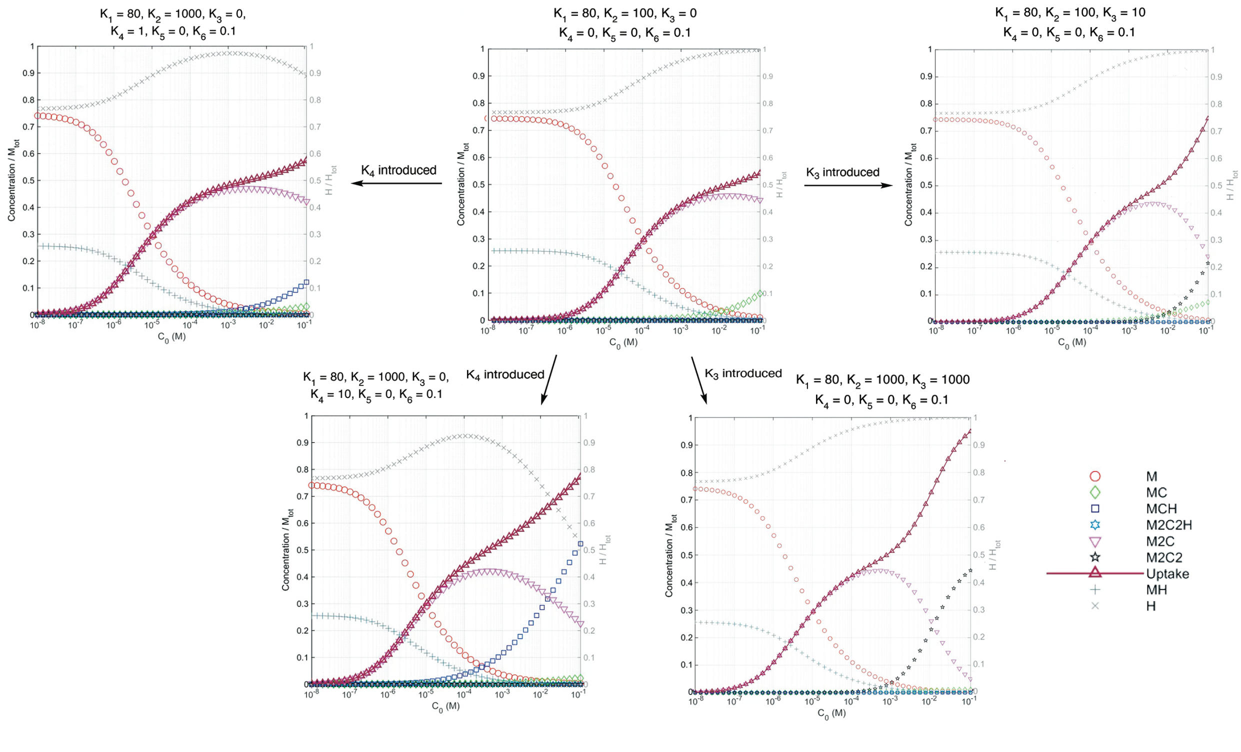

Figure S39. Network plots using only K_1, K_2, and K_6 do not show two-step isotherm patterns. At the top center is a plot with parameters that come closest to two-step patterns upon the introduction of nonzero values for K_3 (right) or K_4 (left).

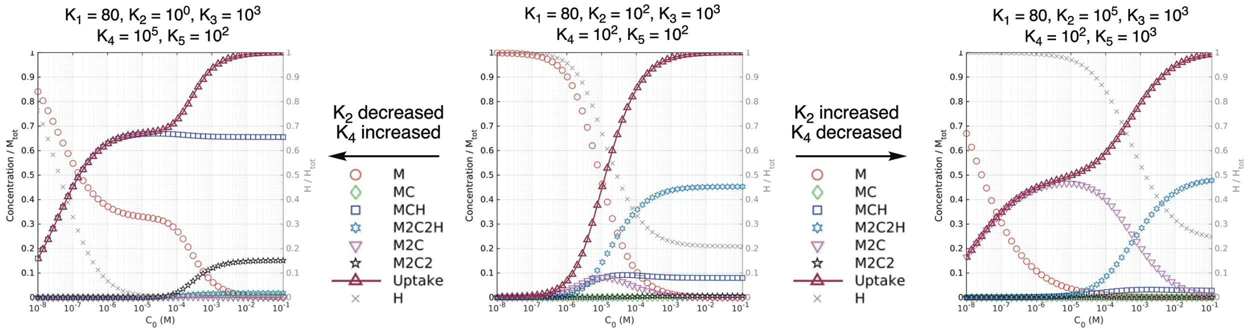

Figure S40. Network plots using Model 2 of CO_2 uptake (molar ratio vs. total MPZ concentration) for 50 weight-% mixtures of MPZ and additive.

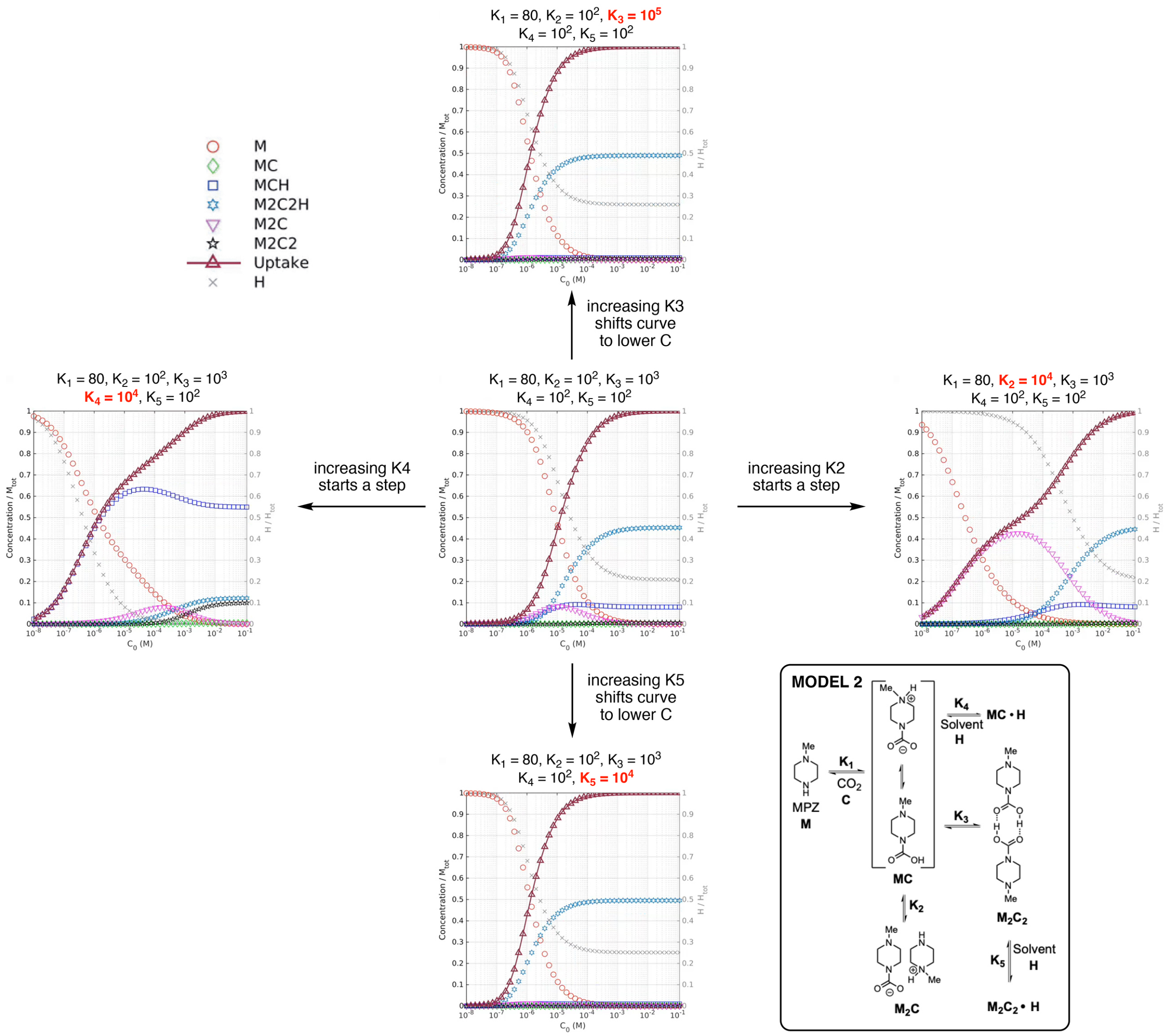

Figure S41. Representative modeling showing the effects of changing individual equilibrium constants within Model 2 (shown at the lower right).

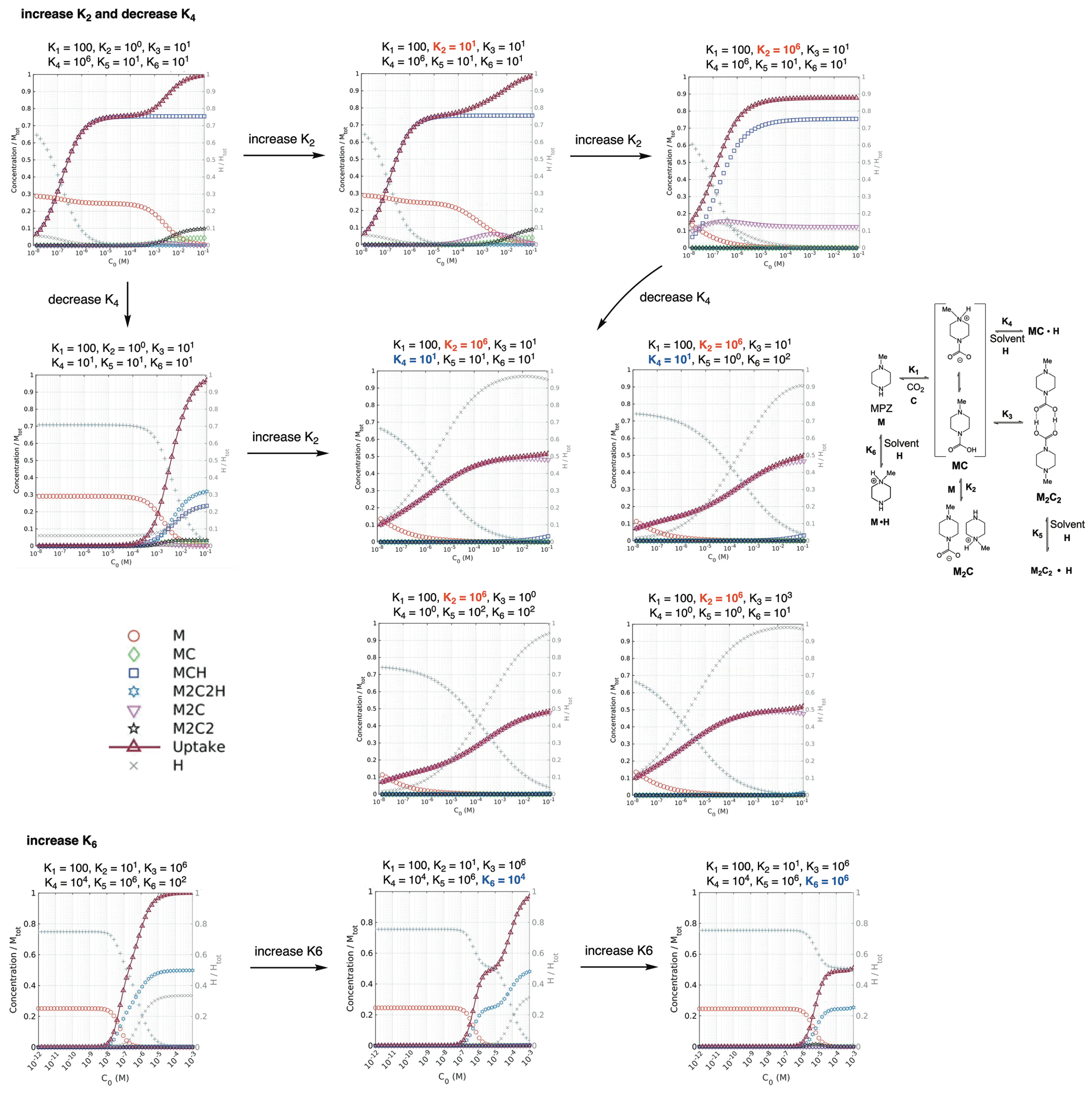

Figure S42. Representative modeling showing the effects of changing equilibrium constants within Model 3, in order to go from efficient two-step absorption to inefficient absorption.

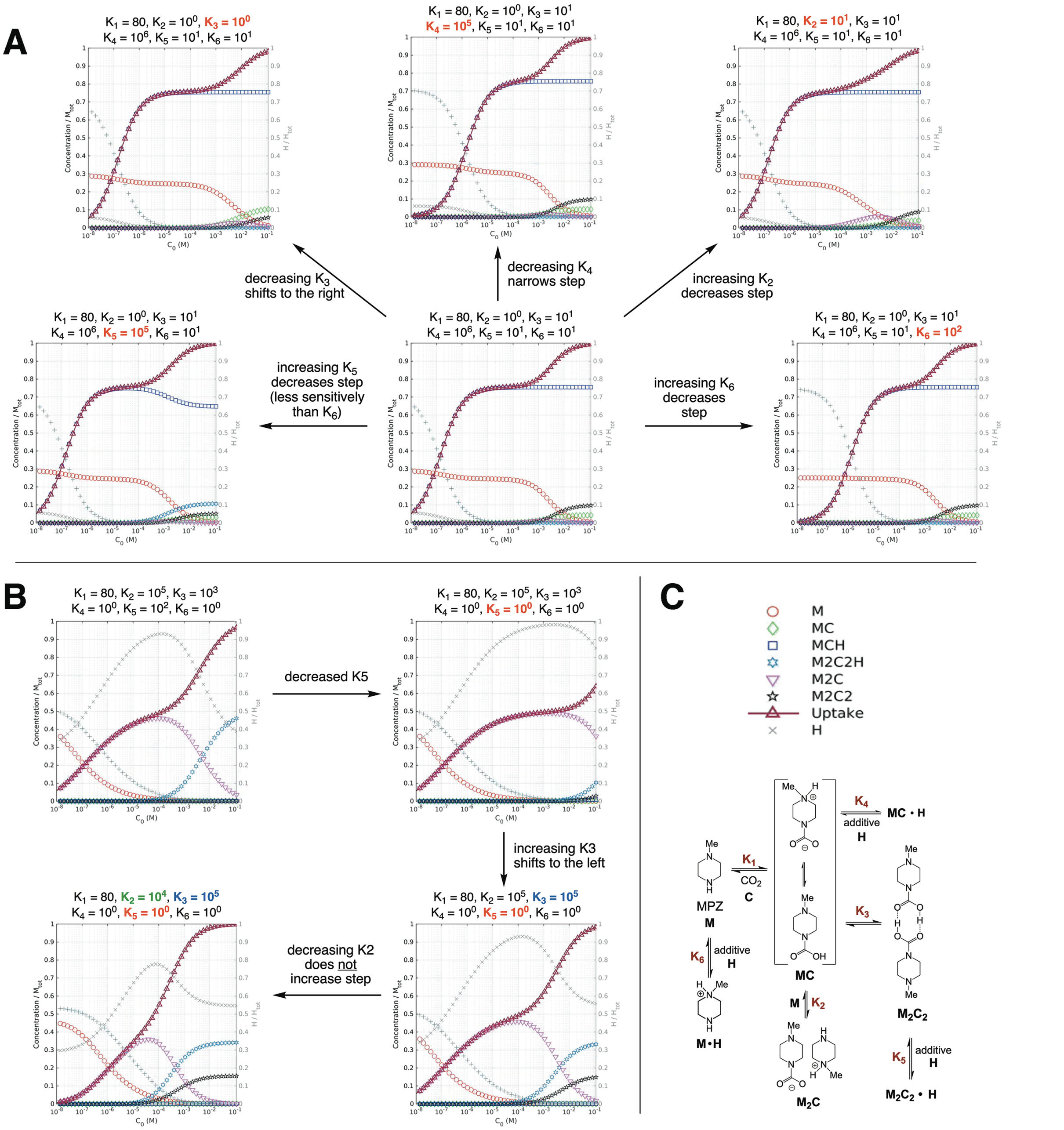

Figure S43. Representative modeling showing the effects of changing equilibrium constants within Model 3. (A) Equilibrium constant parameters that predict an isotherm step at relatively high absorbed CO_2/MPZ stoichiometry. (B) Equilibrium constant parameters that place the isotherm step at lower absorbed CO_2/MPZ ratio. (C) Key to the plots and model 3 equilibria definitions. Relatively low values of K_2 and K_3, coupled with relatively high values of K_4, place the step at a high value of adsorbed CO_2/MPZ, whereas high values of K_2 and K_3, paired with low values of K_4, result in the step occurring ratios at lower ratios of adsorbed CO_2 to base.

SI.8. Isotherm raw data tables

Table S3. Pressure-dependent CO_2 isotherm results plotted in Figure 2. Data values are in the form of X, Y (X = mmol CO_2 absorbed per gram solution; Y = mol CO_2 absorbed per mol MPZ).

	10wt.%MPZ/2'HAP			20wt.%MPZ/2'HAP			25wt.%MPZ/2'HAP	
Pressure (kPa)	X	Y	Pressure (kPa)	X	Y	Pressure (kPa)	X	Y
0.00	0.00	0.00	0.00	0.00	0.00	0.00	0.00	0.00
5.07	0.17	0.17	3.82	0.37	0.17	2.69	0.37	0.15
9.05	0.26	0.25	7.78	0.62	0.29	5.19	0.74	0.29
14.76	0.37	0.35	12.33	0.84	0.40	10.55	1.11	0.44
21.51	0.46	0.43	17.88	1.01	0.47	18.79	1.24	0.49
29.79	0.54	0.49	27.69	1.21	0.56	23.53	1.53	0.60
40.69	0.58	0.52	39.96	1.32	0.61	27.53	2.05	0.81
56.77	0.61	0.53	59.30	1.37	0.62	42.83	2.12	0.83
76.48	0.64	0.52	80.88	1.43	0.63	66.33	2.22	0.85
102.07	0.70	0.54	95.94	1.94	0.87	104.97	2.28	0.86
134.06	0.81	0.61	136.93	2.08	0.91	164.94	2.40	0.87
172.23	1.11	0.84	186.12	2.24	0.95			
218.34	1.26	0.92	231.47	2.31	0.95			
267.41	1.34	0.93	280.31	2.37	0.94			

	30wt.%MPZ/2'HAP			40wt.%MPZ/2'HAP			50wt.%MPZ/2'HAP	
Pressure (kPa)	X	Y	Pressure (kPa)	X	Y	Pressure (kPa)	X	Y
0.00	0.00	0.00	0.00	0.00	0.00	0.00	0.00	0.00
3.46	0.76	0.25	1.43	0.55	0.14	0.42	0.51	0.10
11.90	1.18	0.39	2.70	1.25	0.31	0.74	1.02	0.20
15.35	1.66	0.55	7.89	1.67	0.42	1.99	1.61	0.32
18.18	2.09	0.69	10.93	1.91	0.47	6.85	1.85	0.37
23.90	2.33	0.77	12.31	2.21	0.55	9.65	2.06	0.41
40.26	2.50	0.82	13.08	2.59	0.65	10.55	2.28	0.45
50.42	2.55	0.83	14.51	2.98	0.74	11.35	2.51	0.50
64.80	2.60	0.84	20.34	3.19	0.79	11.99	2.84	0.57
94.82	2.65	0.85	31.44	3.38	0.84	12.34	3.18	0.64
128.17	2.72	0.86	46.29	3.50	0.86	12.56	3.52	0.70
171.09	2.74	0.85	66.43	3.58	0.88	13.68	3.89	0.78
			86.97	3.71	0.91	16.55	4.16	0.83
			117.79	3.80	0.92	32.24	4.47	0.89
			163.78	3.88	0.93	50.82	4.55	0.90
						76.69	4.63	0.91
						132.77	4.70	0.92
						200.37	4.79	0.92

Table S4. Pressure-dependent CO_2 isotherm results plotted in Figure 3. Data values are in the form of X, Y (X = mmol CO_2 absorbed per gram solution; Y = mol CO_2 absorbed per mol MPZ).

	50wt.%MPZ/AP			50wt.%MPZ/2IPP			50wt.%MPZ/2CP	
Pressure (kPa)	X	Y	Pressure (kPa)	X	Y	Pressure (kPa)	X	Y
0.00	0.00	0.00	0.00	0.00	0.00	0.00	0.00	0.00
1.79	0.72	0.14	4.42	0.60	0.12	0.99	0.47	0.09
3.50	1.40	0.28	6.99	1.21	0.24	2.95	0.92	0.18
6.36	2.11	0.42	9.90	1.87	0.37	6.96	1.50	0.30
9.75	2.84	0.57	20.31	2.62	0.52	19.91	1.71	0.34
16.72	3.60	0.72	46.77	2.85	0.56	37.50	1.96	0.39
34.63	3.91	0.78	66.77	3.00	0.59	61.74	2.07	0.41
53.08	4.03	0.80	92.54	3.10	0.61	75.52	2.15	0.42
75.01	4.11	0.81	131.20	3.27	0.63	92.58	2.27	0.44
111.41	4.25	0.84	168.36	3.35	0.64			
159.03	4.34	0.85	198.94	3.46	0.66			
223.14	4.44	0.86						

Table S5. Pressure-dependent CO_2 isotherm results plotted in Figure 4. Data values are in the form of X, Y (X = mmol CO_2 absorbed per gram solution; Y = mol CO_2 absorbed per mol MPZ).

	50wt.%MPZ/3'HAP			50wt.%MPZ/4'HAP	
Pressure (kPa)	X	Y	Pressure (kPa)	X	Y
0.00	0.00	0.00	0.00	0.00	0.00
4.27	0.54	0.11	4.42	0.59	0.12
14.10	0.88	0.17	16.20	1.00	0.20
28.09	1.22	0.24	28.43	1.42	0.28
44.26	1.33	0.26	50.54	1.66	0.32
57.37	1.60	0.31	97.96	2.12	0.41
63.09	2.14	0.42	156.52	2.36	0.44
84.40	2.27	0.44	196.11	2.51	0.47
111.20	2.41	0.46	287.02	2.76	0.50
165.09	2.52	0.48			
226.37	2.58	0.48			

Table S6. Pressure-dependent CO_2 isotherm results plotted in Figure 5. Data values are in the form of X, Y (X = mmol CO_2 absorbed per gram solution; Y = mol CO_2 absorbed per mol MPZ).

	50wt.%MPZ/2'MAP			50wt.%MPZ/DIPB			50wt.%MPZ/Isopar	
Pressure (kPa)	X	Y	Pressure (kPa)	X	Y	Pressure (kPa)	X	Y
0.00	0.00	0.00	0.00	0.00	0.00	0.00	0.00	0.00
1.66	0.66	0.13	2.07	0.48	0.10	2.30	0.57	0.11
2.90	1.44	0.29	3.01	1.03	0.21	4.99	1.12	0.22
9.64	1.96	0.39	5.67	1.42	0.28	7.39	1.62	0.32
15.38	2.35	0.47	8.72	1.83	0.37	12.21	2.02	0.40
20.11	2.79	0.56	11.48	2.37	0.47	17.88	2.55	0.51
21.55	3.48	0.69	12.27	2.99	0.60	28.64	2.81	0.56
24.95	3.93	0.78	15.86	3.72	0.74	39.46	3.00	0.59
39.29	4.05	0.80	43.06	4.16	0.83	60.57	3.18	0.63
57.08	4.12	0.81	80.64	4.23	0.84	101.24	3.26	0.63
77.23	4.23	0.83	138.51	4.30	0.84	160.81	3.32	0.64
115.21	4.32	0.84	192.12	4.38	0.85	214.98	3.40	0.64
201.03	4.51	0.86						

Table S7. Solvent isotherms at 30℃.

	AP		2'HAP		2'MAP		DIPB
Pressure (kPa)	CO_2 loading (mmol CO_2/g solution)	Pressure (kPa)	CO_2 loading (mmol CO_2/g solution)	Pressure (kPa)	CO_2 loading (mmol CO_2/g solution)	Pressure (kPa)	CO_2 loading (mmol CO_2/g solution)
0.00	0.00	0.00	0.00	0.00	0.00	0.00	0.00
25.42	0.08	64.41	0.04	12.15	0.05	16.24	0.02
65.05	0.13	124.63	0.15	33.55	0.10	58.95	0.13
175.91	0.24	173.68	0.22	89.51	0.21	134.10	0.22
268.87	0.37	222.40	0.38	152.91	0.33	220.71	0.32
494.50	0.71	318.32	0.57	249.92	0.52	321.40	0.44
		413.60	0.74			434.70	0.59
		519.31	0.92				

Table S8. CO_2 isotherms of 30 and 40 wt.% MPZ in AP at 30℃

	30MPZ/AP		40MPZ/AP
Pressure (kPa)	CO_2 loading (mmol CO_2/g solution)	Pressure (kPa)	CO_2 loading (mmol CO_2/g solution)
0.00	0.00	0.00	0.00
2.80	0.35	3.41	0.66
6.56	0.96	5.46	1.36
12.13	1.64	8.27	2.12
28.57	2.24	12.89	2.88
58.49	2.49	27.05	3.36
96.47	2.58	57.69	3.50
145.43	2.64	79.42	3.59
210.89	2.73	116.68	3.65
		160.28	3.70

Table S9. CO_2 isotherms of 50 wt.% MPZ in 3'HAP and 4'HAP at 30℃

	50MPZ / 3'HAP run1		50MPZ / 3'HAP run2		50MPZ / 4'HAP run1		50MPZ / 4'HAP run2
Pressure (kPa)	CO_2 loading (mmol CO_2/g solution)	Pressure (kPa)	CO_2 loading (mmol CO_2/g solution)	Pressure (kPa)	CO_2 loading (mmol CO_2/g solution)	Pressure (kPa)	CO_2 loading (mmol CO_2/g solution)
0.00	0.00	0.00	0.00	0.00	0.00	0.00	0.00
4.27	0.54	6.02	0.52	4.42	0.59	4.01	0.42
14.10	0.88	18.01	0.85	16.20	1.00	13.61	0.95
28.09	1.22	31.05	1.09	28.43	1.42	28.49	1.41
44.26	1.33	47.40	1.26	50.54	1.66	44.33	1.91
57.37	1.60	58.65	1.79	97.96	2.12	83.02	2.21
63.09	2.14	73.04	2.24	156.52	2.36	147.94	2.34
84.40	2.27	92.37	2.44	196.11	2.51	225.72	2.57
111.19	2.41	124.12	2.56	287.01	2.76		
165.09	2.52	173.89	2.68				
226.36	2.58	225.97	2.73				

Table S10. CO_2 isotherms of MEA, 40 wt.% MEA/2'HAP and 50wt.% MP/2'HAP solutions at 30℃.

	MEA		40MEA / 2'HAP run1		40MEA / 2'HAP run2		50MP / 2'HAP
Pressure (kPa)	CO_2 loading (mmol CO_2/g solution)	Pressure (kPa)	CO_2 loading (mmol CO_2/g solution)	Pressure (kPa)	CO_2 loading (mmol CO_2/g solution)	Pressure (kPa)	CO_2 loading (mmol CO_2/g solution)
0.00	0.00	0.00	0.00	0.00	0.00	0.00	0.00
2.42	0.72	4.39	0.54	6.09	0.30	0.49	0.45
2.32	1.57	6.14	1.17	8.05	0.75	1.05	0.93
2.74	2.51	18.59	1.34	18.59	1.17	2.38	1.38
7.94	3.40	36.26	1.46	45.61	1.35	7.08	1.73
17.27	4.36	67.42	1.53	97.98	1.50	11.97	1.99
52.84	5.12	125.27	1.61	137.80	1.54	18.58	2.16
79.71	5.81			187.17	1.64	25.56	2.37
142.24	6.02					36.86	2.43
						52.21	2.50
						70.55	2.60
						105.27	2.66
						184.05	2.84

Table S11. Absorption capacity of MPZ in DIPB, 2'MAP and Isopar at 30℃ with 25% CO_2.

	Absorption capacity (mmol/g)	mol CO_2 / mol MPZ
50MPZ/DIPB run1	2.17	0.4347
50MPZ/DIPB run2	2.41	0.4828
50MPZ/DIPB run3	2.35	0.4708
30MPZ/DIPB run1	2.69	0.8981
30MPZ/DIPB run2	2.63	0.8781
50MPZ/2'MAP	4.31	0.8634
50MPZ/Isopar	1.67	0.3345
30MPZ/Isopar run1	1.16	0.3873
30MPZ/Isopar run2	1.3	0.4340
30MPZ/Isopar run3	1.34	0.4474

Table S12. Pressure-dependent CO_2 isotherm results plotted in Figure S3. Data values are in the form of X, Y (X = mmol CO_2 absorbed per gram solution; Y = mol CO_2 absorbed per mol MPZ).

10wt.%MPZ/2'HAP_run1			10wt.%MPZ/2'HAP_run2			10wt.%MPZ/2'HAP_run3		
Pressure (kPa)	X	Y	Pressure (kPa)	X	Y	Pressure (kPa)	X	Y
0	0.00	0.00	0.00	0.00	0.00	0.00	0.00	0.00
5.07	0.17	0.17	3.77	0.20	0.19	7.85	0.23	0.22
9.05	0.26	0.25	15.63	0.37	0.35	16.42	0.36	0.34
14.76	0.37	0.35	36.19	0.57	0.51	32.80	0.51	0.46
21.51	0.46	0.43	58.41	0.61	0.52	56.91	0.60	0.51
29.79	0.54	0.49	83.18	0.67	0.54	85.22	0.65	0.52
40.69	0.58	0.52	104.68	0.77	0.61	103.46	0.75	0.59
56.77	0.61	0.53	135.69	0.99	0.78	121.06	0.92	0.74
76.48	0.64	0.52	172.33	1.20	0.93	140.31	1.13	0.92
102.07	0.70	0.54	216.87	1.28	0.95	162.71	1.22	0.97
134.06	0.81	0.61	261.45	1.34	0.93	195.06	1.25	0.95
172.23	1.11	0.84	316.31	1.39	0.90	220.36	1.28	0.94
218.34	1.26	0.92						
267.41	1.34	0.93						

20wt.%MPZ/2'HAP_run1			20wt.%MPZ/2'HAP_run2			20wt.%MPZ/2'HAP_run3		
Pressure (kPa)	X	Y	Pressure (kPa)	X	Y	Pressure (kPa)	X	Y
0.00	0.00	0.00	0.00	0.00	0.00	0.00	0.00	0.00
3.82	0.37	0.17	5.84	0.40	0.19	4.34	0.31	0.15
7.78	0.62	0.29	12.62	0.71	0.35	11.72	0.62	0.30
12.33	0.84	0.40	25.94	1.08	0.52	20.39	0.88	0.43
17.88	1.01	0.47	52.10	1.34	0.64	28.39	1.03	0.50
27.69	1.21	0.56	81.76	1.49	0.69	37.41	1.17	0.56
39.96	1.32	0.61	99.82	1.87	0.87	52.97	1.24	0.58
59.30	1.37	0.62	117.49	2.04	0.94	77.87	1.36	0.63
80.88	1.43	0.63	142.74	2.08	0.94	88.19	1.76	0.82
95.94	1.94	0.87	187.44	2.13	0.94	110.71	1.99	0.92
136.93	2.08	0.91	226.37	2.18	0.93	158.78	2.12	0.95
186.12	2.24	0.95	270.41	2.20	0.92	218.29	2.19	0.95
231.47	2.31	0.95						
280.31	2.37	0.94						

25wt.%MPZ/2'HAP_run1			25wt.%MPZ/2'HAP_run2			25wt.%MPZ/2'HAP_run3		
Pressure (kPa)	X	Y	Pressure (kPa)	X	Y	Pressure (kPa)	X	Y
0.00	0.00	0.00	0.00	0.00	0.00	0.00	0.00	0.00
2.69	0.37	0.15	3.30	0.56	0.22	1.66	0.42	0.17
5.19	0.74	0.29	10.04	1.12	0.44	3.79	0.78	0.31
10.55	1.11	0.44	20.49	1.36	0.53	8.34	1.06	0.42
18.79	1.24	0.49	24.38	1.71	0.67	14.74	1.28	0.51
23.53	1.53	0.60	31.72	2.11	0.83	20.80	1.44	0.57
27.53	2.05	0.81	44.19	2.23	0.87	23.23	1.74	0.69
42.83	2.12	0.83	70.12	2.31	0.89	26.28	2.07	0.81
66.33	2.22	0.85	88.55	2.34	0.89	30.98	2.26	0.89
104.97	2.28	0.86	116.32	2.38	0.90	40.61	2.31	0.90
164.94	2.40	0.87	166.70	2.44	0.89	56.36	2.35	0.91
			228.12	2.51	0.89	80.96	2.39	0.92
						116.45	2.44	0.92
						165.24	2.50	0.92

30wt.%MPZ/2'HAP_run1			30wt.%MPZ/2'HAP_run2			30wt.%MPZ/2'HAP_run3		
Pressure (kPa)	X	Y	Pressure (kPa)	X	Y	Pressure (kPa)	X	Y
0.00	0.00	0.00	0.00	0.00	0.00	0.00	0.00	0.00
1.54	0.39	0.13	1.32	0.40	0.13	3.46	0.76	0.25
3.37	0.97	0.32	3.02	0.82	0.27	11.90	1.18	0.39
11.80	1.35	0.45	7.81	1.09	0.36	15.35	1.66	0.55
13.79	1.96	0.65	13.47	1.35	0.44	18.18	2.09	0.69
32.87	2.29	0.75	16.53	1.79	0.59	23.90	2.33	0.77
64.03	2.46	0.80	22.02	2.18	0.72	40.26	2.50	0.82
105.72	2.63	0.84	30.31	2.40	0.79	50.42	2.55	0.83
170.12	2.71	0.85	46.32	2.48	0.81	64.80	2.60	0.84
			59.24	2.54	0.83	94.82	2.65	0.85
			78.07	2.59	0.84	128.17	2.72	0.86
			132.52	2.69	0.85	171.09	2.74	0.85

40wt.%MPZ/2'HAP_run1			40wt.%MPZ/2'HAP_run2			40wt.%MPZ/2'HAP_run 3			40wt.%MPZ/2'HAP_run4		
Pressure (kPa)	X	Y	Pressure (kPa)	X	Y	Pressure (kPa)	X	Y	Pressure (kPa)	X	Y
0.00	0.00	0.00	0.00	0.00	0.00	0.00	0.00	0.00	0.00	0.00	0.00
1.05	0.30	0.07	3.21	0.84	0.21	1.43	0.55	0.14	1.13	0.53	0.13
1.35	0.77	0.19	4.31	1.39	0.35	2.70	1.25	0.31	2.04	1.07	0.27
3.09	1.32	0.33	8.73	1.86	0.46	7.89	1.67	0.42	5.98	1.52	0.38
8.54	1.74	0.43	9.57	2.38	0.59	10.93	1.91	0.47	10.61	2.07	0.51
9.24	2.34	0.58	10.52	2.96	0.74	12.31	2.21	0.55	11.58	2.77	0.69
9.67	2.82	0.70	13.38	3.17	0.79	13.08	2.59	0.65	25.27	3.31	0.82
24.01	3.26	0.81	21.61	3.35	0.83	14.51	2.98	0.74	54.91	3.57	0.88
51.84	3.39	0.84	36.40	3.49	0.87	20.34	3.19	0.79	97.04	3.73	0.91
97.43	3.63	0.88	62.02	3.60	0.88	31.44	3.38	0.84	142.73	3.82	0.92
136.50	3.69	0.89	90.58	3.71	0.91	46.29	3.50	0.86			
183.95	3.76	0.89	114.05	3.74	0.91	66.43	3.58	0.88			
						86.97	3.71	0.91			
						117.79	3.80	0.92			
						163.78	3.88	0.93			

50wt.%MPZ/2'HAP_run1			50wt.%MPZ/2'HAP_run2			50wt.%MPZ/2'HAP_run3		
Pressure (kPa)	X	Y	Pressure (kPa)	X	Y	Pressure (kPa)	X	Y
0.00	0.00	0.00	0.00	0.00	0.00	0.00	0.00	0.00
0.20	0.61	0.12	0.46	0.47	0.09	0.42	0.51	0.10
0.85	1.17	0.23	0.54	1.00	0.20	0.74	1.02	0.20
5.00	1.73	0.35	1.81	1.41	0.28	1.99	1.61	0.32
8.82	2.31	0.46	4.92	1.78	0.36	6.85	1.85	0.37
9.76	3.13	0.63	9.11	2.24	0.45	9.65	2.06	0.41
15.82	3.86	0.77	9.81	2.79	0.56	10.55	2.28	0.45
39.36	4.22	0.84	10.43	3.55	0.71	11.35	2.51	0.50
64.23	4.32	0.86	17.68	4.05	0.81	11.99	2.84	0.57
93.64	4.39	0.86	42.50	4.22	0.84	12.34	3.18	0.64
			64.95	4.32	0.85	12.56	3.52	0.70
			110.04	4.38	0.86	13.68	3.89	0.78
						16.55	4.16	0.83
						32.24	4.47	0.89
						50.82	4.55	0.90
						76.69	4.63	0.91
						132.77	4.70	0.92
						200.37	4.79	0.92

References

1. Hasell, T.; Cul aw, J. L.; Chong, S. Y.; Schmidtmann, M.; Little, M. A.; Jelfs, K. E.; Pyzer-Knapp, E. O.; Shepherd, H.; Adams, D. J.; Day, G. M.; Cooper, A. I., Controlling the Crystallization of Porous Organic Cages: Molecular Analogs of Isoreticular Frameworks Using Shape-Specific Directing Solvents. *Journal of the American Chemical Society* **2014,** *136* (4), 1438-1448.
2. Li, H.; Le Moullec, Y.; Lu, J.; Chen, J.; Valle Marcos, J. C.; Chen, G.; Chopin, F., CO2 solubility measurement and thermodynamic modeling for 1-methylpiperazine/water/CO2. *Fluid Phase Equilibria* **2015,** *394*, 118-128.
3. Borne, I.; Saigal, K.; Jones, C. W.; Lively, R. P., Thermodynamic Evidence for Type II Porous Liquids. *Industrial & Engineering Chemistry Research* **2023,** *62* (29), 11689-11696.
4. Mitra, A.; Seaton, P. J.; Ali Assarpour, R.; Williamson, T., Unprecedented concentration dependent chemical shift variation in1H-NMR studies: A caveat in the investigations of molecular recognition and structure elucidation. *Tetrahedron* **1998,** *54* (51), 15489-15498.
5. Saltelli, A.; Tarantola, S.; Chan, K.-S., A quantitative model-independent method for global sensitivity analysis of model output. *Technometrics* **1999,** *41* (1), 39-56.
6. Dvoriashyna, M.; Bentley-Ford, M.; Yu, J.; Chatterjee, S.; Pardue, M. T.; Kane, M. A.; Repetto, R.; Ethier, C. R., All-trans retinoic acid and fluid transport in myopigenesis. *bioRxiv* **2025**.
7. Darunte, L. A.; Sen, T.; Bhawanani, C.; Walton, K. S.; Sholl, D. S.; Realff, M. J.; Jones, C. W., Moving beyond adsorption capacity in design of adsorbents for CO2 capture from ultradilute feeds: kinetics of CO2 adsorption in materials with stepped isotherms. *Industrial & Engineering Chemistry Research* **2018,** *58* (1), 366-377.
8. Ding, X.; Chen, H.; Li, J.; Zhou, T., Comparative techno-economic analysis of CO2 capture processes using blended amines. *Carbon Capture Science & Technology* **2023,** *9*, 100136.
